# Isolation and genome sequencing of four Arctic marine *Psychrobacter* strains exhibiting multicopper oxidase activity

**DOI:** 10.1186/s12864-016-2445-4

**Published:** 2016-02-16

**Authors:** Morteza Shojaei Moghadam, Andreas Albersmeier, Anika Winkler, Lorenzo Cimmino, Kjersti Rise, Martin Frank Hohmann-Marriott, Jörn Kalinowski, Christian Rückert, Alexander Wentzel, Rahmi Lale

**Affiliations:** Department of Biotechnology, PhotoSynLab, Faculty of Natural Sciences and Technology, NTNU Norwegian University of Science and Technology, N-7491 Trondheim, Norway; Technology Platform Genomics, Center for Biotechnology (CeBiTec), Bielefeld University, Sequenz 1, D-33615 Bielefeld, Germany; Current address: Sinskey Lab, Department of Biology, Massachusetts Institute of Technology, Cambridge, MA 02139 USA; Department of Biotechnology, SINTEF Materials and Chemistry, N-7465 Trondheim, Norway

**Keywords:** *Psychrobacter*, Multicopper oxidase, Laccase, High-throughput screening, Genome sequencing, Heterologous expression, *Escherichia coli*

## Abstract

**Background:**

Marine cold-temperature environments are an invaluable source of psychrophilic microbial life for new biodiscoveries. An Arctic marine bacterial strain collection was established consisting of 1448 individual isolates originating from biota, water and sediment samples taken at a various depth in the Barents Sea, North of mainland Norway, with an all year round seawater temperature of 4 °C. The entire collection was subjected to high-throughput screening for detection of extracellular laccase activity with guaiacol as a substrate.

**Results:**

In total, 13 laccase-positive isolates were identified, all belonging to the *Psychrobacter* genus. From the most diverse four strains, based on 16S rRNA gene sequence analysis, all originating from the same *Botryllus* sp. colonial ascidian tunicate sample, genomic DNA was isolated and genome sequenced using a combined approach of whole genome shotgun and 8 kb mate-pair library sequencing on an Illumina MiSeq platform. The genomes were assembled and revealed genome sizes between 3.29 and 3.52 Mbp with an average G + C content of around 42 %, with one to seven plasmids present in the four strains. Bioinformatics based genome mining was performed to describe the metabolic potential of these four strains and to identify gene candidates potentially responsible for the observed laccase-positive phenotype. Up to two different laccase-like multicopper oxidase (LMCO) encoding gene candidates were identified in each of the four strains. Heterologous expression of P11F6-LMCO and P11G5-LMCO2 in *Escherichia coli* BL21 (DE3) resulted in recombinant proteins exhibiting 2,2'-azino-bis-3-ethylbenzothiazoline-6-sulphonic acid (ABTS) and guaiacol oxidizing activity.

**Conclusions:**

Thirteen *Psychrobacter* species with laccase-positive phenotype were isolated from a collection of Arctic marine bacteria. Four of the isolates were genome sequenced. The overall genome features were similar to other publicly available *Psychrobacter* genome sequences except for P11G5 harboring seven plasmids. However, there were differences at the pathway level as genes associated with degradation of phenolic compounds, nicotine, phenylalanine, styrene, ethylbenzene, and ethanolamine were detected only in the *Psychrobacter* strains reported in this study while they were absent among the other publicly available *Psychrobacter* genomes. In addition, six gene candidates were identified by genome mining and shown to possess T1, T2 and T3 copper binding sites as the main signature of the three-domain laccases. P11F6-LMCO and P11G5-LMCO2 were recombinantly expressed and shown to be active when ABTS and guaiacol were used as substrates.

**Electronic supplementary material:**

The online version of this article (doi:10.1186/s12864-016-2445-4) contains supplementary material, which is available to authorized users.

## Background

With 70 % of the Earth’s surface covered by oceans and 90 % of their water having temperatures of maximum 5 °C [1], the oceans represent a major resource for new bio-discoveries of psychrophilic and psychrotolerant species. Increasing efforts in recent years have aimed at describing microbial biodiversity in a variety of marine cold-temperature environments, including large-scale approaches. These studies led to unprecedented insight into the biodiversity of these environments, largely promoted by the significant technological developments in standardized sampling and next-generation sequencing of metagenome and metatranscriptome [2]. Psychrophilic microorganisms living at cold temperatures must cope with reduced rates of biochemical and physical processes in response to the low molecular kinetic energy of their environment. However, these organisms, through evolutionary processes, developed metabolic capabilities that enable them to survive and multiply at low temperatures [3, 4]. Cold-active biocatalysts from psychrophilic microbial strains are attractive with respect to applications that profit from low-temperature reaction conditions, for example due to thermal instability of products and reactants, like in the food processing industry, or the need to efficiently inactivate the biocatalysts during downstream processes, as exemplified by enzymes for bio-chemical synthesis or molecular research [5–8]. Advantages of such enzymes are their often high rates of catalytic activity at low temperature, low temperature optima and less resistance to thermal inactivation as compared to their mesophilic counterparts. Psychrophilic microbial strains with suitable biochemical capabilities may also be used in bio-remediation of cold-temperature environments polluted with recalcitrant chemicals, like aromatic or aliphatic hydrocarbons from crude oil [9].

Laccases (EC 1.10.3.2, p-diphenol:dioxygen oxidoreductase) are copper-containing oxidases that catalyze the monoelectronic oxidation of various aromatic substances at the expense of molecular oxygen, producing water as the sole by-product [10]. Belonging to the blue-copper family of oxidases, they have their main role in nature both in construction and de-construction of complex aromatic polymers, in particular lignin, and thus many different laccases have so far been described from plants, insects, fungi and bacteria [11]. In combination with electron transfer mediators, forming laccase-mediator systems (LMS), their oxidation capability can be even expanded beyond their natural phenolic substrates [12]. Due to considerable improvements in enzyme production and purification methods, today both laccases and LMS represent ideal biocatalysts for a plethora of biotechnological oxidation processes. At present, laccases or LMS are used as industrial enzymes in diverse applications: for delignification of lignocellulosic fibers and bleaching in pulp and paper industry [13, 14]; and in the de-colorization of dyes in the textile and printing industries [15]. They have also the potential to gain a broader role in chemical synthesis [10], as well as in bio-remediation of industrial waste waters and soils contaminated with e.g., dyes [16], polyaromatic hydrocarbons [17], or extra heavy crude oil [18]. Laccase functionality based on the conversion of classical laccase substrates such as 2,2'-azino-bis-3-ethylbenzothiazoline-6-sulphonic acid (ABTS), 2,6-dimethoxyphenol (2,6-DMP) and guaiacol has lately been shown to be exhibited by other proteins and enzymes not specifically assigned as laccases based on Enzyme Commission (EC) nomenclature, including copper resistance protein [19] and endospore coat protein [20]. As for the laccases themselves, the spectrum of substrates these enzymes convert varies [21], hence rendering the assignment of an enzyme as a laccase rather subjective.

Fungal laccases have been extensively studied [22–24]. Despite generally high level of enzymatic activity shown by fungal laccases they do not possess desired features exhibited by their bacterial counterparts such as higher stability over wide ranges of pH and temperature [25]. Therefore, there is a growing interest in the search of new bacterial laccases. Bacterial laccases are produced by phylogenetically diverse bacteria with various biological functions [25–27]. However, despite the recent advances in the discovery and characterization of laccases and laccase like proteins from bacterial sources [26–29], our knowledge is still limited regarding cold active bacterial laccases, while the importance of cold active enzymes both in academy and industry is well documented [6].

In this study, we describe the discovery and genome sequencing of four new *Psychrobacter* strains originating from the Barents Sea that exhibit laccase activity and heterologous expression of two of these enzymes in *Escherichia coli* BL21 (DE3). These strains and/or their aromatic compounds oxidizing enzymes may have broad application potentials as cold-active biocatalysts.

## Results

### Building and characterizing a culture collection of bacterial isolates from an Arctic marine environment

In collaboration with UiT, the Arctic University of Norway, Tromsø, Norway, biota, water and sediments were sampled between May 14th and 26th, 2009, on a research cruise of R/V Jan Mayen in North of Norway within the Arctic Circle. Sampling was done in ten different locations, spanning a region between and around the Svalbard archipelago and the Bear Island in the Barents Sea. The collection led to the establishment of a library of 1448 single bacterial isolates originating from biota (773), sediments (418), and water (257) samples. The strain library consists of, at least, 31 genera based on 16S rRNA gene sequences of 550 isolates, including *Algibacter*, *Aliivibrio*, *Alteromonas*, *Bacillus*, *Bizionia*, *Cellulophaga*, *Cobetia*, *Colwellia*, *Cytophaga*, *Flavobacterium*, *Halomonas*, *Lacinutrix*, *Leeuwenhoekiella*, *Marinobacterium*, *Marinomonas*, *Moritella*, *Neptunomonas*, *Olleya*, *Photobacterium*, *Planococcus*, *Polaribacter*, *Pseudoalteromonas*, *Pseudomonas*, *Psychrobacter*, *Psychromonas*, *Psychroserpens*, *Shewanella*, *Sulfitobacter*, *Tenacibaculum*, *Thalassomonas*, and *Vibrio* species.

### Screening and identification of strains showing laccase activity

**Fig. 1:**
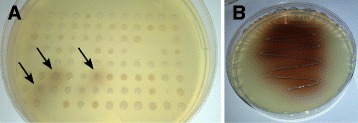
**a** A representative plate from primary high-throughput screening with three potentially laccase-positive hits indicated with arrows. **b** A representative plate from the laccase-activity confirmation step

The entire strain collection of 1448 individual bacterial isolates, arrayed in 96-well plates, was screened for isolates exhibiting extracellular laccase activity, and 13 of them scored positive based on the formation of brown zones around the colonies after 24 and 48 h of incubation at room temperature (20–23 °C). The color formation was observed after 24 h (Fig. 1a). However, the incubation time was prolonged to 48 h to capture the isolates that were slower in color formation due to either slower growth or reaction rates. Positive producers were re-streaked on modified marine agar plates from glycerol stocks and five well separated single colonies from each potential producer were picked and grown on screening plates (Fig. 1b). One colony from each producing isolate, confirmed for the laccase-positive phenotype, was chosen for further study.

**Fig. 2:**
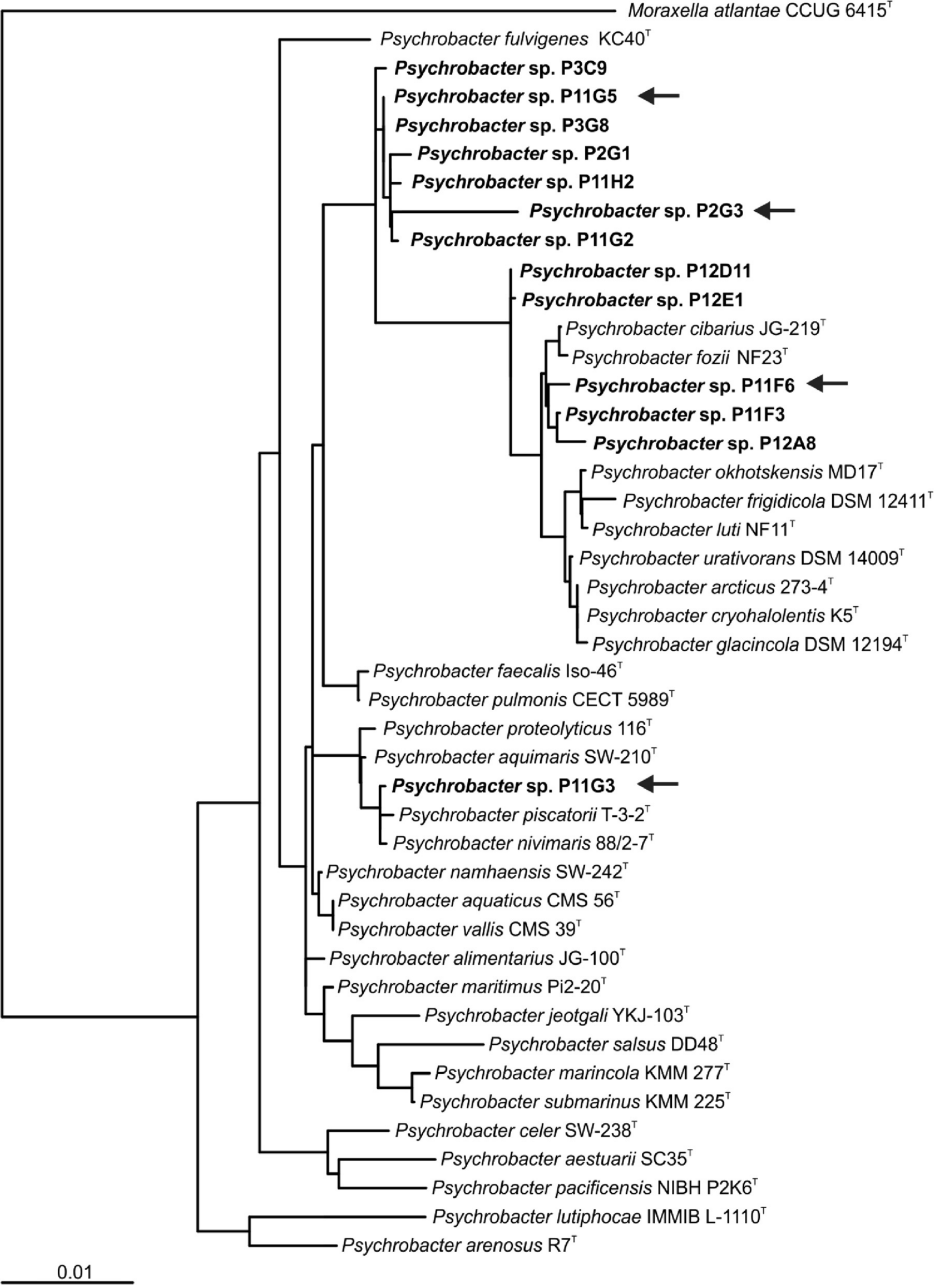
Phylogenetic tree of laccase-positive *Psychrobacter* species based on 16S rRNA gene sequence similarity. *Psychrobacter* strains with laccase-positive phenotype are indicated in bold font. *Psychrobacter* type strains were used as references, with *Moraxella atlantae* as an outgroup. The four strains selected for genome sequence determination are indicated with arrows

All 13 laccase-positive strains were identified as members of the genus *Psychrobacter* based on the 16S rRNA gene sequence analysis. The aligned 16S rRNA gene sequences were used to generate a phylogenetic tree by the Weighbor weighted neighbor joining method (Fig. 2). The four distantly related strains P11F6, P2G3, P11G3 and P11G5 were selected for genome sequencing.

### Genome features, annotation and comparative genomics

Manual *in silico* assembly of the initial Newbler assemblies resulted in one scaffold per replicon for all four genomes; P11G5 and P2G3 genomes completed, and two gaps in P11F6 and P11G3 chromosomes. The genomes of all four strains are almost identical in size and share a similar G + C content (Table 1), however display no significant average nucleotide identity (ANI) [30, 31], except for strains P2G3 and P11G5 which likely belong to the same novel species (97.22 % ANI). For the strain P11F6, high ANI values to *Psychrobacter* sp. JCM 18902 (95.77 % ANI) and *Psychrobacter* sp. JCM 18903 (95.65 % ANI) were found, while strain P11G3 shows significant similarity to *Psychrobacter* sp. JCM 18900 (94.09 % ANI) and *Psychrobacter* sp. JCM 18901 (93.30 % ANI). This fits well with P11F6 and P11G3 strains described as closely related to *P. cibarius* (strains JCM 18902 and JCM 18903) and *P. nivimaris* (strains JCM 18900 and JCM 18901) [32], respectively, thus validating the results for the 16S rRNA gene based classification.

**Table 1:** Genome features of the four *Psychrobacter* strains and other publicly available *Psychrobacter* genomes

Name	Ecosystem	Genome Size (bp)	GC (%)	CDS^a^ Count	HTG^b^ %	rDNA operon	tRNA Count	No. of Plasmid	Sequencing Method	Accession ID
*Psychrobacter* sp. 1501	Host-associated, Circulatory system	3,050,061	41	2660	6.45	1	46	unknown	454	GenBank: AFHU01000000
*Psychrobacter* sp. P11F6	Host-associated, Tunicates ascidians, Marine, Arctic	3,517,228	43	2871	2.58	6	53	1	Illumina MiSeq	This study
*Psychrobacter* sp. P11G3	Host-associated, Tunicates ascidians, Marine, Arctic	3,285,555	43	2650	2.62	5	50	4	Illumina MiSeq	This study
*Psychrobacter* sp. P11G5	Host-associated, Tunicates ascidians, Marine, Arctic	3,519,382	42	2859	3.66	4	48	7	Illumina MiSeq	This study
*Psychrobacter* sp. P2G3	Host-associated, Tunicates ascidians, Marine, Arctic	3,366,800	42	2743	4.1	4	48	3	Illumina MiSeq	This study
*P. aquaticus* CMS 56	Antarctica	3,216,409	43	2814	2.97	1	43	unknown	454	IMG:37750
*P. pacificensis* DSM 23406	Seawater, Japan	3,062,581	44	2666	1.69	2	40	unknown	Illumina HiSeq 2500	IMG:55227
*P. arcticus* 273-4	Terrestrial, Siberian permafrost	2,650,701	43	2147	6.46	4	49	unknown	Sanger	GenBank: CP000082
*P. cryohalolentis* K5	Aquatic, Siberian permafrost	3,101,097	42	2515	8.83	4	48	1	Sanger	GenBank: CP000323-4
*Psychrobacter* sp. LV10R520-6	Lake Visa, Antarctica	3,218,993	43	2666	4.35	4	49	unknown	PacBio RS	IMG:62179
*P. muriicola* 2pS	Arctic cryopeg	3,384,701	43	2820	1.9	6	53	unknown	Illumina HiSeq 2000, HiSeq 2500, PacBio RS	IMG:43211
*P. lutiphocae* DSM 21542	Host-associated, digestive system	3,176,011	41	2630	8.46	1	44	unknown	Illumina HiSeq 2000, HiSeq 2500	IMG:12623
*Psychrobacter* sp. PRwf-1	Host-associated, Skin, Off the coast, northeastern Puerto Rico.	2,995,049	45	2402	4.64	5	57	2	Sanger	GenBank: CP000713-5
*P. phenylpyruvicus* DSM 7000	Host-associated, Circulatory system	3,099,946	42	2622	-	2	45	unknown	Illumina HiSeq 2000, HiSeq 2500	IMG:35831
*Psychrobacter* sp. G	King george i land, Antarctica	3,113,999	42	2622	1.64	4	48	3	454 GS-FLX, Illumina	IMG:26029
*Psychrobacter* sp. UKMCC_SWTGB2	Host-associated, Tinggi island, south China sea	3,384,169	43	3054	14.6	1	31	unknown	Solexa	IMG:744
*Psychrobacter* sp. PAMC 21119	Terrestrial,Antarctica	3,510,716	43	2865	5.43	4	47	unknown	454-GS-FLX-Titanium, Illumina GAiix	IMG:24312
*Psychrobacter* sp. TB67	Host associated, Sponge, Antarctic	3,585,631	43	3021	-	6	52	unknown	Illumina HiSeq	IMG:43312
*Psychrobacter* sp. AC24	Host associated, Sponge, Antarctic	3,574,524	43	2999	-	6	52	unknown	Illumina HiSeq	IMG:39856
*Psychrobacter* sp. TB47	Host associated, Sponge, Antarctic	3,544,180	43	3134	-	2	53	unknown	Illumina HiSeq	IMG:43267
*Psychrobacter* sp. TB15	Host associated, Sponge, Antarctic	3,066,842	45	2574	-	2	45	unknown	Illumina HiSeq	IMG:39779
*Psychrobacter* sp. TB2	Host associated, Sponge, Antarctic	3,033,234	45	2544	-	2	45	unknown	Illumina HiSeq	IMG:39800
*Psychrobacter* sp. JCM 18900	Host associated, Frozen marine animals	3,272,645	43	2893	3.78	1	37	unknown	Ion Torrent PGM	IMG:38002
*Psychrobacter* sp. JCM 18903	Host associated, Frozen marine animals	3,427,960	43	3004	2	1	42	unknown	Ion Torrent PGM	IMG:43060
*Psychrobacter* sp. JCM 18901	Host associated, Frozen marine animals	3,145,827	43	2875	2.67	1	41	unknown	Ion Torrent PGM	IMG:43027
*Psychrobacter* sp. JCM 18902	Host associated, Frozen marine animals	3,274,327	43	2798	1.3	1	42	unknown	Ion Torrent PGM	IMG:43050

^a^CDS: Coding DNA sequence; ^b^HTG: Horizontally transferred gene

An interesting feature of the four genomes is the localization of the rRNA gene operons: all of the strains have two operons that are surrounded by CDS, while the remaining two, three and four operons (for strains P2G3-P11G5, P11G3 and P11F6, respectively) are clustered together. In all cases, two tRNAs are located between the 16S and 23S rRNA encoding genes.

Another point of variability among the four isolates is the number and the size of the plasmids they harbor: strain P11F6 contains one large plasmid (47.8 kbp); P2G3 contains three plasmids (23.9 kbp; 11.3 kbp, and 9.7 kbp); P11G3 has four small plasmids (8.5 kbp, 6.9 kbp, 5.6 kbp, and 5.5 kbp); and P11G5 harbors seven plasmids (41.0 kbp, 14.3 kbp, 13.6 kbp, 9.2 kbp, 6.0 kbp, 5.8 kbp, and 5.5 kbp).

The genome features were also compared to the other 22 *Psychrobacter* genomes that were publicly available as of December 2015 (Table 1), and shown to be similar to the other previously reported sequences except high number of plasmids in P11G5.

Pairwise genome alignments were performed for the four studied strains [see Additional file 1]. In agreement with ANI values for P2G3 and P11G5 (97.22 %), dot plot analysis for the two chromosomal DNA sequences [see Additional file 1: Figure S1C] confirm that they likely belong to the same species as they show very limited level of discontinuity in the main diagonal which could be explained by very few independent evolutionary events introducing insertions/deletions (indels). Also strains P11F6 and P11G3 show high level of similarity [see Additional file 1: Figure S1D]. In addition to indels observed throughout the sequences of the two strains, inverted sequence repeats were observed at the middle of the main diagonal which could be an indication of transposable elements. In other cases, significant variation was observed including indels, as well as transposable elements at the middle of the sequences, while the main diagonal remained relatively more consistent at both ends (see Additional file 1: Figure S1A, S1B, S1E and S1F).

**Fig. 3:**
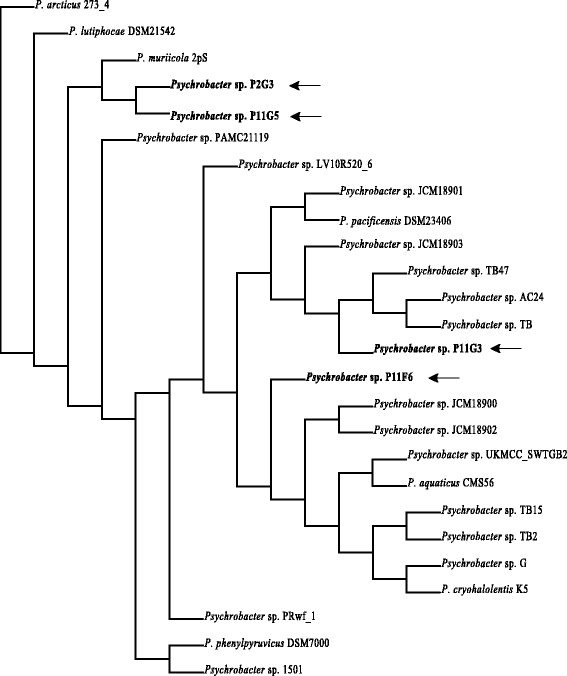
Hierarchical genome clustering of *Psychrobacter* species based on KEGG pathways. The four genome sequenced *Psychrobacter* species in this study are indicated in bold font and with arrows

**Fig. 4:**
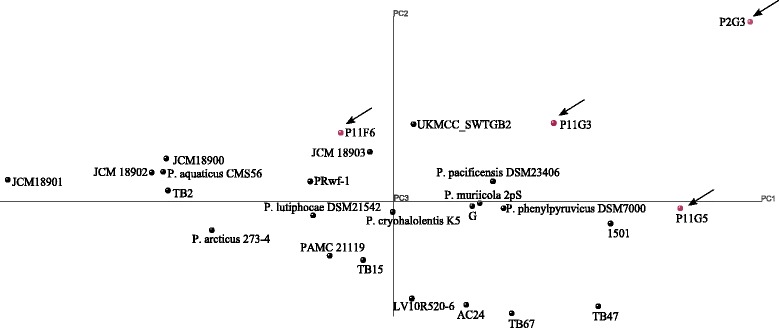
Genome clustering of *Psychrobacter* species based on KEGG pathways by principal component analysis (PCA). The four genome sequenced *Psychrobacter* species in this study are indicated with red spheres and arrows

In order to compare the metabolic profiles of the genomes, principal component analysis (PCA) and hierarchical clustering were performed based on KEGG pathways. According to hierarchical clustering, P11F6 and P11G3 were not clustered with any other species, while P11G5 and P2G3 were clustered together (Fig. 3). In PCA, however, P2G3 appeard as an outlier and clearly distinguished from P11G5 (Fig. 4). In order to get an insight as to which metabolic pathway distinguishes the studied genomes, not only from each other but also from the other previously reported *Psychrobacter* genomes, all genes were assigned to KEGG Orthology (KO) numbers and subsequently used to generate a heat map [see Additional file 2: Figure S2]. It should be noted that 36–50 % of the genes could not be connected to any KO. Interestingly, *Psychrobacter* sp. P11F6, P2G3 and P11G5 were the only strains possessing phenol hydroxylases (K16249, K16246, K16245, K16244, K16243 and K16242) that catalyzes the first step of phenol degradation pathway [33]. *Psychrobacter* sp. P11F6 and P2G3 were the only strains possessing maleate isomerase gene (K01799) which have been found in nicotine degrading bacteria catalyzing the conversion of maleate to fumarate which can subsequently be used to produce aspartic acid [34]. 4-hydroxy 2-oxovalerate aldolase (K01666), acetaldehyde dehydrogenase (K04073), and 3-phenylpropionate/trans-cinnamate dioxygenase ferredoxin reductase subunit (K00529, K05710) were detected only in *Psychrobacter* sp. P11F6. These enzymes are known to participate in the metabolism of aromatic amino acid phenylalanine along with other environmental pollutants with similar chemical structure such as styrene and ethylbenzene [35, 36]. *Psychrobacter* sp. P2G3 and P11G5 were the only strains possessing ethanolamine ammonia-lyase small subunit (K03736) and large subunit (K03735) which is an enzyme commonly found in Entrobacteriaceae family giving them the ability to utilize ethanolamine present in mammalian gastrointestinal tract as a source of carbone and nitrogen [37, 38]. Basic amino acid/polyamine antiporter-APA family (K03294) was only found in *Psychrobacter* sp. P11F6 which is an electron proton pump involved in extreme acidic resistance [39]. There were also certain genes that were found to be present in the four strains and only in a few of other previously reported genomes. For example, *Psychrobacter* sp. P2G3 and P11G5, together with *Psychrobacter* sp. UKMCC_SWTGB2 were the only strains with polysaccharide transporter (PST family; K03328) which potentially enables them to resist and survive in external environmental stress and host interaction settings [40]. Chemotaxis protein methyltransferase gene-*cheR* (K00575) was detected in *Psychrobacter* sp. P2G3, *Psychrobacter* sp. P11G5, *P. pacificensis* DSM 23406 and *P. cryohalolentis* K5. Interestingly, even though *cheY* and *cheX* were present in 16 of the analyzed strains, other chemotaxis and flagella-assembly associated genes were absent in all of the strains.

### Laccase-like gene candidates and sequence analysis

**Fig. 5:**
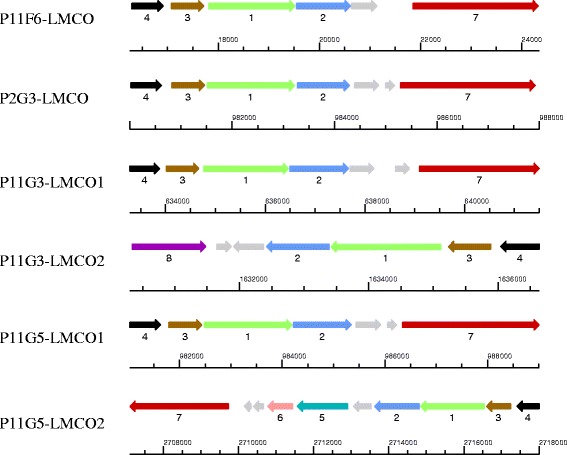
Gene organizations of gene candidates and their positions. 1: LMCO; 2: *copB*; 3: ATP-binding protein; 4: Cytochrome C; 5: amino acid permease; 6: Isoprenylcysteine carboxyl methyltransferase; 7: Copper-transporting P-type ATPase; 8: Oxidoreductase; Grey arrows represents hypothetical proteins

In total six laccase like multicopper oxidase (LMCO) genes were identified by genome mining (two each in P11G3 and P11G5, and one each in P11F6 and P2G3) possibly involved in the observed phenotype of laccase activity. These genes were annotated as copper-resistance protein (*copA*). The LMCO gene is located on a plasmid in the strain P11F6, while it is located on the chromosome of the other three strains. The two genes detected in P11G3 share 95 % similarity and located 1,000,347 bp apart from each other and oriented in opposite directions (Fig. 5). Similarly, the LMCO genes from P11G5 share 94 % similarity and are located 1,734,160 bp apart and also in opposite direction. In all six cases, the LMCO genes are followed by *copB* encoding a putative copper-resistance protein.

Table 2 summarizes the lengths, similarity analysis of the predicted candidate genes, and predicted residues forming the type 1 copper binding site. A complete list of amino acid residues predicted to form the substrate binding pocket is provided [see Additional file 3: Table S1]. The canonical three cupredoxin domains forming type 1 (T1), type 2 (T2) and type 3 (T3) copper binding sites of laccases were also detected in structural modeling analysis using Phyre2 [41]. In all cases, the T1 copper binding site was found to be composed of two histidines, one cysteine and one methionine residues (Table 2). The deduced amino acid sequence of all six LMCOs share 85–99 % similarity (Table 3). The signal sequences and the respective cleavage sites were detected in all sequences using the web based applications (see *Methods* for details).

**Table 2:** Sequence analysis of LMCO genes. Length of the genes (bp) and the encoded proteins (aa), amino acid residues forming T1 copper binding site, and the level of similarity to the already known proteins as analyzed by BLAST search are listed

Name	ORF (bp/aa)	T1 copper site	Amino acid similarity analysis	
			Description	Coverage/Similarity (%)
P11F6-LMCO	1707/568	H503 C551 H556 M561	multicopper oxidase [*Psychrobacter* sp. JCM 18902]	100/100
P2G3-LMCO	1704/567	H502 C550 H555 M560	CopA family copper resistance protein [*Psychrobacter* sp. PRwf-1]	100/95
P11G3-LMCO1	1701/566	H501 C549 H554 M559	copper resistance protein CopA [*Psychrobacter* sp. PAMC 21119]	100/96
P11G3-LMCO2	1701/566	H501 C549 H554 M559	copper resistance protein CopA [*Psychrobacter* sp. PAMC 21119]	100/94
P11G5-LMCO1	1704/567	H502 C550 H555 M560	CopA family copper resistance protein [*Psychrobacter* sp. PRwf-1]	100/95
P11G5-LMCO2	1698/565	H500 C548 H553 M558	MULTISPECIES: copper resistance protein CopA [*Psychrobacter*]	100/99

**Table 3:** The percentage of sequence similarity between amino acid sequences of the six discovered LMCOs

	P11F6-LMCO	P2G3-LMCO	P11G3-LMCO1	P11G3-LMCO2	P11G5-LMCO1	P11G5-LMCO2
P11F6-LMCO	N/A	86	88	87	87	85
P2G3-LMCO	86	N/A	90	91	99	94
P11G3-LMCO1	88	90	N/A	95	91	90
P11G3-LMCO2	87	91	95	N/A	92	91
P11G5-LMCO1	87	99	91	92	N/A	94
P11G5-LMCO2	85	94	90	91	94	N/A

### Enzymatic assay of P11F6-LMCO and P11G5-LMCO2

**Fig. 6:**
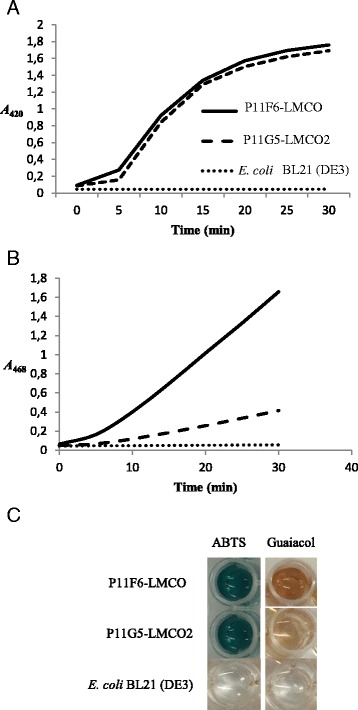
Enzymatic assay of P11F6-LMCO and P11G5-LMCO2 using ABTS and guaiacol as substrates. Increase in *A*_420_ and *A*_468_ when ABTS (**a**) and guaiacol (**b**) were used as the substrates, respectively. **c** Chromogenic products of ABTS and guaiacol oxidation by the studied LMCOs

The reaction mixture was prepared and absorbance was monitored at *A*_420_ and *A*_468_ for ABTS and guaiacol, respectively. The increase in absorbance was an indication of enzymatic activity (Fig. 6a and b). Moreover, the oxidized product of ABTS and guaiacol are green and brown, respectively, which for both enzymes could be confirmed visually (Fig. 6c).

## Discussion

Psychrophilic microorganisms are found in all three domains of life: bacteria, archaea, and eukarya [42]. However, in deep sea and cold temperature marine environments bacteria, and in particular Gamma-proteobacteria, are dominant. Many novel cultivable species of the genera *Colwellia*, *Moritella*, *Photobacterium*, *Psychromonas*, *Marinomonas* and *Shewanella* have been first described from cold environments [42]. It is therefore not surprising that the screening of the Arctic marine bacterial collection led to identification of strains belonging to the Gamma-proteobacteria exhibiting laccase activity.

Comparative genomics were performed on KEGG-based metabolic profile of the studied four strains against the other publicly available *Psychrobacter* genomes. This analysis revealed noteworthy differences as well as novel genes not being present in the previously reported *Psychrobacter* genomes. For instance, genes associated with metabolic pathways of phenol (in P11F6, P2G3 and P11G5), nicotine (in P11F6 and P2G3), phenylalanine/styrene/ethylbenzene (in P11F6), ethanolamine (in P2G3 and P11G5) degradation are detected only in the *Psychrobacter* species that are reported in this study. In addition, P11F6 is the only strain with basic amino acid/polyamine antiporter gene responsible for extreme acidic resistance. Although not unique to our strains, several other features relevant to survival in extreme conditions are also detected such as resistance to heavy metals, regulation of osmotic pressure and the ability to utilize different carbon, nitrogen, and sulfur sources. All of these features potentially enable the species to survive in harsh conditions. Noteworthy, neither flagella assembly genes nor complete set of genes encoding chemotaxis components could be found based on KO analysis, in agreement that members of the *Psychrobacter* genus are generally known to be non-motile [43].

The discovery of the first bacterial laccase [44] motivated many researchers to explore the bacterial domain for laccases. It is now well established that those from bacterial sources exhibit higher thermal- and pH stability compared to their fungal counterparts [28]. In this study, we sought to find LMCOs from marine cold adapted bacteria. There are a limited number of reports on laccase activity from bacteria living in cold environments, while the importance of cold-active enzymes is well known [6]. Recent reports support the extracellular laccase like activity from *Psychrobacter* genus [45], as well as psychrophilic/psychrotolerant bacterial species [46].

The bacterial collection used in this study was screened for extracellular laccase activity without supplementing the screening medium with additional copper. This suggests that the identified isolates produce enzyme(s) with laccase-like activity, most likely, extracellularly, if not taking up the substrate and pumping out the chromogenic product back to the extracellular medium. We tested enzymatic activity of cell free supernatant from cultures grown in the absence of substrate which led to no measurable laccase activity (data not shown). This observation may suggest that the production of LMCOs by the studied isolates is inducible. The enzymes did not rely solely on copper for the activity as there were other metal ions such as Mg^2+^ and Ca^2+^ present in the medium that could possibly serve as cofactors for laccases [47]. However, we observed more intense brown zone formations in the presence of 0.25 mM CuCl_2_ (data not shown).

The laccase-positive isolates were all identified as members of the genus *Psychrobacter*, while members belonging to known laccase-active genera such as *Shewanella* and *Pseudomonas* [27, 48] were also present in our collection. This may be explained by our screening system being designed to target only species producing LMCOs extracellularly and only those able to oxidize guaiacol as substrate. Bacterial LMCOs from *Gramella forsetii* KT0803, *Marivirga tractuosa* DSM4126 were shown to not accept guaiacol although they oxidize ABTS and syringaldazine [21].

Similar to the present study, the majority of known bacterial genes encoding proteins with laccase-like activity have been annotated as multicopper oxidase (MCO) or copper-resistance proteins (*copA)* [25]. The amino acid sequence analysis of the studied LMCOs revealed the presence of conserved T1, T2 and T3 copper binding sites of 3-domain laccases. The T1 site is typically composed of two histidines, one cysteine and one position which is usually variable [49]. The variable residue in the studied sequences was methionine which is in agreement with known bacterial LMCOs [49]. This position is replaced by leucine or phenylalanine in fungal laccases and has been argued to influence the oxidation potential [49]. Therefore, the studied LMCOs are likely among the low redox potential laccases with redox potential below 500 mV [50]. Notably, however, a bacterial laccase with redox potential as high as 638 mV was recently reported [51] which falls into the middle range of redox potentials (up to 700 mV) [50]. Although the redox potential of the T1 Cu site (where the substrate oxidation takes place) is the key parameter in the oxidation capacity of laccases, changes in amino acid residues constituting the substrate binding pocket can considerably change the *k*_cat_ and *K*_M_ values of laccases while maintaining the same redox potential of T1 Cu site [50, 52]. In spite of sharing high level of similarity at the amino acid sequence level, the studied LMCOs showed considerable differences in the amino acid residues forming their predicted substrate binding pocket [see Additional file 3: Table S1]. This suggests that the studied enzymes may exhibit different substrate preferences.

Detection of single and multiple laccase genes, both on plasmid and chromosomes, in this study is in agreement with the earlier report by Ausec and coworkers [27] in which the authors showed that some members of certain bacterial genera (e.g., *Rhodococcus*) possess multiple laccase genes in their genome and that the genes can be present both on a plasmid or a chromosome. The presence of multiple laccase genes may confer adaptive benefits with respect to certain life styles and environmental factors [53]. Prediction of signal peptides encoded by the studied gene candidates supports the extracellular laccase activity observed during high-throughput screening (HTS). The majority of putative laccase genes found from publicly available finished/draft genomes and metagenomic datasets encode signal peptide sequences suggesting that the gene products are exported from the cytoplasm [27].

Heterologous expression of P11F6-LMCO and P11G5-LMCO2, in *E.coli¸* confirmed the *in silico* based selection of the target enzymes showing laccase activity towards the phenolic substrate guaiacol, which was used during the initial HTS, as well as the bulky non-phenolic substrate ABTS. However, LMCO gene knock-out studies in the native strains are needed to confirm whether the identified candidate genes actually are responsible for the observed phenotype.

## Conclusions

The screening of a bacterial collection from marine Arctic environments was performed that led to identification of 13 *Psychrobacter* strains exhibiting extracellular laccase activity. Four of these strains were genome sequenced. Based on genome scale metabolic profile analysis, genes associated with degradation of phenolic compounds, nicotine, phenylalanine, styrene, ethylbenzene, and ethanolamine were detected only in the *Psychrobacter* strains reported in this study while they were absent in the other publicly available *Psychrobacter* genomes. In addition, six LMCO gene candidates were identified by genome mining, and were located both in plasmid and chromosomal DNA. Conserved domains for copper binding sites for three-domain laccases were also detected in all six sequences. The activity of heterologously expressed P11F6-LMCO and P11G5-LMCO2 were validated using both ABTS and guaiacol as substrate. Further studies are underway to evaluate the role of the identified LMCOs in the four laccase-positive *Psychrobacter* strains, as well as their full biochemical characterization with respect to their potential in various biotechnological applications.

## Methods

### Bacterial culture collection

Details about the generation of the Arctic marine strain collection, including in total 1448 bacterial isolates, are presented in the Results section. Of particular relevance for this study, a *Botryllus* sp. (colonial ascidian tunicate) sample was taken from a depth of about 20 m, with seawater temperature of 4.1 °C (69° 44,98704 N, 30° 25,21654 E). The specimen was washed with sterile sea water, and was homogenized, which was then used as inoculum. The homogenate was prepared and diluted in sterile sea water, and aliquots of the diluted homogenate were spread on IM8 agar plates (1 g/L malt extract, 1 g/L glycerol, 1 g/L glucose, 1 g/L peptone, 1 g/L yeast extract, 18 g/L agar, 16 g/L sea salt, [54]) and incubated at 4 °C for two weeks, until visible colonies appeared. Single colonies were picked and grown in liquid IM8 with 225 rpm agitation at 4 °C until enough cell growth had occurred. Samples were frozen in individual vials at −80 °C. Later on, single colonies were transferred to 96 deep-well plates for screening purposes.

### High-throughput screening for laccase activity

The bacterial collection in 96-well plate format was replicated in modified marine broth medium [15 g/L Difco marine broth, 5 g/L peptone, 150 mL/L artificial sea water (425 mM NaCl, 9 mM KCl, 9 mM CaCl_2_.2H_2_O, 26 mM MgSO_4_.7H_2_O, 23 mM MgCl_2_.6 H_2_O, pH 7.8)] grown overnight at 20 °C with 800 rpm orbital agitation. Cultures were then transferred and stamped to modified marine agar (modified marine broth containing 15 g/L bacteriological agar) plates (14 cm) supplemented with 0.01 % guaiacol (Sigma-Aldrich) by a 96-pin replicator. The plates were covered to protect from light and incubated at room temperature (20–23 °C) for two days. The plates were checked after 24 and 48 h. The colonies with visible brown color zones around were selected for further characterization.

### Isolation of total DNA and 16S rRNA gene sequence analysis

Total DNA from the producing isolates was purified using Wizard® Genomic DNA Purification Kit (Promega). 16S rRNA genes were amplified by PCR using primers 27f (5' AGAGTTTGATCMTGGCTCAG 3') and 1492r (5' TACGGYTACTTGTTACGACTT 3') [55]. The same primers were also used for direct sequencing of the PCR products. The similarity search of 16S rRNA gene sequences was performed by BLASTN [56]. A phylogenetic tree was then generated using the RDP database [57] with the 16S sequences of all *Psychrobacter* type strains as references and *Moraxella atlantae* as an outgroup.

### Genome sequencing, annotation and comparative genomics

Genomic DNA was sequenced in a combined approach using a whole genome shotgun and a mate pair library for each strain to be sequenced. The whole genome shotgun libraries were constructed with the TruSeq DNA PCR-Free library preparation kit (Illumina). 8 kb mate pair libraries were prepared with the Nextera mate pair sample preparation kit (Illumina) according to the gel-plus protocol. All libraries were sequenced in a paired-end run using the MiSeq reagent kit v3 (600 cycles) and the MiSeq desktop sequencer (Illumina). Reads from the WGS libraries were quality trimmed (at least 5 nt with > = Q30 at the 3' end). Reads from both libraries were assembled with the Roche GS *de novo* Assembler software (Newbler; release 2.8). The results for the initial assemblies are listed in Table 4. The gap closure step was facilitated by the Consed software (version 26) [58]. Gene prediction was performed with Prodigal [59], tRNAscan-SE [60], and RNAmmer [61] for CDS, tRNAs and rRNAs in general. Functional annotation was performed using the GenDB annotation platform [62]. ANI values were calculated using the implementation in EzBioCloud (http://www.ezbiocloud.net/).

**Table 4:** Statistics of the initial, automated assembly

		P2G3	P11F6	P11G3	P11G5
Aligned Reads		2,096,689	2,858,651	1,705,396	1,811,833
Assembled Bases		533,309,075	722,997,409	422,716,068	458,257,731
Contigs^a^		28/30/45	32/39/51	28/32/51	39/46/68
Scaffolds		6	6	8	8
Bases in scaffolds		3,345,653	3,468,794	3,246,680	3,495,250
Coverage		159.4	208.43	130.2	131.11
Gaps/MBase		8.4	9.2	8.6	11.2
G + C content [%]		41.79	42.73	42.76	41.81
Scaffold Size	Average	557,608	578,132	405,835	436,906
	Largest	3,289,362	2,649,068	2,388,248	3,395,360
Contig Size	Average	111,293	88,690	63,489	75,633
	Largest	378,865	441,017	655,512	487,469

^a^in scaffolds/large/all

The embedded comparative tools in RAST version 2.0 [63] and integrated microbial genome (IMG) [64] were used for comparative genome analyses using publicly available genome sequences of *Psychrobacter* strains listed in Table 1.

### Nucleotide sequence accession numbers

The Whole Genome Shotgun projects have been deposited at DDBJ/EMBL/GenBank under the accessions LJCE00000000 (strain P11F6) and LJCF00000000 (strain P11G3). The versions described in this paper are version LJCE01000000 and LJCF01000000, respectively. The complete annotated genome sequences (chromosomes and plasmids) have been deposited at DDBJ/EMBL/GenBank under the accession numbers CP012529-CP012532 (strain P2G3) and CP012533-CP012540 (strain P11G5).

### Genome mining and sequence analysis of gene candidates

Genome mining, to identify genes encoding for enzymes potentially involved in the observed laccase-positive phenotype of the four strains, was performed using amino acid sequences of putative laccases, identified by Ausec and colleagues [27], against the *Psychrobacter* genome sequences obtained in the present study by using BLAST [65]. In addition, *Psychrobacter* genome annotations were browsed for genes annotated as laccases, multicopper oxidases (MCOs) and copper-resistance proteins. The DNA sequences mutually detected by both approaches were chosen for further study. Structure modeling and analysis of conserved domains of the studied laccase like multicopper oxidases (LMCOs) was performed using Phyre2 [41] and the laccase from *Botrytis aclada* (3SQR) as the template. The deduced amino acid sequence encoded by the gene candidates were further analyzed for the presence of a signal peptide using the web based applications SignalP 4.0 server [66], PRED-TAT [67], Phobius [68], and Phyre2 [41]. The level of similarity shared between the sequences was evaluated using the multiple sequence alignment function of the software CloneManager 9 Professional edition (Scientific & Educational Software).

### Heterologous expression and activity validation of P11F6-LMCO and P11G5-LMCO2

In order to study the laccase activity of the LMCOs, P11F6-LMCO and P11G5-LMCO2 sharing the minimum homology (Table 3) were chosen as representatives. The region encoding mature protein, excluding the signal peptide predicted by SignalP, was heterologously expressed in Expresso™ T7 cloning and expression system (Lucigen). Cloning procedure was carried out according to manufacturer’s instructions. Expression was performed by inoculating 1 % of overnight-grown culture into 50 mL terrific broth medium [69] supplemented with 0.25 mM CuCl_2_ followed by incubation at 37 °C and 200 rpm until it reached OD600: ~1.0. The cultures were then induced by 0.1 mM isopropyl β-D-1-thiogalactopyranoside (IPTG) and incubated overnight at 10 °C at 200 rpm. The cells were harvested by centrifugation at 4000 × g for 15 min, and were resuspended in 5 mL lysis buffer (50 mM NaH_2_PO_4_, 300 mM NaCl, pH 8.0) followed by the addition of 1 mg/mL lysozyme and incubation on ice for 30 min. Cells were then disrupted by sonication. The lysate centrifuged at 10000 × g for 20 min. Crude extract was prepared by filtering supernatants through 0.22 μM sterile filters and transferring into a new tube. Crude extract obtained from untransformed *E. coli* BL21 (DE3) was used as a negative control.

The reaction mixture was prepared in a total volume of 200 μL containing 50 mM Tris–HCl pH 7.0, 0.25 mM CuCl_2_, and 10 μL of crude extract. Commonly used nonphenolic and phenolic laccase substrates ABTS (1 mM) and guaiacol (10 mM), respectively, were added into separate reaction mixture and the reactions were then monitored at 420 nm and 468 nm, respectively.

### Availability of supporting data

The datasets supporting the results of this article are included within the article and its Additional files.

Phylogenetic tree is available in the TreeBASE repository, [http://purl.org/phylo/treebase/phylows/study/TB2:S18786?x-accesscode=fa3db0ef767f25ea6936f6b6651efbd3&format=html].

## Additional files

Additional file 1: Figure S1. Dot plot analysis of chromosomal DNA sequences. A) P2G3 vs P11G3, B) P11F6 vs P2G3, C) P2G3 vs P11G5, D) P11F6 vs P11G3, E) P11F6 vs P11G5, F) P11G3 vs P11G5. (PDF 125 kb) Additional file 2: Figure S2. Heat map of selected genes (mentioned in the text) in the genomes of *Psychrobacter* species based on KEGG Orthology (KO) gene counts. Figure S3. Complete heat map of genes with KO number in the genomes of *Psychrobacter* species. (PDF 8085 kb) Additional file 3: Table S1. Predicted amino acid residues forming substrate binding pocket. (PDF 87 kb)

## Abbreviations

2,6-DMP: 2,6-Dimethoxyphenol
ABTS: 2,2'-azino-bis-3-ethylbenzothiazoline-6-sulphonic acid
ANI: Average Nucleotide Identity
BLAST: Basic Local Alignment Search Tool
CDS: Coding DNA Sequence
DDBJ: DNA Data Bank of Japan
EC: Enzyme Commission
EMBL: European Molecular Biology Laboratory
HTS: High Throughput Screening
HTG: Horizontally Transferred Genes
IMG: Integrated Microbial Genomes
IPTG: Isopropyl β-D-1-thiogalactopyranoside
kbp: Kilobase pair
KEGG: Kyoto Encyclopedia of Genes and Genomes
KO: KEGG Orthology
LMS: Laccase Mediator System
LMCO: Laccase-like Multicopper Oxidase
PCA: Principal Component Analysis
RAST: Rapid Annotation Using Subsystem Technology
RDP: Ribosomal Database Project
rpm: Revolutions Per Minute
WGS: Whole Genome Shotgun
