# Supplementary material for: Isolation and genome sequencing of four Arctic marine Psychrobacter strains exhibiting multicopper oxidase activity
Source: BMC Genomics. 2016 Feb 16;17:117. doi: 10.1186/s12864-016-2445-4 (PMC4754876; doi:10.1186/s12864-016-2445-4)
Supplement: Additional file 1: Figure S1. — Dot plot analysis of chromosomal DNA sequences. A) P2G3 vs P11G3, B) P11F6 vs P2G3, C) P2G3 vs P11G5, D) P11F6 vs P11G3, E) P11F6 vs P11G5, F) P11G3 vs P11G5. (PDF 125 kb) [file 12864_2016_2445_MOESM1_ESM.pdf]

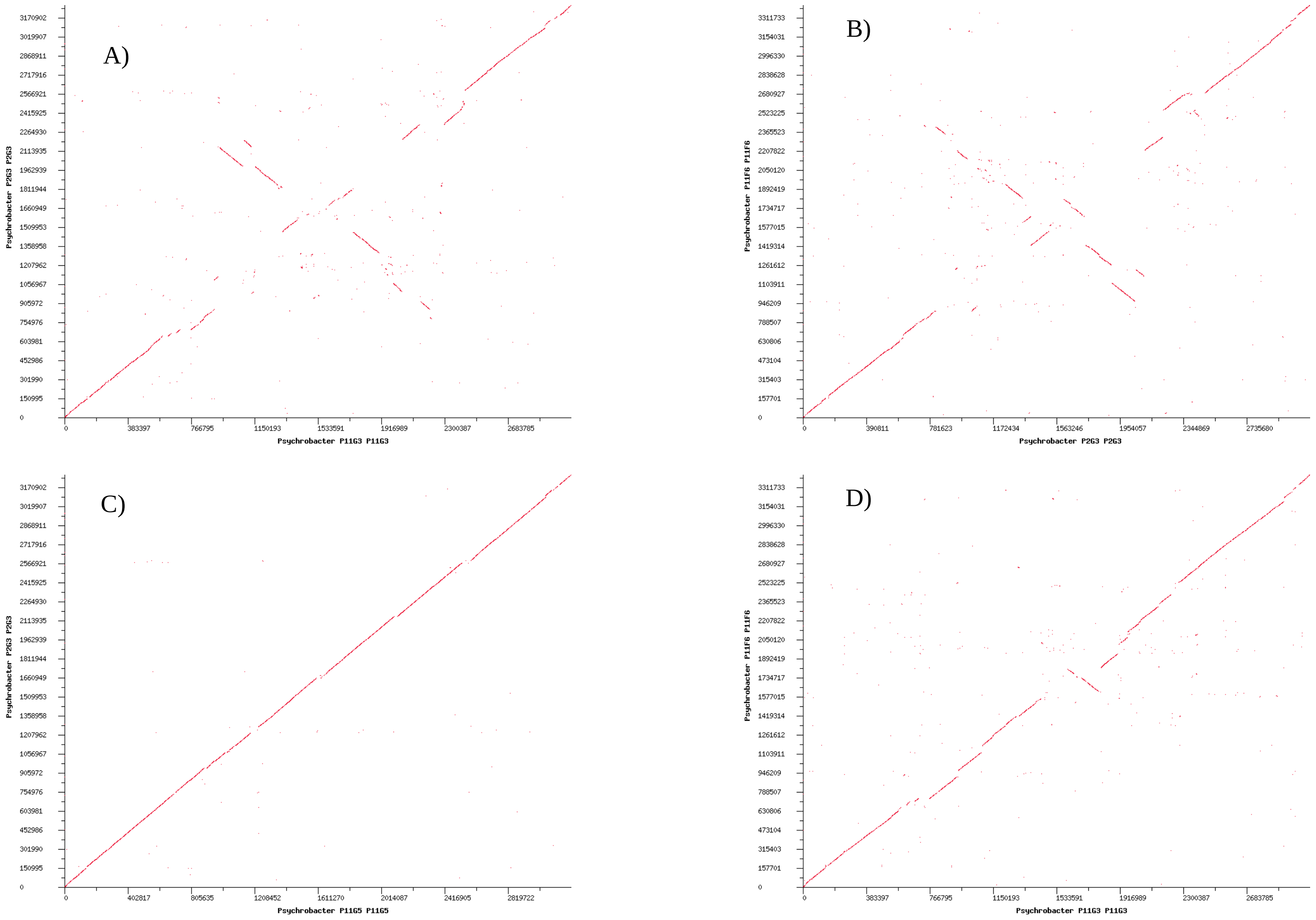

A)
Psychrobacter P2G3 P2G3
Psychrobacter P11G3 P11G3
0
150995
301990
452986
603981
754976
905972
1056967
1207962
1358958
1509953
1660949
1811944
1962939
2113935
2264930
2415925
2566921
2717916
2868911
3019907
3170902
383397
766795
1150193
1533591
1916989
2300387
2683785
B)
Psychrobacter P11F6 P11F6
Psychrobacter P2G3 P2G3
157701
315403
473104
630806
788507
946209
1103911
1261612
1419314
1577015
1734717
1892419
2050120
2207822
2365523
2523225
2680927
2838628
2996330
3154031
3311733
390811
781623
1172434
1563246
1954057
2344869
2735680
C)
Psychrobacter P2G3 P2G3
Psychrobacter P11G5 P11G5
402817
805635
1208452
1611270
2014087
2416905
2819722
D)
Psychrobacter P11F6 P11F6
Psychrobacter P11G3 P11G3

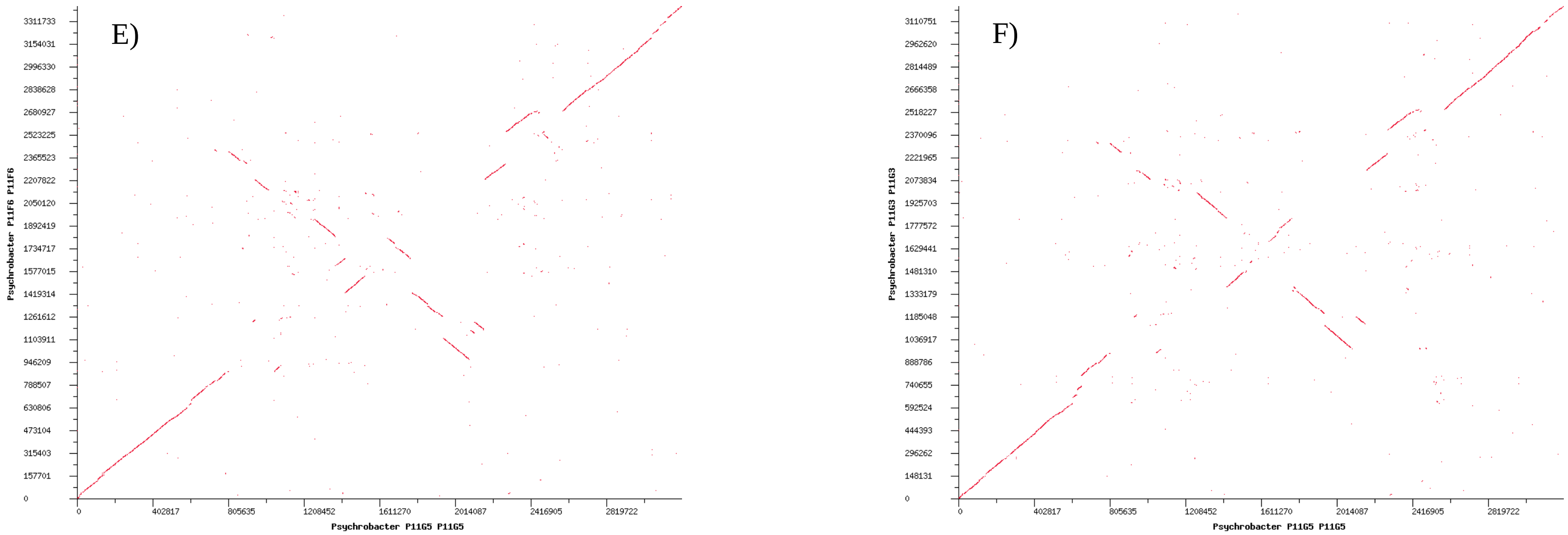

Figure S1. Dot plot analysis of chromosomal DNA sequences. A) P2G3 vs P11G3, B) P11F6 vs P2G3, C) P2G3 vs P11G5, D) P11F6 vs P11G3, E) P11F6 vs P11G5, F) P11G3 vs P11G5.
