# Supplementary material for: Isolation and genome sequencing of four Arctic marine Psychrobacter strains exhibiting multicopper oxidase activity
Source: BMC Genomics. 2016 Feb 16;17:117. doi: 10.1186/s12864-016-2445-4 (PMC4754876; doi:10.1186/s12864-016-2445-4)
Supplement: Additional file 2: Figure S2. — Heat map of selected genes (mentioned in the text) in the genomes of Psychrobacter species based on KEGG Orthology (KO) gene counts. Figure S3. Complete heat map of genes with KO number in the genomes of Psychrobacter species. (PDF 8085 kb) [file 12864_2016_2445_MOESM2_ESM.pdf]

	P.aquaticus CMS56	*P.arcticus* 273_4	*P.cryohalolentis* K5	*P.lutiphocae* DSM21542	*P.muricola* 2pS	*P.pacificensis* DSM23406	*P.phenylpyruvicus* DSM7000	*Psychrobacter* sp. 1501	*Psychrobacter* sp. AC24	*Psychrobacter* sp. G	*Psychrobacter* sp. JCM18900	*Psychrobacter* sp. JCM18901	*Psychrobacter* sp. JCM18902	*Psychrobacter* sp. JCM18903	*Psychrobacter* sp. LV10R520_6	*Psychrobacter* sp. P11F6	*Psychrobacter* sp. P11G3	*Psychrobacter* sp. P11G5	*Psychrobacter* sp. P2G3	*Psychrobacter* sp. PAMC21119	*Psychrobacter* sp. PRwf_1	*Psychrobacter* sp. TB15	*Psychrobacter* sp. TB2	*Psychrobacter* sp. TB47	*Psychrobacter* sp. TB67	*Psychrobacter* sp. UKMCC_SWTGB2
K00216	0	0	0	0	0	0	0	0	0	0	0	0	0	0	0	1	0	0	0	1	0	0	0	0	0	0
K00276	0	0	0	1	0	0	0	0	0	0	0	0	0	0	1	0	0	1	1	0	0	0	0	0	0	0
K00520	0	0	0	0	0	1	0	0	0	0	0	0	0	0	1	0	0	0	1	0	0	1	0	0	0	0
K00529	0	0	0	0	0	0	0	0	0	0	0	0	0	0	0	1	0	0	0	0	0	0	0	0	0	0
K00575	0	0	1	0	0	1	0	0	0	0	0	0	0	0	0	0	0	1	1	0	0	0	0	0	0	0
K01252	0	0	0	0	0	0	0	0	0	0	0	0	0	0	0	1	0	0	0	1	0	0	0	0	0	0
K01666	0	0	0	0	0	0	0	0	0	0	0	0	0	0	0	1	0	0	0	0	0	0	0	0	0	0
K01674	0	0	0	1	0	0	0	0	0	0	0	0	0	0	0	0	0	1	1	0	0	0	0	0	0	0
K01751	0	0	1	0	0	0	0	0	0	1	0	0	0	0	0	0	0	0	1	0	0	0	0	0	0	0
K01760	0	0	0	0	0	0	0	0	0	0	0	0	0	0	0	1	0	0	0	0	0	0	0	0	0	0
K01799	0	0	0	0	0	0	0	0	0	0	0	0	0	0	0	1	0	0	1	0	0	0	0	0	0	0
K02361	0	0	0	0	0	0	0	0	0	0	0	0	0	0	0	1	0	0	0	1	0	0	0	0	0	0
K02363	0	0	0	0	0	0	0	0	0	0	0	0	0	0	0	1	0	0	0	1	0	0	0	0	0	0
K03294	0	0	0	0	0	0	0	0	0	0	0	0	0	0	0	1	0	0	0	0	0	0	0	0	0	0
K03328	0	0	0	0	0	0	0	0	0	0	0	0	0	0	0	0	0	2	1	0	0	0	0	0	0	1
K03735	0	0	0	0	0	0	0	0	0	0	0	0	0	0	0	0	0	1	1	0	0	0	0	0	0	0
K03736	0	0	0	0	0	0	0	0	0	0	0	0	0	0	0	0	0	1	1	0	0	0	0	0	0	0
K04073	0	0	0	0	0	0	0	0	0	0	0	0	0	0	0	1	0	0	0	0	0	0	0	0	0	0
K05020	0	0	0	1	0	0	0	0	0	0	0	0	0	0	0	0	0	0	1	0	0	0	0	0	0	0
K05710	0	0	0	0	0	0	0	0	0	0	0	0	0	0	0	1	0	0	0	0	0	0	0	0	0	0
K07240	1	0	1	0	0	0	0	0	0	1	0	0	0	0	0	0	0	0	1	0	0	0	0	0	0	0
K07705	0	0	1	0	0	0	0	0	0	0	0	0	0	0	0	0	0	1	1	0	0	0	0	0	0	0
K16242	0	0	0	0	0	0	0	0	0	0	0	0	0	0	0	1	0	1	1	0	0	0	0	0	0	0
K16243	0	0	0	0	0	0	0	0	0	0	0	0	0	0	0	1	0	1	1	0	0	0	0	0	0	0
K16244	0	0	0	0	0	0	0	0	0	0	0	0	0	0	0	1	0	1	1	0	0	0	0	0	0	0
K16245	0	0	0	0	0	0	0	0	0	0	0	0	0	0	0	1	0	1	1	0	0	0	0	0	0	0
K16246	0	0	0	0	0	0	0	0	0	0	0	0	0	0	0	1	0	1	1	0	0	0	0	0	0	0
K16249	0	0	0	0	0	0	0	0	0	0	0	0	0	0	0	1	0	1	1	0	0	0	0	0	0	0

Figure S2. Heat map of selected genes (mentioned in the text) in the genomes of *Psychrobacter* species based on KEGG Orthology (KO) gene counts.

	P.aquaticus CMS56	*P.arcticus* 273_4	*P.cryohalolentis* K5	*P.lutiphocae* DSM21542	*P.muriicola* 2pS	*P.pacificensis* DSM23406	*P.phenylpyruvicus* DSM7000	*Psychrobacter* sp. 1501	*Psychrobacter* sp. AC24	*Psychrobacter* sp. G	*Psychrobacter* sp. JCM18900	*Psychrobacter* sp. JCM18901	*Psychrobacter* sp. JCM18902	*Psychrobacter* sp. JCM18903	*Psychrobacter* sp. LV10R520_6	*Psychrobacter* sp. P11F6	*Psychrobacter* sp. P11G3	*Psychrobacter* sp. P11G5	*Psychrobacter* sp. P2G3	*Psychrobacter* sp. PAMC21119	*Psychrobacter* sp. PRwf_1	*Psychrobacter* sp. TB15	*Psychrobacter* sp. TB2	*Psychrobacter* sp. TB47	*Psychrobacter* sp. TB67	*Psychrobacter* sp. UKMCC_SWTGB2
K00001	2	2	2	1	2	2	0	1	2	2	2	2	2	2	1	2	2	2	2	2	1	2	2	1	2	2
K00003	1	1	1	1	1	1	1	1	1	1	1	1	1	1	1	1	1	1	1	1	1	1	1	1	1	1
K00004	1	0	0	0	2	0	1	1	0	0	0	0	0	0	1	0	0	0	0	0	1	0	0	0	0	1
K00012	2	1	1	1	2	2	2	3	3	1	1	2	2	1	1	2	2	2	1	2	2	2	2	3	3	2
K00013	1	1	1	1	1	1	1	1	1	1	1	1	1	1	1	1	1	1	1	1	1	1	1	1	1	1
K00014	1	1	1	1	1	1	1	1	1	1	1	1	1	1	1	1	1	1	1	1	1	1	1	1	1	1
K00018	1	1	1	2	1	1	2	2	1	1	1	1	1	1	1	1	1	2	2	1	1	2	2	1	1	1
K00019	1	1	1	0	1	1	0	1	1	1	1	1	1	1	0	1	1	1	1	1	1	0	0	1	1	1
K00020	1	1	1	2	2	1	2	2	1	1	2	1	1	1	1	1	1	2	1	1	2	1	1	1	1	2
K00023	0	0	0	0	0	0	1	1	0	0	0	0	0	0	0	0	0	1	1	0	0	0	0	0	0	0
K00024	1	1	1	1	1	1	1	1	1	1	1	1	1	1	1	1	1	1	1	1	1	1	1	1	1	1
K00027	1	1	1	0	1	1	0	0	1	1	1	1	1	1	1	1	1	1	1	1	0	1	1	1	1	1
K00029	1	1	1	1	1	1	1	1	1	1	1	1	1	1	1	1	1	1	1	1	1	1	1	1	1	1
K00031	2	1	2	1	2	2	2	2	2	2	2	2	2	2	2	2	2	2	2	2	2	2	2	2	2	2
K00038	0	0	0	1	0	0	1	1	1	0	0	0	1	1	0	1	0	1	1	0	1	0	0	1	1	0
K00052	1	1	1	1	1	1	1	1	1	1	1	1	1	1	1	1	1	1	1	1	1	1	1	1	1	1
K00053	1	1	1	1	1	1	1	1	1	1	1	1	1	1	1	1	1	1	1	1	1	1	1	1	1	1
K00054	0	0	0	0	0	0	0	0	0	0	0	0	0	0	0	0	0	0	0	0	0	0	0	0	0	1
K00055	0	0	0	0	1	0	0	0	0	0	1	0	1	0	1	0	0	1	1	0	0	0	0	0	0	1
K00057	1	1	1	1	1	1	1	1	1	1	1	1	1	1	1	1	1	1	1	1	1	1	1	1	1	1
K00058	1	1	1	1	1	1	1	1	1	1	1	1	1	1	1	1	1	1	1	1	1	1	1	1	1	1
K00059	3	3	3	3	3	3	3	3	3	3	4	4	2	3	3	2	4	3	4	3	3	3	3	3	3	2
K00060	0	0	0	0	1	0	0	0	0	0	0	1	1	0	0	1	1	0	0	1	0	0	0	0	0	1
K00067	1	1	1	1	0	0	0	0	0	0	0	0	0	0	0	0	0	0	0	0	0	0	0	0	0	0
K00074	0	1	1	1	1	1	0	1	1	1	2	0	1	0	1	0	2	1	1	1	0	2	2	1	1	1
K00075	1	1	1	1	1	1	1	1	1	1	1	1	1	1	1	1	1	1	1	1	1	1	1	1	1	1
K00076	0	0	1	1	1	0	1	1	0	1	0	0	2	1	0	2	0	1	1	0	1	0	0	0	0	0
K00086	0	0	0	0	1	0	0	0	0	0	0	0	0	0	0	0	0	0	0	0	0	0	0	0	0	0
K00088	1	1	1	1	1	1	1	1	1	1	1	1	1	1	1	1	1	1	1	1	1	1	1	1	1	1
K00097	1	1	1	1	1	1	1	1	1	1	1	1	1	1	1	1	1	1	1	1	1	1	1	1	1	1
K00099	1	1	1	1	1	1	1	1	1	1	1	1	1	1	1	1	1	1	1	1	1	1	1	1	1	1
K00100	1	0	0	0	1	1	1	1	2	0	1	1	2	1	0	1	1	1	1	1	1	1	1	2	2	1
K00101	1	1	1	1	2	1	1	1	2	1	3	1	2	2	1	2	1	2	1	0	1	1	1	2	2	2
K00102	0	0	0	1	1	0	1	1	0	0	1	1	1	0	0	1	1	1	1	0	1	0	0	0	0	1
K00104	0	1	1	0	1	0	0	0	0	1	0	0	0	0	1	0	0	1	1	0	0	0	0	0	0	0
K00108	1	1	1	1	1	1	1	0	1	1	2	1	2	1	1	1	2	1	1	1	0	0	0	1	1	1
K00111	0	0	1	1	1	0	1	1	1	1	0	0	1	1	0	1	0	0	0	1	0	1	1	1	1	0
K00117	1	1	0	0	1	1	0	1	0	1	1	1	0	0	0	0	1	0	0	0	1	0	0	0	0	1
K00121	1	1	1	1	1	1	1	1	1	1	1	1	1	2	1	1	1	1	1	2	1	1	1	1	1	2
K00128	2	1	3	3	3	1	1	1	2	3	3	1	4	3	2	4	3	3	3	3	1	2	2	2	2	2
K00130	2	1	1	1	3	2	1	0	1	1	4	2	2	2	2	2	2	2	1	2	0	0	0	1	1	1
K00133	1	1	1	1	1	1	1	1	1	1	1	1	1	1	1	1	1	1	1	1	1	1	1	1	1	1
K00134	3	2	2	2	2	2	2	2	2	2	2	2	2	2	2	2	2	2	2	2	2	2	2	2	2	2
K00135	3	1	3	3	3	3	2	1	3	3	4	3	3	3	3	4	3	3	3	3	2	2	2	3	3	3
K00138	1	1	1	0	2	1	1	1	1	1	1	1	1	1	1	1	1	1	1	1	1	1	1	1	1	1
K00140	1	1	1	1	2	2	1	1	1	1	2	1	1	1	1	1	3	2	2	1	1	1	1	1	1	1
K00141	0	0	0	0	1	0	0	0	0	0	0	0	0	0	1	0	0	0	0	0	0	0	0	0	0	0
K00145	1	1	1	1	1	1	1	1	1	1	1	1	1	1	1	1	1	1	1	1	1	1	1	1	1	1
K00147	1	1	1	1	1	1	1	1	1	1	1	1	1	1	1	1	1	1	1	1	1	1	1	1	1	1
K00154	1	1	1	1	1	2	1	1	1	1	2	1	1	1	1	1	1	1	1	1	1	1	1	1	1	1
K00158	0	0	0	0	0	0	0	0	0	0	0	0	0	0	0	0	0	0	0	0	1	0	0	0	0	0
K00163	1	1	1	1	1	1	1	1	1	1	1	1	1	1	1	1	1	1	1	1	1	1	1	1	1	1
K00164	1	1	1	1	1	1	1	1	1	1	1	1	1	1	1	1	1	1	1	1	1	1	1	1	1	1
K00208	1	1	1	1	1	1	1	1	1	1	1	1	1	1	1	1	1	1	1	1	1	1	1	1	1	1

K00215	1	1	1	1	1	1	1	1	1	1	1	1	1	1	1	1	1	1	1	1	1	1	1	1	1	1
K00216	0	0	0	0	0	0	0	0	0	0	0	0	0	0	0	1	0	0	0	1	0	0	0	0	0	0
K00219	1	1	1	1	1	1	1	1	1	1	1	1	1	1	1	1	1	1	1	1	1	1	1	1	1	1
K00228	1	1	1	1	1	1	1	1	1	1	1	1	1	1	1	1	1	1	1	1	1	1	1	1	1	1
K00239	1	1	1	1	1	1	1	1	1	1	1	1	1	1	1	1	1	1	1	1	1	1	1	1	1	1
K00240	1	1	1	1	1	1	1	1	1	1	1	1	1	1	1	1	1	1	1	1	1	1	1	1	1	1
K00241	1	1	1	1	1	1	0	1	1	1	1	1	1	1	1	1	1	1	1	1	1	1	1	1	1	1
K00242	1	1	1	1	1	1	1	1	1	1	1	1	1	1	1	1	1	1	1	1	1	1	1	1	1	1
K00244	0	0	0	1	0	0	1	0	0	0	0	0	0	0	0	0	0	0	0	0	0	0	0	0	0	0
K00245	0	0	0	1	0	0	1	0	0	0	0	0	0	0	0	0	0	0	0	0	0	0	0	0	0	0
K00246	0	0	0	1	0	0	1	0	0	0	0	0	0	0	0	0	0	0	0	0	0	0	0	0	0	0
K00247	0	0	0	1	0	0	1	0	0	0	0	0	0	0	0	0	0	0	0	0	0	0	0	0	0	0
K00248	1	0	1	0	1	1	1	1	1	1	1	1	1	1	1	1	1	1	1	1	1	1	1	1	1	1
K00252	1	1	1	1	1	1	1	1	1	1	1	1	1	1	1	1	1	1	1	1	1	1	1	0	1	1
K00253	1	1	1	1	1	1	1	1	1	1	1	1	1	1	1	1	1	1	1	1	1	1	1	1	1	1
K00254	1	1	1	1	1	1	1	1	1	1	1	1	1	1	1	1	1	1	1	1	1	1	1	1	1	1
K00255	0	0	1	1	1	1	1	1	2	1	1	1	1	1	1	1	1	1	1	1	1	1	1	2	2	1
K00257	4	4	4	4	5	4	4	3	4	4	4	5	4	4	4	4	4	5	5	4	3	4	4	4	4	3
K00259	0	0	0	0	0	0	0	0	0	0	0	0	0	0	1	0	0	1	1	0	0	1	1	0	0	0
K00262	1	1	1	1	1	0	1	1	1	1	1	1	1	1	1	1	0	1	1	0	1	1	1	1	1	1
K00265	1	1	1	1	1	1	1	1	1	1	1	1	1	1	1	1	1	1	1	1	1	1	1	1	1	1
K00266	1	1	1	1	1	1	1	1	1	1	1	0	1	1	1	1	1	1	1	1	1	1	1	1	1	1
K00274	0	1	2	0	2	0	1	0	1	2	1	1	1	1	2	1	1	2	2	2	1	1	1	1	1	0
K00275	1	1	1	1	1	1	1	1	1	1	1	1	1	1	1	1	1	1	1	1	1	1	1	1	1	1
K00276	0	0	0	1	0	0	0	0	0	0	0	0	0	0	1	0	0	1	1	0	0	0	0	0	0	0
K00278	1	1	1	1	1	1	1	1	1	1	1	1	1	1	1	1	1	1	1	1	1	1	1	1	1	1
K00281	1	1	1	1	1	1	1	1	1	1	1	1	1	0	1	1	1	1	1	1	1	1	1	1	1	1
K00285	2	2	4	2	2	2	1	1	3	4	2	1	2	3	2	3	3	3	3	4	1	2	2	3	3	3
K00286	1	1	1	1	1	1	1	1	1	1	1	1	1	1	1	1	1	1	1	1	1	1	1	1	1	1
K00287	1	1	1	1	1	1	1	1	1	1	1	1	1	1	1	1	1	1	1	1	1	1	1	1	1	1
K00292	1	1	1	0	1	1	1	1	1	1	1	1	1	1	1	1	1	0	1	1	1	1	1	1	1	1
K00297	1	1	1	1	1	1	1	1	1	1	1	1	1	1	1	1	1	1	1	1	1	1	1	1	1	1
K00311	1	1	1	1	1	1	1	1	1	1	1	1	1	1	1	1	1	1	1	1	1	1	1	1	1	1
K00322	1	1	1	1	1	1	1	1	1	1	1	1	1	1	1	1	1	1	1	1	1	1	1	1	1	1
K00324	2	2	2	2	2	2	2	2	2	2	2	2	2	2	2	2	2	2	2	2	2	2	2	2	2	2
K00325	1	1	1	1	1	1	1	1	1	1	1	1	1	1	1	1	1	1	1	1	1	1	1	1	1	1
K00330	1	1	1	1	1	1	1	1	1	1	1	1	1	1	1	1	1	1	1	1	1	1	1	1	1	1
K00331	1	1	1	1	1	1	1	1	1	1	1	1	1	1	1	1	1	1	1	1	1	1	1	1	1	1
K00334	1	1	1	1	1	1	1	1	1	1	1	1	1	1	1	1	1	1	1	1	1	1	1	1	1	1
K00335	1	1	1	1	1	1	1	1	1	1	1	1	1	1	1	1	1	1	1	1	1	1	1	1	1	1
K00336	1	1	1	1	1	1	1	1	1	1	1	1	1	1	1	1	1	1	1	1	1	1	1	1	1	1
K00337	1	1	1	1	1	1	1	1	1	1	1	1	1	1	1	1	1	1	1	1	1	1	1	1	1	1
K00338	1	1	1	1	1	1	1	1	1	1	1	1	1	1	1	1	1	1	1	1	1	1	1	1	1	1
K00339	1	1	1	1	1	1	1	1	1	1	1	1	1	1	1	1	1	1	1	1	1	1	1	1	1	1
K00340	1	1	1	0	1	1	1	1	1	1	1	1	1	1	1	1	1	1	1	1	1	1	1	1	1	1
K00341	1	1	1	1	1	1	1	1	1	1	1	1	1	1	1	1	1	1	1	1	1	1	1	1	1	1
K00342	1	1	1	1	1	1	1	1	1	1	1	1	1	1	1	1	1	1	1	1	1	1	1	0	1	1
K00343	1	1	1	1	1	1	1	1	1	1	1	1	1	1	1	1	1	1	1	1	1	1	1	1	1	1
K00344	1	1	1	1	1	2	1	2	2	1	2	1	1	1	1	2	1	1	1	2	1	2	2	1	2	2
K00346	1	1	1	1	1	1	1	1	1	1	1	1	0	1	1	1	1	1	1	1	1	1	1	1	1	1
K00347	1	1	1	1	1	1	1	1	1	1	1	1	1	1	1	1	1	1	1	1	1	1	1	1	1	1
K00348	1	1	1	1	1	1	1	1	1	1	1	1	1	1	1	1	1	1	1	1	1	1	1	1	1	1
K00349	1	1	1	1	1	1	1	1	1	1	1	1	1	0	1	1	1	1	1	1	1	1	1	1	1	1
K00350	1	1	1	1	1	1	1	1	1	1	1	1	1	1	1	1	1	1	1	1	1	1	1	1	1	1
K00351	1	1	1	1	1	1	1	1	1	1	1	1	1	1	1	1	1	1	1	1	1	1	1	1	1	1
K00362	1	0	1	1	0	0	1	1	2	1	0	1	0	0	0	0	1	0	0	1	1	0	0	2	2	1
K00363	1	0	1	1	0	0	1	1	1	1	0	0	0	0	0	0	1	0	0	1	1	0	0	1	1	1
K00368	0	0	0	0	0	0	0	1	0	0	0	0	0	0	0	0	0	0	0	0	0	0	0	0	0	0
K00370	1	1	0	1	0	0	0	0	0	0	0	0	1	1	1	1	0	1	1	0	1	0	0	0	0	0
K00371	1	1	0	1	0	0	0	0	0	0	0	0	1	1	1	1	0	1	1	0	1	0	0	0	0	0
K00372	1	0	1	1	0	0	1	1	1	1	0	1	0	0	0	0	1	0	0	1	1	0	0	1	1	1
K00373	1	1	0	1	0	0	0	0	0	0	0	0	1	1	1	1	0	1	1	0	1	0	0	0	0	0
K00374	1	1	0	1	0	0	0	0	0	0	0	0	1	1	1	1	0	1	1	0	1	0	0	0	0	0
K00381	1	1	1	1	1	1	1	1	1	1	1	1	1	1	1	1	1	1	1	1	1	1	1	1	1	1
K00382	2	2	2	1	2	2	2	2	2	2	2	2	2	2	2	2	2	2	2	2	2	2	2	2	2	2
K00383	0	1	1	1	1	1	1	1	1	1	1	1	1	1	1	1	1	1	1	1	1	1	1	1	1	0
K00384	1	1	1	1	1	1	1	1	1	1	1	1	1	1	1	1	1	1	1	1	1	1	1	1	1	1
K00390	1	1	1	1	1	1	1	1	1	1	1	1	1	1	1	1	1	1	1	1	1	1	1	1	1	1

K00404	1	1	1	1	1	1	1	1	1	1	1	1	1	1	1	1	1	1	1	1	1	1	1	1	1	1
K00405	1	1	1	1	1	1	1	1	1	1	1	1	1	1	1	1	1	1	1	1	1	1	1	1	1	1
K00406	1	1	1	1	1	1	1	1	1	1	1	1	1	1	1	1	1	1	1	1	1	1	1	1	1	1
K00407	1	1	1	1	1	1	1	1	1	1	1	1	1	1	1	1	1	1	1	1	1	1	1	1	1	1
K00411	1	1	1	1	1	1	1	1	1	1	1	1	1	1	1	1	1	1	1	1	1	1	1	1	1	1
K00412	1	1	1	1	1	1	1	1	1	1	1	1	1	1	1	1	1	1	1	1	1	1	1	1	1	1
K00413	1	1	1	1	1	1	1	1	1	1	1	1	1	1	1	1	1	1	1	1	1	1	1	1	1	1
K00425	1	1	1	1	1	1	1	2	1	1	1	1	1	1	1	1	1	1	1	1	1	1	1	1	1	1
K00426	1	1	1	1	1	1	1	2	1	1	1	1	1	1	1	1	1	1	1	1	1	1	1	1	1	1
K00428	0	0	0	1	0	0	1	0	0	0	0	0	0	0	0	0	0	0	0	0	1	0	0	0	0	0
K00432	1	0	1	1	1	1	1	1	1	0	0	1	1	1	1	1	1	1	1	1	0	1	1	1	1	1
K00448	0	0	0	1	1	0	0	0	0	0	1	0	1	0	1	0	0	1	1	0	0	0	0	0	0	0
K00449	0	0	0	1	1	0	0	0	0	0	1	0	1	0	1	0	0	1	1	0	0	0	0	0	0	0
K00450	0	0	0	0	0	0	0	0	0	0	0	0	0	0	0	0	0	0	0	0	1	0	0	0	0	0
K00457	1	0	1	0	1	1	0	1	0	1	1	1	1	1	1	1	1	1	1	1	1	0	0	0	0	1
K00459	1	1	1	0	1	1	1	1	1	1	1	1	1	1	1	2	1	1	1	1	1	0	0	1	1	1
K00471	0	0	0	0	0	0	0	0	0	0	0	0	0	0	2	0	1	0	0	0	0	0	0	0	0	0
K00472	0	0	0	1	0	0	1	0	0	0	0	0	0	0	0	0	0	0	0	0	1	1	1	0	0	0
K00480	0	0	0	0	0	0	1	0	0	0	0	0	0	0	0	0	0	0	0	0	0	0	0	0	0	0
K00481	0	0	0	1	1	0	0	0	0	0	0	0	1	0	1	0	0	1	1	0	0	0	0	0	0	0
K00496	0	0	0	1	0	0	1	1	0	0	0	0	0	0	0	0	0	0	0	0	1	0	0	0	0	0
K00499	0	0	0	1	0	0	0	0	0	0	1	0	0	0	0	0	0	0	0	0	0	0	0	0	0	0
K00500	1	0	1	0	1	1	0	1	0	1	1	1	1	1	1	1	1	1	1	1	1	0	0	0	0	1
K00507	1	1	1	1	1	1	1	1	1	1	1	1	1	1	1	1	1	1	1	1	1	1	1	1	1	1
K00508	1	0	1	0	1	1	0	0	1	1	1	1	1	1	1	1	1	1	1	1	0	1	1	1	1	1
K00518	0	0	0	0	0	0	1	0	0	0	0	0	0	0	0	0	0	0	0	0	0	0	0	0	0	0
K00520	0	0	0	0	0	1	0	0	0	0	0	0	0	0	1	0	0	0	1	0	0	1	0	0	0	0
K00525	1	1	1	1	1	1	1	1	1	1	1	1	1	1	1	1	1	1	1	1	1	1	1	1	1	1
K00526	1	1	1	1	1	1	1	1	1	1	1	1	1	1	1	1	1	1	1	1	1	1	1	1	1	1
K00527	0	0	0	1	0	0	0	0	0	0	0	0	0	0	0	0	0	0	0	0	0	0	0	0	0	0
K00528	2	2	2	2	2	2	2	2	2	2	2	2	2	2	2	2	2	2	2	2	2	2	2	2	2	2
K00529	0	0	0	0	0	0	0	0	0	0	0	0	0	0	0	1	0	0	0	0	0	0	0	0	0	0
K00537	1	1	1	1	1	1	1	0	1	1	1	1	1	1	0	1	1	1	1	1	1	1	1	1	1	1
K00540	1	3	1	2	2	3	2	2	4	1	1	1	3	2	1	1	2	2	2	2	1	2	2	4	4	3
K00548	1	1	1	1	1	1	1	1	1	1	1	1	1	1	1	1	1	1	1	1	1	1	1	0	1	0
K00549	1	1	1	1	1	1	2	1	1	1	1	1	1	1	2	1	1	1	1	1	1	1	1	1	1	1
K00554	1	1	1	1	1	1	1	1	1	1	1	1	1	1	1	1	1	1	1	1	1	1	1	1	1	1
K00556	0	0	0	0	0	0	0	1	0	0	0	0	0	0	0	0	0	0	0	0	0	0	0	0	0	0
K00558	1	2	0	1	2	0	0	0	1	1	1	0	0	2	0	0	1	0	0	0	0	0	0	1	1	0
K00560	1	1	1	1	1	1	1	2	1	1	1	1	1	1	1	1	1	1	1	1	1	2	2	1	1	1
K00563	1	1	1	1	1	1	1	1	1	1	1	1	1	1	1	1	1	1	1	1	1	1	1	1	1	1
K00566	1	1	1	1	1	1	1	1	1	1	1	1	1	1	1	1	1	1	1	1	1	1	1	1	1	1
K00567	1	1	1	1	1	1	1	1	1	1	1	1	1	1	1	1	1	1	1	1	1	1	1	1	1	1
K00568	1	1	1	1	1	1	1	1	1	1	1	1	1	1	1	1	1	1	1	1	1	1	1	1	1	1
K00569	2	1	3	1	2	2	1	1	2	3	2	2	2	3	2	2	2	2	2	3	1	2	2	2	2	2
K00571	0	0	0	0	0	1	0	0	1	0	0	0	0	0	0	0	0	1	0	0	0	1	1	1	1	0
K00574	1	1	1	0	1	1	0	0	1	1	1	1	1	1	1	1	1	1	1	1	1	1	1	1	1	1
K00575	0	0	1	0	0	1	0	0	0	0	0	0	0	0	0	0	0	1	1	0	0	0	0	0	0	0
K00590	0	0	0	1	0	0	0	0	0	0	0	0	0	0	0	0	0	0	0	0	0	0	0	0	0	0
K00600	1	1	1	1	1	1	1	1	1	1	1	1	1	1	1	1	1	1	1	1	1	1	1	1	1	1
K00602	1	1	1	1	1	1	1	1	1	1	1	1	1	1	1	1	1	1	1	1	1	1	1	1	1	1
K00604	2	2	1	1	1	1	2	1	1	1	1	1	1	1	3	1	1	1	1	1	1	2	2	1	1	1
K00605	1	1	1	1	1	1	1	1	1	1	1	1	1	1	1	1	1	1	1	1	1	1	1	1	1	1
K00606	1	1	1	1	1	1	1	1	1	1	1	1	1	1	1	1	1	1	1	1	1	1	1	1	1	1
K00609	1	1	1	1	1	1	1	1	1	1	1	1	1	1	1	1	1	1	1	1	1	1	1	1	1	1
K00611	0	0	0	0	0	0	0	0	0	0	0	0	0	0	1	0	0	0	0	1	0	0	0	0	0	0
K00615	1	1	1	1	1	1	1	1	1	1	1	1	1	1	1	1	1	1	1	1	1	1	1	1	1	1
K00616	1	1	1	1	1	1	1	1	1	1	1	1	1	1	1	1	1	1	1	1	1	1	1	1	1	1
K00620	1	1	1	1	1	1	1	1	1	1	1	1	1	1	1	1	1	1	1	1	1	1	1	0	1	1
K00624	0	0	0	1	0	0	1	0	0	0	0	0	0	0	0	0	0	0	0	0	0	0	0	0	0	0
K00626	4	4	5	3	5	4	3	3	4	5	4	2	4	2	4	4	4	5	5	4	3	5	5	4	4	5
K00627	1	1	1	1	1	1	1	1	1	1	1	1	1	1	1	1	1	1	1	1	1	1	1	1	1	1
K00631	1	1	1	0	1	1	1	1	1	1	1	1	1	1	1	1	1	1	1	1	1	1	1	1	1	1
K00632	1	1	1	2	1	1	1	1	1	1	1	1	1	1	2	1	1	1	1	1	1	1	1	1	1	1
K00635	1	1	1	2	1	1	2	2	1	1	1	1	1	1	1	1	1	1	1	1	2	1	1	1	1	1
K00639	0	0	0	0	1	0	0	0	0	0	0	1	1	0	0	1	1	0	0	1	0	0	0	0	0	1
K00640	1	1	1	1	2	1	1	0	1	1	1	1	1	1	1	1	1	1	1	1	1	1	1	1	1	1
K00641	1	1	1	1	1	1	1	1	1	1	1	1	1	1	2	1	1	1	1	1	1	1	1	1	1	1

K00645	2	2	1	1	2	1	1	1	1	2	1	1	1	1	2	1	1	1	2	1	1	1	1	1	1	2
K00647	1	1	1	0	1	1	0	0	1	1	1	1	1	1	1	1	1	1	1	1	0	1	1	1	1	1
K00652	1	1	1	1	1	1	1	1	1	1	1	1	1	1	1	1	1	1	1	1	1	0	0	1	1	1
K00655	1	1	1	2	1	1	2	2	1	1	1	1	1	1	1	1	1	1	1	1	2	1	1	1	1	1
K00658	1	1	1	1	1	1	1	1	1	1	1	1	1	1	1	1	1	1	1	1	1	1	1	1	1	1
K00666	2	4	4	4	5	5	5	5	7	4	3	2	3	3	4	4	5	6	6	5	4	4	4	6	7	3
K00674	1	1	1	1	1	1	1	1	1	1	1	1	1	1	1	1	1	1	1	1	1	1	1	0	1	1
K00675	0	0	0	0	0	0	0	0	1	0	0	0	0	0	0	0	0	0	0	0	1	0	0	1	1	0
K00677	1	1	2	1	1	1	1	1	1	1	1	1	1	1	1	1	1	1	1	1	1	1	1	1	1	1
K00680	0	0	1	0	0	0	0	0	0	1	1	0	1	0	0	0	0	1	0	0	0	1	1	0	0	1
K00681	1	1	1	1	1	1	0	1	1	1	1	1	1	1	1	1	1	1	1	1	1	1	1	1	1	1
K00684	1	1	1	1	1	1	1	1	1	1	1	1	1	1	1	1	1	1	1	1	1	1	1	1	1	1
K00685	0	0	0	1	0	0	1	1	0	0	0	0	0	0	0	0	0	0	0	0	1	0	0	0	0	0
K00697	1	0	1	0	0	1	1	1	1	1	0	0	0	0	0	0	0	1	1	0	0	1	1	1	1	1
K00748	1	1	1	1	1	1	1	1	1	1	1	1	1	1	1	1	1	1	1	1	1	1	1	1	1	1
K00759	1	1	1	1	1	1	1	1	1	1	1	1	1	1	1	1	1	1	1	1	1	1	1	1	1	1
K00760	1	1	1	1	1	1	1	1	1	1	1	1	1	1	1	1	1	1	1	1	1	1	1	1	1	1
K00761	1	1	1	1	1	1	1	1	1	1	1	1	1	1	1	1	1	1	1	1	1	1	1	1	1	1
K00762	1	1	1	1	1	1	1	1	1	1	1	1	1	1	1	1	1	1	1	1	1	1	1	1	1	1
K00763	1	1	1	1	1	1	1	1	1	1	1	1	1	1	1	1	1	1	1	1	1	1	1	1	1	1
K00764	1	1	1	1	1	1	1	1	1	1	1	1	1	1	1	1	1	1	1	1	1	1	1	1	1	1
K00765	1	1	1	1	1	1	1	1	1	1	1	1	1	1	1	1	1	1	1	1	1	1	1	1	1	1
K00766	1	1	1	1	1	1	1	1	1	1	1	1	1	1	1	1	1	1	1	1	1	1	1	1	1	1
K00767	1	1	1	1	1	1	1	1	1	1	1	1	1	1	1	1	1	1	1	1	1	1	1	1	1	1
K00768	1	0	1	1	1	0	1	1	1	1	0	0	1	1	0	1	0	1	1	0	1	0	0	1	1	1
K00772	1	1	1	0	1	0	0	0	1	1	1	1	1	1	1	1	1	1	1	1	0	1	1	1	1	0
K00773	1	1	1	1	1	1	1	1	1	1	1	1	1	0	1	1	1	1	1	1	1	1	1	1	1	1
K00783	1	1	1	1	1	1	1	1	1	1	1	1	1	1	1	1	1	1	1	1	1	1	1	1	1	1
K00784	1	0	1	1	2	1	1	1	2	1	1	1	1	1	1	1	1	1	1	1	1	1	1	2	2	1
K00785	0	0	0	0	0	0	1	0	0	0	0	0	0	0	0	0	0	0	0	0	0	1	1	0	0	0
K00786	0	0	0	0	0	0	1	0	0	0	0	0	0	0	0	0	0	0	0	0	0	0	0	0	0	0
K00788	1	1	1	1	1	1	1	1	1	1	1	1	1	1	1	1	1	1	1	1	1	1	1	1	1	1
K00789	1	1	1	1	1	1	1	1	1	1	1	1	1	1	1	1	1	1	1	3	1	1	1	1	1	1
K00790	1	1	1	1	1	1	1	1	1	1	1	1	1	1	1	1	1	1	1	1	1	1	1	1	1	1
K00791	1	1	1	1	1	1	1	1	1	1	1	1	0	1	1	1	1	1	1	1	1	1	1	1	1	1
K00793	1	1	1	1	1	1	1	1	1	1	1	1	1	1	1	1	1	1	1	1	1	1	1	1	1	1
K00794	1	1	1	1	1	1	1	1	1	1	1	1	1	1	1	1	1	1	1	1	1	1	1	1	1	1
K00795	1	1	1	1	1	1	1	1	1	1	1	1	0	1	1	1	1	1	1	1	1	1	1	1	1	1
K00796	1	1	1	1	1	1	1	1	1	1	1	1	1	1	1	1	1	1	1	1	1	1	1	1	1	1
K00797	0	0	0	1	0	0	1	1	0	0	0	0	0	0	0	0	0	0	0	0	0	0	0	0	0	0
K00798	1	1	1	1	1	1	1	1	1	1	1	1	1	1	1	1	1	1	1	1	1	1	1	0	1	1
K00799	5	4	4	2	4	3	3	2	4	4	7	6	4	4	5	4	6	7	7	6	5	4	4	4	4	5
K00800	1	1	1	1	1	1	1	1	1	1	1	1	1	1	1	1	1	1	1	1	1	1	1	1	1	1
K00803	0	0	1	1	1	0	1	1	1	1	0	0	1	1	0	1	0	0	0	1	0	1	1	1	1	0
K00806	1	1	1	1	1	1	1	1	1	1	1	1	1	0	1	1	1	1	1	1	1	1	1	1	1	1
K00812	1	0	0	0	1	1	0	0	1	0	1	0	1	1	1	1	1	1	1	1	0	1	1	1	1	1
K00813	0	0	0	0	0	0	0	0	0	0	1	1	0	0	0	0	1	0	0	0	0	0	0	0	0	1
K00817	2	2	3	2	3	2	2	2	4	3	3	3	3	4	2	4	3	4	4	3	2	2	2	4	4	2
K00818	1	1	1	1	1	1	1	1	1	1	1	1	1	1	1	1	1	1	1	1	1	1	1	1	1	1
K00820	1	1	1	1	1	1	1	1	1	1	1	1	1	1	1	1	1	1	1	1	1	1	1	1	1	1
K00826	1	1	1	1	1	1	1	1	1	1	1	1	1	1	1	1	1	1	1	1	1	1	1	1	1	1
K00830	0	1	1	0	0	0	0	0	0	1	0	0	0	0	0	0	0	0	0	0	0	0	0	0	0	0
K00831	1	1	1	1	1	1	1	1	1	1	1	1	1	1	1	1	1	1	1	1	1	1	1	1	1	1
K00832	1	1	1	2	1	1	1	1	1	1	1	1	1	1	1	1	1	1	1	1	1	1	1	1	1	1
K00833	1	1	1	1	1	1	1	1	1	1	1	1	1	1	2	1	1	1	1	1	1	1	1	1	1	1
K00835	1	1	1	1	1	1	1	1	1	1	1	1	1	1	1	1	1	1	1	1	1	1	1	1	1	1
K00836	1	0	0	0	1	0	1	0	0	0	1	0	0	0	0	0	0	0	0	1	0	0	0	0	0	1
K00837	0	0	0	0	0	0	0	1	1	0	0	0	1	0	1	0	0	0	0	0	0	0	0	1	1	0
K00852	1	1	1	1	1	1	1	1	1	1	1	1	1	1	1	1	1	1	1	1	1	1	1	1	1	1
K00858	1	1	1	1	1	1	1	1	1	1	1	0	1	1	1	1	1	1	1	1	1	1	1	1	1	1
K00859	1	1	1	1	1	1	1	1	1	1	1	1	1	1	1	1	1	1	1	1	1	1	1	1	1	1
K00864	1	1	1	1	1	1	1	1	1	1	1	1	1	1	1	1	1	1	1	1	1	1	1	1	1	1
K00865	1	1	1	1	1	1	1	1	1	1	1	1	1	1	1	1	1	1	1	0	1	1	1	1	1	1
K00878	0	0	0	0	0	0	1	1	0	0	0	0	0	0	0	0	0	0	0	0	1	0	0	0	0	0
K00891	1	1	1	1	1	1	1	1	1	1	1	1	1	1	1	1	1	1	1	1	1	1	1	1	1	1
K00901	0	1	1	0	1	2	1	1	2	1	1	1	1	1	1	1	1	1	1	1	1	1	1	2	2	1
K00903	0	0	1	0	0	0	0	0	0	0	0	0	0	0	0	0	0	0	0	0	0	0	0	0	0	0
K00912	1	1	1	1	1	1	1	1	1	1	1	1	1	1	1	1	1	1	1	1	1	1	1	1	1	1

K00919	1	1	1	1	1	1	1	1	1	1	1	1	1	1	0	1	1	1	1	1	1	1	1	1	1	1
K00925	1	1	1	1	1	1	1	1	1	1	1	1	1	1	1	1	1	1	1	1	1	1	1	1	1	1
K00926	0	0	0	0	0	0	0	0	0	0	0	0	0	0	1	0	0	0	0	1	0	0	0	0	0	0
K00927	1	1	1	1	1	1	1	1	1	1	1	1	1	1	1	1	1	1	1	1	1	1	1	1	1	1
K00928	1	1	1	1	1	1	1	1	1	1	1	1	1	1	1	1	1	1	1	1	1	1	1	1	1	1
K00930	1	1	1	1	1	1	1	1	1	1	1	1	1	1	1	1	1	1	1	1	1	1	1	1	1	1
K00931	1	1	1	1	1	1	1	1	1	1	1	1	1	1	1	1	1	1	1	1	1	1	1	1	1	1
K00937	1	1	1	1	1	1	1	1	1	1	1	0	1	1	1	1	1	1	1	1	1	1	1	1	1	1
K00939	1	1	1	1	1	1	1	1	1	1	1	1	1	1	1	1	1	1	1	1	1	1	1	1	1	1
K00940	1	1	1	1	1	1	1	1	1	1	1	1	1	1	1	1	1	1	1	1	1	1	1	1	1	1
K00941	1	1	1	1	1	1	1	1	1	1	1	1	1	1	1	1	1	1	1	1	1	1	1	1	1	1
K00942	1	1	1	1	1	1	1	1	1	1	1	1	1	1	1	1	1	1	1	1	1	1	1	1	1	1
K00943	1	1	1	1	1	1	1	1	1	1	1	1	1	1	1	1	1	1	1	1	1	1	1	1	1	1
K00945	1	1	1	1	1	1	1	1	1	1	1	1	1	1	1	1	1	1	1	1	1	1	1	1	1	1
K00946	1	1	1	1	1	1	1	1	1	1	1	1	1	1	1	1	1	1	1	1	1	1	1	1	1	1
K00948	1	1	1	1	1	1	1	1	1	1	1	1	1	1	1	1	1	1	1	1	1	1	1	1	1	1
K00950	2	2	2	2	2	2	2	2	2	2	2	2	2	2	2	2	2	2	2	2	2	2	2	2	2	2
K00951	2	1	2	1	2	2	1	1	2	2	2	2	2	2	2	2	2	2	2	2	1	2	2	2	2	2
K00954	1	1	1	1	1	1	1	1	1	1	1	1	1	1	2	1	1	1	1	1	1	1	1	1	1	1
K00958	1	1	1	1	1	1	1	1	1	1	1	1	1	1	1	1	1	1	1	1	1	1	1	1	1	1
K00962	1	1	1	1	1	1	1	1	1	1	1	1	1	1	1	1	1	1	1	1	1	1	1	1	1	1
K00963	1	1	1	1	1	1	1	1	1	1	1	1	1	1	1	1	1	1	1	1	1	1	1	1	1	1
K00969	1	1	1	1	1	1	1	1	1	1	1	1	1	1	1	1	1	1	1	1	1	1	1	1	1	1
K00970	1	1	1	1	1	1	1	1	1	1	1	0	0	1	1	1	1	1	1	1	1	1	1	1	1	1
K00973	0	1	1	1	0	0	0	0	0	1	0	0	0	0	1	0	0	0	0	0	0	0	0	0	0	0
K00974	1	1	1	1	1	1	1	1	1	1	1	1	1	1	1	1	1	1	1	1	1	1	1	1	1	1
K00979	1	1	1	1	1	1	1	1	1	1	1	1	1	1	1	1	1	1	1	1	1	1	1	1	1	1
K00980	0	0	0	0	0	0	0	0	0	0	0	1	0	0	0	0	0	0	0	0	0	0	0	0	0	0
K00981	1	1	1	1	1	1	1	1	2	1	2	2	1	2	1	2	2	1	1	2	1	1	1	2	2	1
K00982	1	1	1	1	1	1	1	1	1	1	1	1	1	1	1	1	1	1	1	1	1	1	1	0	1	1
K00983	1	1	0	0	0	0	1	0	0	0	0	0	0	0	0	0	0	0	1	0	0	1	1	0	0	0
K00989	1	1	1	1	1	1	1	1	1	1	1	1	1	1	1	1	1	1	1	1	1	1	1	1	1	1
K00990	1	1	1	1	1	1	1	1	1	1	1	1	1	0	1	1	1	1	1	1	1	1	1	1	1	1
K00991	1	1	1	1	1	1	1	1	1	1	1	1	1	1	1	1	1	1	1	1	1	1	1	1	1	1
K00995	1	1	1	1	1	1	1	1	1	1	1	2	1	1	1	1	1	1	1	1	1	1	1	1	1	0
K01000	1	1	1	1	1	1	1	1	1	1	1	1	1	1	1	1	1	1	1	1	1	1	1	1	1	1
K01007	1	1	1	1	1	1	1	1	1	1	1	1	1	1	1	1	1	1	1	1	1	1	1	1	1	1
K01011	0	0	0	0	0	0	0	0	0	0	0	0	0	0	0	0	0	0	0	0	0	0	0	0	0	1
K01012	1	1	1	1	1	1	1	1	1	1	1	1	1	1	1	1	1	1	1	1	1	1	1	1	1	1
K01028	1	1	1	1	1	1	1	1	1	1	1	1	1	1	1	1	1	1	1	1	1	1	1	1	1	1
K01029	1	1	1	1	1	1	1	1	1	1	1	1	1	1	1	1	1	1	1	1	1	1	1	1	1	1
K01031	1	0	1	1	2	0	0	0	0	1	1	0	1	0	1	1	0	1	1	0	0	1	1	0	0	1
K01032	1	0	1	1	2	0	0	0	0	1	1	0	1	0	1	1	0	1	1	0	0	1	1	0	0	1
K01046	1	0	1	3	1	1	3	1	2	1	1	1	1	1	1	1	2	1	1	1	2	1	1	1	2	1
K01055	1	0	1	0	1	0	0	0	0	1	0	0	0	0	0	1	0	1	1	0	0	1	1	0	0	1
K01056	1	1	1	1	1	1	1	1	1	1	1	1	1	1	1	1	1	1	1	1	1	1	1	1	1	1
K01058	1	1	1	1	1	1	1	1	1	1	1	1	1	1	1	1	1	1	1	1	1	1	1	1	1	1
K01066	0	0	0	0	0	0	0	0	0	0	0	0	0	0	1	0	0	0	0	0	1	0	0	0	0	0
K01069	1	1	2	1	1	2	1	1	1	2	2	2	1	1	2	1	2	2	2	1	1	2	2	1	1	1
K01070	1	1	1	1	1	1	1	2	1	1	1	1	1	2	1	1	1	1	1	2	1	1	1	1	1	1
K01077	0	0	0	0	0	0	2	2	1	0	0	0	0	0	0	0	0	0	0	0	0	0	0	1	1	0
K01079	1	1	1	1	1	1	1	1	1	1	1	1	1	1	1	1	1	1	1	1	1	1	1	1	1	1
K01081	1	1	1	0	2	1	1	1	1	1	1	1	1	2	1	1	1	1	1	1	1	1	1	1	1	1
K01087	1	0	1	0	0	1	1	1	1	1	0	0	0	0	0	0	0	1	1	0	0	1	1	1	1	1
K01090	0	1	1	0	0	0	0	0	1	0	0	2	0	0	1	0	0	0	1	0	0	1	1	1	1	0
K01091	3	3	3	3	3	3	3	3	3	3	3	3	3	3	3	3	3	3	3	3	3	3	3	3	3	3
K01092	1	1	1	1	1	1	1	1	1	1	1	1	1	1	1	1	1	1	1	1	1	1	1	1	1	1
K01095	1	1	1	1	1	1	1	1	1	1	1	1	1	1	1	1	1	1	1	1	1	1	1	1	1	1
K01104	2	2	2	2	2	2	2	2	1	2	1	1	2	2	2	2	1	2	2	1	2	2	2	1	1	2
K01113	0	0	0	0	0	0	1	1	1	0	0	0	0	1	0	0	0	0	0	2	0	1	1	1	1	0
K01126	2	1	2	2	2	2	2	2	2	2	1	1	1	1	1	1	2	1	1	2	1	0	0	2	2	1
K01129	1	1	1	1	1	1	1	1	1	1	1	1	1	1	2	1	1	1	1	3	1	1	1	1	1	1
K01130	0	0	0	1	0	0	0	0	0	0	0	0	0	0	0	0	0	0	0	0	0	0	0	0	0	0
K01139	0	1	0	1	0	0	1	1	0	0	0	0	0	0	0	0	0	0	0	0	1	0	0	0	0	0
K01142	1	1	1	1	1	1	1	1	1	1	1	1	1	1	1	1	1	1	1	1	1	1	1	1	1	1
K01153	2	2	4	3	0	2	2	2	4	2	1	2	1	3	2	2	2	1	1	2	2	2	1	4	4	2
K01154	2	2	3	3	0	2	1	2	4	1	1	0	0	1	2	2	2	1	2	2	1	2	1	4	4	2
K01155	1	1	2	1	1	1	2	0	1	1	2	1	1	1	2	1	0	0	1	1	1	0	0	1	1	1

K01156	1	0	0	1	0	0	0	1	0	1	0	0	0	0	0	0	0	1	0	0	0	1	1	0	0	0
K01159	1	1	1	1	1	1	1	1	1	1	1	1	1	1	1	1	1	1	1	1	1	1	1	1	1	1
K01166	1	1	1	0	0	1	0	0	1	1	1	1	1	1	1	1	1	1	1	1	0	1	1	1	1	1
K01173	1	1	1	1	1	1	1	1	1	1	1	1	1	1	1	1	1	1	1	1	1	1	1	1	1	1
K01185	0	1	0	0	0	0	0	0	0	0	0	0	0	0	0	0	0	0	0	1	0	0	0	0	0	0
K01207	1	1	1	1	1	1	1	1	1	1	1	1	1	1	1	1	1	1	1	1	1	1	1	0	1	1
K01247	1	0	1	1	1	1	1	1	1	1	1	1	1	1	1	1	1	1	1	1	1	1	1	1	1	1
K01251	1	1	1	1	1	1	1	1	1	1	1	1	1	1	1	1	1	1	1	1	1	1	1	0	1	1
K01252	0	0	0	0	0	0	0	0	0	0	0	0	0	0	0	1	0	0	0	1	0	0	0	0	0	0
K01253	1	0	0	0	0	0	0	0	0	0	0	0	0	0	0	0	0	0	0	0	0	0	0	0	0	0
K01255	1	1	1	1	1	1	1	1	1	1	1	1	1	1	1	1	1	1	1	1	1	1	1	1	1	1
K01256	1	1	1	1	1	1	1	1	1	1	1	1	1	1	1	1	1	1	1	1	1	1	1	1	1	1
K01262	1	1	1	1	1	1	1	1	1	1	1	1	1	1	1	1	1	1	1	1	1	1	1	1	1	1
K01265	3	1	1	1	2	1	1	1	1	1	2	1	2	2	1	2	2	1	1	1	1	1	1	1	1	2
K01270	0	0	0	1	0	0	1	1	0	0	0	0	0	0	0	0	0	0	0	0	1	0	0	0	0	0
K01273	1	0	0	0	1	1	0	0	0	0	1	1	0	0	0	0	1	0	0	0	0	0	0	0	0	1
K01286	1	0	0	0	0	0	0	0	0	0	0	0	0	0	1	0	0	0	0	1	0	0	0	0	0	0
K01297	1	1	1	1	1	1	1	1	1	1	1	1	1	1	1	1	1	1	1	1	1	1	1	1	1	1
K01338	1	1	1	0	1	1	1	1	1	1	1	1	1	1	1	1	1	1	1	1	1	1	1	1	1	1
K01358	1	1	1	1	1	1	1	1	1	1	1	1	1	1	1	1	1	1	1	1	1	1	1	1	1	1
K01409	1	1	1	1	1	1	1	1	1	1	1	1	1	1	1	1	1	1	1	1	1	1	1	1	1	1
K01414	1	1	1	1	1	1	1	1	1	1	1	1	1	0	1	1	1	1	1	0	1	1	1	1	1	1
K01424	1	1	1	1	1	1	1	1	1	1	1	1	1	1	1	1	1	1	1	1	1	1	1	0	1	1
K01425	1	0	1	0	1	1	0	0	0	1	1	1	0	0	0	0	1	0	0	0	0	1	1	0	0	1
K01426	1	0	1	1	1	1	1	1	1	1	1	1	1	1	1	1	1	1	1	1	1	1	1	1	1	1
K01428	1	0	2	1	1	1	1	2	1	2	1	1	1	1	0	1	1	1	1	1	0	1	1	1	1	0
K01429	0	0	1	1	0	0	1	1	0	1	0	0	0	0	0	0	0	1	1	0	0	0	1	0	0	0
K01430	1	0	2	1	1	1	1	2	1	2	1	1	1	1	0	1	1	1	1	1	0	1	1	1	1	0
K01433	1	0	1	1	1	1	1	1	1	1	1	1	1	1	1	1	1	1	1	1	1	1	1	1	1	1
K01436	1	0	1	0	1	0	0	0	0	1	0	0	0	0	0	0	0	1	1	0	0	1	1	0	0	1
K01438	1	1	1	1	1	1	1	1	1	1	1	1	1	1	1	1	1	1	1	1	1	1	1	1	1	2
K01439	1	1	1	1	1	1	1	1	1	1	1	1	1	1	1	1	1	1	1	1	1	1	1	1	1	1
K01447	0	0	1	1	1	1	0	0	0	0	1	0	0	0	0	0	0	0	0	0	0	1	1	0	0	0
K01448	0	1	0	0	0	0	0	0	0	0	0	0	0	0	0	0	0	0	0	0	0	0	0	0	0	0
K01451	0	0	0	3	1	0	0	0	0	0	1	0	0	0	1	1	0	1	1	0	0	0	0	0	0	1
K01453	0	0	1	0	1	0	0	0	0	1	0	0	1	1	0	1	0	0	0	0	0	0	0	0	0	1
K01457	1	0	0	0	0	0	0	0	0	0	0	0	0	0	0	0	0	0	0	0	0	0	0	0	0	0
K01462	1	1	1	1	1	1	1	1	1	1	1	1	1	1	1	1	1	1	1	1	1	1	1	1	1	1
K01465	2	2	2	2	2	2	2	2	2	2	2	2	2	2	3	2	2	2	2	2	2	2	2	2	2	2
K01467	0	0	1	0	1	0	0	0	1	1	1	0	0	2	0	0	1	1	1	1	0	0	0	1	1	1
K01468	1	0	1	0	1	1	0	1	1	1	1	1	1	1	1	1	1	1	1	1	1	1	1	1	1	1
K01473	0	1	0	0	0	0	0	0	0	0	0	0	1	0	0	1	0	1	1	0	0	0	0	0	0	0
K01474	0	1	1	0	0	0	0	0	0	1	0	0	0	0	0	1	0	1	1	0	0	0	0	0	0	0
K01476	1	0	1	0	0	0	0	0	0	1	1	1	0	0	0	0	1	1	1	1	0	0	0	0	0	1
K01477	0	0	0	1	0	1	1	0	0	0	1	1	0	0	1	0	1	0	0	0	0	0	0	0	0	0
K01478	0	0	0	0	0	0	0	0	0	0	0	0	0	0	1	0	0	0	0	1	0	0	0	0	0	0
K01479	1	0	1	0	1	1	0	1	1	1	1	1	1	1	1	1	1	1	1	1	1	1	1	1	1	1
K01483	0	0	1	1	1	1	1	0	0	1	1	1	1	1	1	1	1	1	1	0	0	1	1	0	0	0
K01485	0	0	0	1	0	0	1	0	0	0	0	0	1	1	0	1	0	1	1	0	0	0	0	0	0	0
K01487	0	0	1	0	0	1	0	0	0	1	1	1	0	0	1	0	1	1	1	0	0	1	1	0	0	0
K01488	1	0	1	0	1	1	0	0	1	1	1	1	1	1	1	1	1	1	1	1	0	1	1	1	1	1
K01491	1	1	1	1	1	1	1	1	1	1	1	1	1	1	1	1	1	1	1	1	1	1	1	1	1	1
K01494	1	1	1	1	1	1	1	1	1	1	1	1	1	1	1	1	1	1	1	1	1	1	1	1	1	1
K01495	1	1	1	1	1	1	1	1	1	1	1	1	1	1	1	1	1	1	1	1	1	1	1	1	1	1
K01496	0	0	0	0	0	0	0	0	0	0	0	0	0	0	1	0	0	0	0	0	0	0	0	0	0	0
K01497	1	1	1	1	1	1	1	1	1	1	1	1	1	1	1	1	1	1	1	1	1	1	1	1	1	1
K01501	1	1	1	1	1	1	1	1	1	1	1	1	1	1	1	1	1	1	1	1	1	1	1	1	1	1
K01507	1	1	1	1	1	1	1	1	1	1	1	1	1	1	1	1	1	1	1	1	1	1	1	1	1	1
K01520	1	1	1	1	1	1	1	1	1	1	1	1	1	1	1	1	1	1	1	1	1	1	1	1	1	1
K01524	1	1	1	1	1	1	1	1	1	1	1	1	1	1	1	1	1	1	1	1	1	1	1	1	1	1
K01525	1	1	1	1	1	1	1	1	1	1	1	1	1	1	1	1	1	1	1	1	1	1	1	1	1	1
K01533	1	1	1	1	1	1	1	1	1	1	0	0	1	1	1	1	1	1	1	1	1	1	1	1	1	1
K01534	0	0	1	0	1	1	0	1	1	1	2	0	2	2	0	2	3	2	2	0	0	1	0	1	1	1
K01546	0	0	0	0	0	0	0	0	0	1	0	0	0	0	0	0	0	0	0	1	0	0	0	0	0	1
K01547	0	0	0	0	0	0	0	0	0	1	0	0	0	0	0	0	0	0	0	1	0	0	0	0	0	1
K01548	0	0	0	0	0	0	0	0	0	1	0	0	0	0	0	0	0	0	0	1	0	0	0	0	0	1
K01551	1	1	1	1	1	1	1	1	1	1	1	1	1	1	1	1	1	1	1	1	1	1	1	1	1	1
K01555	1	0	1	0	1	1	0	1	0	1	1	1	1	1	1	1	1	1	1	1	1	0	0	0	0	1

K01556	0	0	0	1	0	0	0	0	0	0	0	0	0	0	0	0	0	0	0	0	0	0	0	0	0	0
K01560	1	1	1	0	1	1	0	0	1	1	1	1	1	1	1	1	1	1	1	1	0	0	0	1	1	1
K01563	0	0	1	0	0	0	0	0	1	1	1	1	0	0	0	0	1	1	1	1	0	1	1	1	1	0
K01576	0	0	0	0	0	0	0	0	0	0	1	0	1	0	1	0	0	0	0	0	2	0	0	0	0	0
K01579	1	1	1	1	1	1	1	1	1	1	1	1	1	1	1	1	1	1	1	1	1	1	1	1	1	1
K01580	0	0	0	0	1	0	0	0	0	0	0	0	0	0	0	0	0	0	0	0	0	0	0	0	0	0
K01581	1	1	1	1	1	1	1	1	1	1	0	1	1	1	1	2	1	1	1	1	1	1	1	1	1	1
K01586	1	1	2	1	1	1	1	1	1	2	1	1	1	1	1	1	1	1	1	1	1	1	1	1	1	1
K01588	1	1	1	1	1	1	1	1	1	1	1	1	1	1	1	1	1	1	1	1	1	1	1	1	1	1
K01589	1	1	1	1	1	1	1	1	1	1	1	1	1	1	1	1	1	1	1	1	1	1	1	1	1	1
K01590	0	0	0	0	0	0	0	0	0	0	0	0	0	0	0	0	0	0	0	1	0	0	0	0	0	0
K01591	1	1	1	1	1	1	1	1	1	1	1	1	1	1	1	1	1	1	1	1	1	1	1	1	1	1
K01595	1	1	1	1	1	1	1	1	2	1	1	1	1	1	1	1	1	1	1	1	1	1	1	2	2	1
K01599	1	1	1	1	1	1	1	1	1	1	1	1	1	1	1	1	1	1	1	1	1	1	1	1	1	1
K01607	1	1	1	1	1	1	2	1	1	1	2	1	0	1	0	2	1	1	1	0	1	1	1	1	1	2
K01609	1	1	1	1	1	1	1	1	1	1	1	1	1	1	1	1	1	1	1	1	1	1	1	1	1	1
K01611	0	0	0	1	0	0	1	0	0	0	0	0	0	0	0	0	0	0	0	0	0	0	0	0	0	0
K01613	1	1	1	1	1	1	1	1	1	1	1	1	1	1	1	1	1	1	1	1	1	1	1	1	1	1
K01620	0	0	0	1	0	0	1	1	0	0	0	0	0	0	0	0	0	0	0	0	1	0	0	0	0	0
K01623	1	1	1	1	1	1	1	1	1	1	0	0	1	1	1	1	1	1	1	1	1	1	1	1	1	1
K01624	1	1	1	0	1	1	0	0	1	1	1	1	1	1	1	1	1	1	1	1	0	1	1	1	1	1
K01626	1	1	1	1	1	1	1	1	1	1	1	1	1	1	1	2	1	1	1	1	1	1	1	1	1	1
K01627	1	1	1	1	1	1	1	1	1	1	1	1	1	1	1	1	1	1	1	1	1	1	1	1	1	1
K01633	1	1	1	1	1	1	1	1	1	1	1	1	1	1	1	1	1	1	1	1	1	1	1	1	1	1
K01637	1	1	1	1	1	1	1	1	1	1	1	1	1	1	1	1	1	1	1	1	1	1	1	1	1	1
K01638	1	1	1	1	1	1	2	1	1	1	1	1	1	1	1	1	1	1	1	1	1	1	1	1	1	1
K01640	1	1	1	1	1	1	1	1	1	1	1	1	1	1	1	1	1	1	1	1	1	1	1	1	1	1
K01644	0	0	1	1	0	0	1	1	0	0	0	0	0	1	0	0	0	0	0	1	1	0	0	0	0	0
K01647	1	1	1	1	1	1	1	1	1	1	1	1	1	1	1	1	1	1	1	1	1	1	1	1	1	1
K01649	1	1	1	1	1	1	1	1	1	1	1	1	1	1	1	1	1	1	1	1	1	1	1	1	1	1
K01652	2	1	2	1	2	2	1	1	2	2	2	2	2	2	2	3	2	3	3	2	1	1	1	2	2	2
K01653	1	1	1	1	1	1	1	1	1	1	1	1	1	1	1	1	1	1	1	1	1	1	1	1	1	1
K01654	1	2	0	0	0	0	1	0	1	0	1	0	1	0	1	0	0	0	1	0	0	1	1	1	1	0
K01657	1	1	1	1	2	2	1	1	2	1	1	1	2	2	2	2	2	1	1	2	1	1	1	2	2	2
K01658	1	1	1	1	1	1	1	1	1	2	1	1	1	1	1	1	1	0	0	1	1	0	0	1	1	1
K01659	1	1	1	1	1	1	1	1	1	1	1	1	1	1	1	1	1	1	1	1	1	1	1	1	1	1
K01662	1	1	1	1	1	1	1	1	1	1	1	1	1	1	1	1	1	1	1	1	1	1	1	1	1	1
K01664	1	1	1	1	1	1	1	1	1	0	1	1	1	1	1	1	1	1	1	1	1	1	1	1	1	1
K01665	0	1	1	1	1	1	1	1	1	1	1	1	1	1	1	1	1	1	1	1	1	1	1	1	1	1
K01666	0	0	0	0	0	0	0	0	0	0	0	0	0	0	0	1	0	0	0	0	0	0	0	0	0	0
K01669	2	1	1	1	2	2	1	1	2	1	2	2	2	2	2	2	2	1	1	2	1	2	2	2	2	2
K01673	1	1	1	1	2	1	1	1	1	2	1	1	2	2	1	2	1	1	1	1	1	1	1	1	1	1
K01674	0	0	0	1	0	0	0	0	0	0	0	0	0	0	0	0	0	1	1	0	0	0	0	0	0	0
K01679	1	1	1	1	1	1	1	1	1	1	1	1	1	1	1	1	1	1	1	1	1	1	1	0	1	1
K01681	2	2	2	2	2	2	2	2	2	2	2	2	2	2	2	2	2	2	2	2	2	2	2	2	2	2
K01682	1	1	1	1	1	1	1	1	1	1	1	1	1	1	1	1	1	1	1	1	1	1	1	1	1	1
K01687	1	1	1	1	1	1	1	1	1	1	1	1	1	1	2	1	1	1	1	1	1	1	1	1	1	2
K01689	1	1	1	1	1	1	1	1	1	1	1	1	1	1	1	1	1	1	1	1	1	1	1	1	1	1
K01692	0	0	0	1	0	0	0	0	0	0	1	0	1	0	2	0	0	1	1	0	0	0	0	0	0	0
K01693	1	1	1	1	1	1	1	1	1	1	1	1	1	1	1	1	1	1	1	1	1	1	1	1	1	1
K01695	1	1	1	1	1	1	1	1	1	1	1	1	1	1	1	1	1	1	1	1	1	1	1	0	1	1
K01696	1	1	1	1	1	1	1	1	1	1	1	1	1	1	1	1	1	1	1	1	1	1	1	1	1	1
K01698	1	1	1	1	1	1	1	1	1	1	1	1	1	1	2	1	1	1	1	1	1	1	1	1	1	1
K01703	1	1	1	1	1	1	1	1	1	1	1	1	1	1	1	1	1	1	1	1	1	1	1	1	1	1
K01704	1	1	1	1	1	1	1	1	0	1	1	1	1	1	1	1	1	1	1	1	1	1	1	0	0	1
K01710	0	1	1	1	0	0	0	0	0	1	0	0	0	0	1	0	0	0	0	0	0	0	0	0	0	0
K01711	0	0	0	0	0	0	0	0	0	0	0	2	0	0	0	0	0	0	0	0	0	0	0	0	0	0
K01712	1	0	1	0	1	1	0	1	1	1	1	1	1	1	1	1	1	1	1	1	1	1	1	1	1	0
K01714	1	1	2	2	1	1	1	1	2	2	1	1	1	2	1	2	2	2	2	1	1	1	1	2	2	2
K01719	1	1	1	1	1	1	1	1	1	1	1	1	1	1	1	1	1	1	1	1	1	1	1	1	1	1
K01720	0	1	1	0	1	1	0	0	1	1	1	1	1	1	1	1	1	1	1	1	0	1	1	1	1	0
K01724	2	1	2	1	2	2	1	2	1	2	2	2	2	2	2	2	2	2	2	2	2	1	1	1	1	2
K01725	0	0	0	0	1	0	0	0	0	1	0	0	1	1	0	1	0	0	0	0	0	0	0	0	0	0
K01733	1	1	1	1	1	1	1	1	1	1	1	1	1	1	1	1	1	1	1	1	1	1	1	1	1	1
K01735	1	1	1	1	1	1	1	1	1	1	1	1	1	1	1	1	1	1	1	1	1	1	1	1	1	1
K01736	1	1	1	1	1	1	1	1	1	1	1	1	1	1	1	1	1	1	1	1	1	1	1	1	1	1
K01737	1	1	1	1	1	1	1	1	1	1	1	1	1	1	1	1	1	1	1	1	1	0	0	1	1	1
K01740	1	0	1	1	1	1	1	1	1	1	1	1	1	1	1	1	1	1	1	1	1	1	1	0	1	1

K01744	1	0	1	1	0	1	1	1	0	1	0	0	0	0	0	0	1	0	0	0	1	0	0	0	0	1
K01745	1	0	1	0	1	1	0	1	1	1	1	1	1	1	1	1	1	1	1	1	1	1	1	1	1	1
K01749	1	1	1	1	1	1	1	1	1	1	1	1	1	1	1	1	1	1	1	1	1	1	1	1	1	1
K01750	1	1	3	1	1	0	1	1	1	3	1	1	1	1	1	1	1	2	2	2	1	0	0	1	1	1
K01751	0	0	1	0	0	0	0	0	0	1	0	0	0	0	0	0	0	0	1	0	0	0	0	0	0	0
K01752	1	1	1	1	1	1	1	1	1	1	1	1	1	1	1	1	1	1	1	1	1	1	1	1	1	1
K01753	1	0	0	1	0	0	1	1	0	0	0	0	0	0	0	0	0	0	0	0	0	0	0	0	0	0
K01754	1	2	2	1	1	1	1	1	1	2	1	1	1	1	1	1	1	1	1	1	1	1	1	1	1	1
K01755	1	1	1	1	1	1	1	1	1	1	1	1	1	1	1	1	1	1	1	1	1	1	1	1	1	1
K01756	1	1	1	1	1	1	1	1	1	1	1	1	1	1	1	1	1	1	1	1	1	1	1	1	1	1
K01759	1	1	1	1	1	1	1	1	1	1	1	1	1	1	2	1	2	1	1	1	1	1	1	1	1	1
K01760	0	0	0	0	0	0	0	0	0	0	0	0	0	0	0	1	0	0	0	0	0	0	0	0	0	0
K01770	1	1	1	1	1	1	1	1	1	1	1	1	1	1	1	1	1	1	1	1	1	1	1	1	1	1
K01772	1	1	1	1	1	1	1	1	1	1	1	1	1	1	1	1	1	1	1	1	1	1	1	1	1	1
K01775	1	1	1	1	1	1	1	1	1	1	1	1	1	1	1	1	1	1	1	1	1	1	1	1	1	1
K01776	1	1	1	1	1	1	1	1	1	1	1	0	1	1	1	1	1	1	1	1	1	1	1	1	1	1
K01778	1	1	1	1	1	1	1	1	1	1	1	1	1	1	1	1	1	1	1	1	1	1	1	1	1	1
K01779	0	0	1	1	1	0	0	0	1	1	1	1	1	1	1	1	1	1	1	1	0	1	1	1	1	1
K01782	1	1	1	1	1	1	1	1	1	1	1	0	1	1	1	1	1	1	1	1	1	1	1	1	1	1
K01783	1	1	1	1	1	1	1	1	1	1	1	1	1	1	1	1	1	1	1	1	1	1	1	1	1	1
K01784	0	1	2	0	1	0	1	1	1	1	2	2	1	2	1	3	2	1	1	0	1	1	1	1	1	0
K01790	0	1	1	0	0	0	0	0	0	0	0	0	0	0	0	0	0	0	0	0	0	0	0	0	0	0
K01791	0	0	0	1	1	0	0	0	1	0	0	0	0	1	1	0	1	0	0	1	0	0	0	1	1	1
K01792	0	0	0	1	0	0	1	1	0	0	0	0	0	0	0	0	0	0	0	0	1	0	0	0	0	0
K01799	0	0	0	0	0	0	0	0	0	0	0	0	0	0	0	1	0	0	1	0	0	0	0	0	0	0
K01801	1	0	1	0	1	1	0	1	0	1	1	1	1	1	1	1	1	1	1	1	2	0	0	0	0	1
K01803	1	1	1	1	1	1	1	1	1	1	1	1	1	1	1	1	1	1	1	1	1	1	1	1	1	1
K01807	1	1	1	1	1	1	1	1	1	1	1	1	1	1	1	1	1	1	1	1	1	1	1	1	1	1
K01810	1	1	1	1	1	1	1	1	1	1	1	1	1	1	1	1	1	1	1	1	1	1	1	1	1	1
K01814	1	1	1	1	1	1	1	1	1	1	1	1	1	1	1	1	1	1	1	1	1	1	1	1	1	1
K01817	1	1	1	1	1	1	1	1	1	1	1	1	1	1	1	1	1	1	1	1	1	1	1	1	1	1
K01825	1	1	1	1	1	1	1	1	1	1	1	1	1	1	1	1	1	1	1	1	1	1	1	1	1	1
K01826	2	1	1	1	1	1	1	1	1	1	1	1	1	1	1	1	1	1	1	1	1	1	1	1	1	1
K01840	1	1	1	1	1	1	1	1	1	1	1	1	1	1	1	1	1	1	1	1	1	1	1	1	1	1
K01845	1	1	1	1	1	1	1	1	1	1	1	1	1	1	1	1	1	1	1	1	1	1	1	1	1	1
K01856	1	0	1	0	1	0	0	0	0	1	0	0	0	0	0	1	0	1	1	0	0	1	1	0	0	1
K01857	0	0	0	1	1	0	0	0	0	0	1	0	1	0	1	0	0	1	1	0	0	0	0	0	0	0
K01866	1	1	1	1	1	1	1	1	1	1	1	1	1	1	1	1	1	1	1	1	1	1	1	1	1	1
K01867	1	1	1	1	1	1	1	1	1	1	1	1	1	1	1	1	1	1	1	1	1	1	1	1	1	1
K01868	1	1	1	1	1	1	1	1	1	1	1	1	1	1	1	1	1	1	1	1	1	1	1	1	1	1
K01869	1	1	1	1	1	1	1	1	1	1	0	0	1	1	1	1	1	1	1	1	1	1	1	0	1	1
K01870	1	1	1	1	1	1	1	1	1	1	1	1	1	1	1	1	1	1	1	1	1	1	1	1	1	1
K01872	1	1	1	1	1	1	1	1	1	1	1	1	1	1	1	1	1	1	1	1	1	1	1	1	1	1
K01873	1	1	1	1	1	1	1	1	1	1	0	1	1	1	1	1	1	1	1	1	1	1	1	1	1	1
K01874	1	1	1	1	1	1	1	1	1	1	1	1	1	1	1	1	1	1	1	1	1	1	1	1	1	1
K01875	1	1	1	1	1	1	1	1	1	1	1	1	1	1	1	1	1	1	1	1	1	1	1	1	1	1
K01876	1	1	1	1	1	1	1	1	1	1	1	1	1	1	1	1	1	1	1	1	1	1	1	1	1	1
K01878	1	1	1	1	1	1	1	1	1	1	1	1	1	1	1	1	1	1	1	1	1	1	1	1	1	1
K01879	1	1	1	1	1	1	1	1	1	1	1	1	1	1	1	1	1	1	1	1	1	1	1	1	1	1
K01881	1	1	1	1	1	1	1	1	1	1	1	1	1	1	1	1	1	1	1	1	1	1	1	1	1	1
K01883	1	1	1	1	1	1	1	1	1	1	1	1	1	1	1	1	1	1	1	1	1	1	1	1	1	1
K01885	1	1	1	1	1	1	1	1	1	1	1	1	1	1	1	1	1	1	1	1	1	1	1	1	1	1
K01886	1	1	1	1	1	1	1	1	1	1	1	1	1	1	1	1	1	1	1	1	1	1	1	1	1	1
K01887	1	1	1	1	1	1	1	1	1	1	1	1	1	1	1	1	1	1	1	1	1	1	1	1	1	1
K01889	1	1	1	1	1	1	1	1	1	1	1	1	1	1	1	1	1	1	1	1	1	1	1	1	1	1
K01890	1	1	1	1	1	1	1	1	1	1	1	1	1	1	1	1	1	1	1	1	1	1	1	1	1	1
K01892	1	1	1	1	1	1	1	1	1	1	1	1	1	1	1	1	1	1	1	1	1	1	1	1	1	1
K01894	1	1	1	1	1	1	1	1	1	1	1	1	1	1	1	1	1	1	1	1	1	1	1	1	1	1
K01895	2	2	2	2	2	2	2	2	2	2	2	2	2	2	2	2	2	2	2	2	2	1	1	2	2	2
K01897	2	2	2	2	3	2	3	3	3	2	3	3	3	3	2	3	3	3	3	3	2	2	2	3	3	2
K01902	1	1	1	1	1	1	1	1	1	1	1	1	1	1	1	1	1	1	1	1	1	1	1	1	1	1
K01903	1	1	1	1	1	1	1	1	1	1	1	1	1	1	1	1	1	1	1	1	1	1	1	1	1	1
K01908	1	1	1	1	1	1	1	1	1	1	1	1	1	1	1	1	1	1	1	1	1	0	0	1	1	1
K01912	0	0	0	1	0	1	0	0	0	0	1	0	1	0	1	0	0	0	0	0	0	0	0	0	0	0
K01915	1	1	1	2	2	1	1	1	1	2	2	1	2	1	1	2	1	2	2	2	1	1	1	1	1	1
K01918	1	1	1	1	1	1	1	1	1	1	1	1	1	1	1	1	1	1	1	1	1	1	1	1	1	1
K01919	1	1	1	1	1	1	1	1	1	1	1	1	1	1	1	1	1	1	1	1	1	1	1	1	1	1
K01920	1	1	1	1	1	1	1	1	1	1	1	1	1	1	1	1	1	1	1	1	1	1	1	1	1	1

K01921	1	1	1	1	1	1	1	2	1	1	1	1	1	0	1	1	1	1	1	1	1	1	1	1	1	1
K01923	1	1	1	1	1	1	1	1	1	1	1	1	1	1	1	1	1	1	1	1	1	1	1	1	1	1
K01924	1	1	1	1	1	1	1	1	1	1	1	1	1	1	1	1	1	1	1	1	1	1	1	1	1	1
K01925	1	1	1	1	1	1	1	1	1	1	1	1	1	1	1	1	1	1	1	1	1	1	1	0	1	1
K01928	1	1	1	1	1	1	1	1	1	1	1	1	1	1	1	1	1	1	1	1	1	1	1	0	1	1
K01929	1	1	1	1	1	1	1	1	1	1	1	1	1	1	1	1	1	1	1	1	1	1	1	1	1	1
K01933	1	1	1	1	1	1	1	1	1	1	1	1	1	1	1	1	1	1	1	1	1	1	1	1	1	1
K01934	1	1	1	1	1	1	1	1	1	1	1	1	1	1	1	1	1	1	1	1	1	1	1	1	1	1
K01935	1	1	1	1	1	1	2	2	1	1	1	1	1	1	2	1	1	1	1	1	2	1	1	1	1	1
K01937	1	1	1	1	1	1	1	1	1	1	1	1	1	1	1	1	1	1	1	1	1	1	1	1	1	1
K01938	0	0	0	1	0	0	1	1	0	0	0	0	0	0	0	0	0	1	1	0	1	1	1	0	0	0
K01939	1	1	1	1	1	1	1	1	1	1	1	1	1	1	1	1	1	1	1	1	1	1	1	1	1	1
K01940	1	1	1	1	1	1	1	1	1	1	1	1	1	1	1	1	1	1	1	1	1	1	1	1	1	1
K01941	1	0	0	0	0	0	0	0	0	0	0	0	0	0	0	0	0	0	0	0	0	0	0	0	0	0
K01945	1	1	1	1	1	1	1	1	1	1	1	1	1	1	1	1	1	1	1	1	1	1	1	1	1	1
K01950	1	1	1	1	1	1	1	1	1	1	1	1	1	1	1	1	1	1	1	1	1	1	1	1	1	1
K01951	1	1	1	1	2	2	1	1	1	1	1	1	1	2	1	2	1	1	1	2	1	2	2	1	1	2
K01952	1	1	1	1	1	1	1	1	1	1	0	1	1	1	1	1	1	1	1	1	1	1	1	1	1	1
K01953	0	2	1	0	0	1	0	0	0	1	0	0	0	0	1	0	0	0	0	0	1	0	0	0	0	0
K01955	1	1	1	1	1	1	1	1	1	1	1	1	0	1	1	1	1	2	2	1	1	1	1	1	1	1
K01956	1	1	1	1	1	1	1	1	1	1	1	1	1	1	1	1	1	1	1	1	1	1	1	1	1	1
K01961	1	2	1	1	1	1	1	1	2	1	2	1	1	1	2	1	2	2	2	2	2	2	2	2	2	2
K01962	1	1	1	1	1	1	1	1	1	1	1	1	1	1	1	1	1	1	1	1	1	1	1	1	1	1
K01963	1	1	1	1	1	1	1	1	1	1	1	1	1	1	1	1	1	1	1	1	1	1	1	1	1	1
K01968	1	1	1	1	1	1	1	1	1	1	1	1	1	1	1	1	1	1	1	1	1	1	1	1	1	1
K01969	1	1	1	1	1	1	1	1	1	1	1	1	1	1	1	1	1	1	1	1	1	1	1	1	1	1
K01972	1	1	1	1	1	1	1	1	1	1	1	1	1	1	1	1	1	1	1	1	1	1	1	1	1	1
K01974	1	0	0	0	1	1	0	0	0	0	0	0	1	1	0	1	0	1	0	0	0	0	0	0	0	1
K01989	2	2	2	2	2	2	2	2	2	2	2	1	2	2	2	2	2	2	2	2	2	2	2	2	2	2
K01990	1	1	1	1	1	1	1	1	1	1	1	1	1	1	1	1	1	1	1	1	1	1	1	1	1	1
K01991	1	1	1	1	1	1	1	1	0	1	1	1	0	1	1	1	1	1	1	0	1	1	1	0	0	1
K01992	1	1	3	4	3	3	4	3	3	3	3	3	3	3	1	3	3	3	3	4	3	3	3	2	3	3
K01993	0	0	1	2	1	1	2	1	1	1	1	0	0	0	0	1	1	1	1	2	1	1	1	1	1	1
K01995	0	0	0	0	0	0	1	0	0	0	0	0	0	0	0	0	1	0	0	1	0	0	0	0	0	0
K01996	0	0	0	0	0	0	1	0	0	0	0	0	0	0	0	0	1	0	0	1	0	0	0	0	0	0
K01997	0	0	0	0	0	0	1	0	0	0	1	0	0	0	0	0	1	0	0	1	0	0	0	0	0	0
K01998	0	0	0	0	0	0	1	0	0	0	1	0	0	0	0	0	1	0	0	1	0	0	0	0	0	0
K01999	0	0	0	0	0	0	1	0	0	0	1	0	0	0	0	0	1	0	0	1	0	0	0	0	0	0
K02000	1	0	1	1	1	1	1	1	1	1	1	1	1	1	1	1	1	1	1	1	1	1	1	1	1	1
K02002	1	0	1	1	1	1	1	1	1	1	1	1	1	1	1	1	1	1	1	1	1	1	1	1	1	1
K02003	1	1	1	1	1	1	1	1	1	1	1	1	1	1	2	1	1	1	1	1	1	1	1	1	1	1
K02004	1	1	1	1	1	1	1	1	1	1	1	1	1	1	2	1	1	1	1	1	1	1	1	1	1	1
K02005	0	0	0	0	1	0	0	0	0	0	0	0	1	1	2	1	0	1	1	0	0	0	0	0	0	0
K02010	1	1	1	1	1	1	1	1	1	1	1	1	2	2	2	3	1	2	2	2	1	1	1	1	1	1
K02011	1	1	1	1	1	1	1	1	1	1	2	0	2	1	2	3	1	2	2	2	1	1	1	1	1	1
K02012	1	1	1	2	1	1	2	2	1	1	2	1	2	2	2	3	1	2	2	2	2	1	1	1	1	1
K02013	3	0	2	4	3	2	3	2	2	4	2	3	3	3	0	5	2	1	1	3	4	3	3	2	2	5
K02014	4	0	1	3	3	3	4	3	3	2	3	1	5	3	0	7	4	0	0	4	5	3	3	3	3	4
K02015	5	0	4	6	4	3	4	3	3	6	3	4	3	3	0	6	3	2	2	5	5	4	4	3	3	7
K02016	3	0	2	4	2	2	3	2	2	4	2	2	3	2	0	5	2	1	1	3	3	3	3	2	2	4
K02018	1	1	1	1	1	1	1	1	1	1	1	1	1	1	1	1	1	2	2	1	1	1	1	1	1	1
K02020	1	1	1	1	1	1	1	1	1	1	1	1	1	1	1	1	1	1	1	1	1	1	1	1	1	1
K02022	1	1	0	0	0	0	0	1	0	0	1	1	0	0	1	0	0	0	0	0	0	1	1	0	0	0
K02028	1	1	1	1	1	1	1	1	1	1	1	1	1	1	1	1	1	1	1	1	1	1	1	1	1	1
K02029	2	2	2	2	2	2	2	2	2	2	2	2	2	2	2	2	2	2	2	2	2	2	2	2	2	3
K02030	3	2	3	3	3	3	3	3	3	3	3	3	3	3	3	3	3	3	3	3	3	3	3	3	3	4
K02031	0	0	0	1	0	0	1	0	0	0	0	0	0	0	0	0	0	0	0	0	0	0	0	0	0	0
K02032	1	0	0	1	1	0	1	0	0	0	0	0	0	0	0	0	0	0	0	0	0	0	0	0	0	1
K02033	1	0	0	1	1	0	1	0	0	0	0	0	0	0	0	0	0	0	0	0	0	0	0	0	0	1
K02034	1	0	0	1	1	0	1	0	0	0	0	0	0	0	0	0	0	0	0	0	0	0	0	0	0	1
K02035	2	1	1	2	2	1	2	1	1	0	1	1	1	1	1	1	1	1	1	1	1	1	1	1	1	2
K02036	1	1	1	1	1	1	1	1	1	1	1	1	1	1	1	1	1	1	1	1	1	1	1	1	1	1
K02037	1	1	1	1	1	1	1	1	1	1	1	1	1	1	1	1	1	1	1	1	1	1	1	1	1	1
K02038	1	1	1	1	1	1	1	1	1	1	1	1	1	1	1	1	1	1	1	1	1	1	1	1	1	1
K02039	1	1	1	1	1	1	1	1	1	1	1	1	1	1	1	1	1	1	1	1	1	1	1	1	1	1
K02040	1	1	1	1	1	1	1	1	1	1	0	0	1	1	1	1	1	1	1	1	1	1	1	1	1	1
K02044	1	0	0	0	1	1	0	0	1	0	1	1	1	1	1	1	1	1	1	1	0	1	1	1	1	1
K02045	1	1	1	1	1	1	1	1	1	1	1	1	1	1	1	1	1	1	1	1	1	1	1	1	1	1

K02046	1	1	1	1	1	1	1	1	1	1	1	1	1	1	1	1	1	1	1	1	1	1	1	1	1	1
K02047	1	1	1	1	1	1	1	1	1	1	1	1	1	1	1	1	1	1	1	1	1	1	1	1	1	1
K02048	2	2	2	1	2	2	2	2	2	2	2	2	2	2	2	2	2	2	2	2	2	1	1	2	2	2
K02049	1	0	0	0	0	0	0	0	0	0	0	0	0	0	0	0	0	0	0	0	0	0	0	0	0	0
K02050	1	0	0	0	0	0	0	0	0	0	0	0	0	0	0	0	0	0	0	0	0	0	0	0	0	0
K02051	1	0	0	0	0	0	0	0	0	0	0	0	0	0	0	0	0	0	0	0	0	0	0	0	0	0
K02068	0	0	0	0	1	1	0	0	1	0	1	1	1	1	1	1	1	1	1	1	0	0	0	1	1	1
K02069	1	0	0	0	1	1	0	0	1	0	1	1	1	1	1	1	1	1	1	1	0	0	0	1	1	1
K02071	1	1	1	1	1	1	1	1	1	1	0	0	1	1	1	1	1	1	1	1	1	1	1	1	1	1
K02072	1	1	1	1	1	1	0	1	1	1	1	1	1	1	1	1	1	1	1	1	1	1	1	1	1	1
K02073	1	1	1	1	1	1	1	1	1	1	1	1	1	1	1	1	1	1	1	1	1	1	1	1	1	1
K02074	0	0	0	0	0	0	0	0	0	0	0	0	0	0	1	0	0	0	0	0	0	0	0	0	0	0
K02075	0	0	0	0	0	0	0	0	0	0	0	0	0	0	1	0	0	0	0	0	0	0	0	0	0	0
K02077	0	0	0	0	0	0	0	0	0	0	0	0	0	0	1	0	0	0	0	0	0	0	0	0	0	0
K02078	1	1	1	1	1	1	1	1	1	1	1	1	1	1	1	1	1	1	1	1	1	1	1	1	1	1
K02106	0	0	1	0	1	0	0	0	1	1	1	1	1	1	1	1	1	1	1	1	1	0	0	1	1	1
K02108	1	1	1	1	1	1	1	1	1	1	1	1	1	1	1	1	1	1	1	1	1	1	1	1	1	1
K02109	1	1	1	1	1	1	1	1	1	1	1	1	1	1	1	1	1	1	1	1	1	1	1	1	1	1
K02110	1	1	1	1	1	1	1	1	1	1	1	1	1	1	1	1	1	1	1	1	1	1	1	1	1	1
K02111	1	1	1	1	1	1	1	1	1	1	1	1	1	1	1	1	1	1	1	1	1	1	1	1	1	1
K02112	1	1	1	1	1	1	1	1	1	1	1	1	1	1	1	1	1	1	1	1	1	1	1	1	1	1
K02113	1	1	1	1	1	1	1	1	1	1	1	1	1	1	1	1	1	1	1	1	1	1	1	1	1	1
K02114	1	1	1	1	1	1	1	1	1	1	1	1	1	1	1	1	1	1	1	1	1	1	1	1	1	1
K02115	1	1	1	1	1	1	1	1	1	1	1	1	1	1	1	1	1	1	1	1	1	1	1	1	1	1
K02116	1	1	1	1	1	1	1	1	1	1	1	1	1	1	1	1	1	1	1	1	1	1	1	1	1	1
K02160	1	3	1	1	1	1	1	1	3	1	3	1	1	1	2	1	3	3	2	3	2	3	3	3	3	3
K02168	3	3	4	2	4	3	2	3	5	4	5	5	4	4	4	4	5	5	6	4	2	3	3	5	5	3
K02169	1	1	1	0	1	1	0	0	1	1	1	1	1	1	1	1	1	1	1	1	0	0	0	1	1	1
K02192	1	1	1	1	1	1	1	1	1	1	1	1	1	1	1	1	1	1	1	1	1	1	1	1	1	1
K02193	1	1	1	1	1	1	1	1	1	1	1	1	1	1	1	1	1	1	1	1	1	1	1	1	1	1
K02194	1	1	1	1	1	1	1	1	1	1	1	1	1	1	1	1	1	1	1	1	1	1	1	1	1	1
K02195	1	1	1	1	1	1	1	1	1	1	1	1	1	1	1	1	1	1	1	1	1	1	1	1	1	0
K02196	1	1	1	1	1	1	1	1	1	1	1	1	1	0	1	1	1	1	1	1	1	1	1	1	1	1
K02197	1	1	1	1	1	1	1	1	1	1	1	1	1	1	1	1	1	1	1	1	1	1	1	1	1	1
K02198	1	1	2	1	2	2	1	1	2	2	2	1	2	2	1	2	3	3	2	1	1	2	1	2	2	1
K02199	1	1	2	1	2	2	1	1	2	2	2	1	2	2	1	2	3	3	2	1	1	2	1	2	2	2
K02200	1	1	2	1	2	2	1	1	2	2	0	0	2	2	1	2	3	3	2	1	1	2	1	2	2	2
K02203	0	0	0	0	0	0	0	0	0	0	0	0	0	0	0	1	0	0	0	3	0	0	0	0	0	0
K02204	1	1	1	1	1	1	1	1	1	1	1	1	1	1	1	1	1	1	1	1	1	1	1	1	1	1
K02217	0	0	0	0	0	0	1	1	0	0	0	0	0	0	0	0	0	0	0	0	1	0	0	0	0	0
K02221	1	1	1	1	1	1	1	1	1	1	1	1	1	1	1	1	1	1	1	1	1	1	1	1	1	1
K02226	1	0	1	1	1	0	1	1	1	1	0	0	1	1	0	1	0	1	1	0	1	0	0	1	1	1
K02231	0	0	0	1	0	0	1	1	0	0	0	0	0	0	0	0	0	0	0	0	0	0	0	0	0	0
K02233	1	0	1	1	1	0	1	1	1	1	0	0	1	1	0	1	0	1	1	0	1	0	0	1	1	1
K02237	1	1	1	1	1	1	1	1	1	1	1	1	1	1	1	1	1	1	1	1	1	1	1	1	1	1
K02238	1	1	1	1	1	1	1	0	1	1	1	1	1	1	1	1	1	1	1	1	1	1	1	1	1	0
K02279	0	0	0	1	0	0	0	0	0	0	0	0	0	0	0	0	0	0	0	0	0	0	0	0	0	0
K02280	0	0	0	1	0	0	0	0	0	0	0	0	0	0	0	0	0	0	0	0	0	0	0	0	0	0
K02283	0	0	0	1	0	0	0	0	0	0	0	0	0	0	0	0	0	0	0	0	0	0	0	0	0	0
K02294	0	0	0	0	1	0	0	0	1	0	0	0	1	1	0	1	0	0	0	1	0	0	0	1	1	0
K02297	0	0	0	0	0	0	0	0	0	0	0	0	0	0	0	0	0	0	0	0	0	0	0	0	0	1
K02298	0	0	0	0	0	0	0	0	0	0	0	0	0	0	0	0	0	0	0	0	0	0	0	0	0	1
K02299	0	0	0	0	0	0	0	0	0	0	0	0	0	0	0	0	0	0	0	0	0	0	0	0	0	1
K02301	0	0	0	0	0	0	0	0	0	0	0	0	0	0	0	0	0	0	0	0	0	0	0	0	0	1
K02302	1	1	1	1	1	1	1	1	1	1	1	1	1	1	1	1	1	1	1	1	1	1	1	1	1	1
K02303	1	0	1	1	0	0	1	1	1	1	0	1	0	0	0	0	1	0	0	1	1	0	0	1	1	1
K02313	1	1	1	1	1	1	1	1	1	1	1	1	1	1	1	1	1	1	1	1	1	1	1	1	1	1
K02314	1	1	1	1	1	1	1	1	1	1	1	2	1	1	1	2	1	1	1	1	1	1	1	1	1	2
K02315	0	0	0	0	0	0	0	0	0	0	0	0	0	0	0	1	0	0	0	0	0	0	0	0	0	0
K02316	1	1	1	1	1	1	1	1	1	1	1	1	1	1	1	1	1	1	1	1	1	1	1	1	1	1
K02335	1	1	1	1	1	1	1	1	0	1	1	1	1	1	1	1	1	1	1	1	1	1	1	0	1	1
K02337	1	1	1	1	1	1	1	1	1	1	1	1	1	1	1	1	1	1	1	1	1	1	1	1	1	1
K02338	1	1	1	1	1	1	1	1	1	1	1	1	1	1	1	1	1	1	1	1	1	1	1	1	1	1
K02339	1	1	1	1	1	1	1	1	1	1	1	1	1	1	1	1	1	1	1	1	1	1	1	1	1	1
K02340	1	1	1	1	1	1	1	1	1	1	1	1	1	1	1	1	1	1	1	1	1	1	1	1	1	1
K02341	1	1	1	1	1	1	1	1	1	1	1	1	1	1	1	1	1	1	1	1	1	1	1	1	1	1
K02342	1	1	1	1	1	1	1	1	1	1	1	1	2	1	1	1	1	2	1	1	1	1	1	1	1	1
K02343	1	1	1	1	1	0	1	1	1	1	0	0	0	1	1	1	1	1	1	1	1	1	1	1	1	0

K02346	1	0	1	1	1	1	1	1	0	1	1	1	1	1	1	1	1	1	1	1	1	1	1	0	0	1
K02348	1	1	1	1	1	1	1	1	1	1	1	1	1	1	1	1	1	1	1	1	1	1	1	1	1	2
K02355	1	1	1	1	1	1	1	1	1	1	1	1	1	1	1	1	1	1	1	1	1	1	1	1	1	1
K02356	1	1	1	1	1	1	1	1	1	1	1	1	1	1	1	1	1	1	1	1	1	1	1	1	1	1
K02357	1	1	1	1	1	1	1	1	1	1	0	1	1	1	1	1	1	1	1	1	1	1	1	1	1	1
K02358	0	2	2	2	2	1	2	1	4	2	0	1	0	0	2	2	2	2	2	1	2	4	4	4	4	1
K02361	0	0	0	0	0	0	0	0	0	0	0	0	0	0	0	1	0	0	0	1	0	0	0	0	0	0
K02363	0	0	0	0	0	0	0	0	0	0	0	0	0	0	0	1	0	0	0	1	0	0	0	0	0	0
K02372	1	1	1	1	1	1	1	1	1	1	1	1	1	1	1	1	1	1	1	1	1	1	1	1	1	1
K02377	0	0	0	0	0	0	0	0	0	0	0	1	0	0	0	0	0	0	0	0	0	0	0	0	0	0
K02379	1	1	1	1	1	1	1	0	1	1	1	1	1	1	1	1	1	1	1	1	1	1	1	1	1	1
K02424	2	0	2	1	2	2	0	1	1	2	2	2	2	2	2	2	2	4	4	2	1	1	1	1	1	2
K02427	1	1	1	1	1	1	1	1	1	1	1	1	1	1	1	1	1	1	1	1	1	1	1	1	1	1
K02428	1	1	1	1	1	1	1	1	1	1	1	1	1	1	1	1	1	1	1	1	1	1	1	1	1	1
K02433	1	1	1	1	1	1	2	1	1	1	1	1	1	1	2	1	1	2	2	1	1	1	1	1	1	1
K02434	1	1	1	1	1	1	1	1	1	1	1	1	1	1	1	1	1	1	1	1	1	1	1	1	1	1
K02435	1	1	1	1	1	1	1	1	1	1	1	1	1	1	1	1	1	1	1	1	1	1	1	1	1	1
K02437	1	1	1	1	1	1	1	1	1	1	1	1	1	1	1	1	1	1	1	1	1	1	1	1	1	1
K02442	0	0	1	0	2	1	0	0	1	1	1	1	1	1	0	1	1	1	1	1	0	0	0	1	1	1
K02452	1	0	1	0	1	1	0	0	1	1	1	1	1	1	1	1	1	1	1	1	0	1	1	1	1	0
K02453	1	0	1	0	1	1	0	0	1	1	1	1	1	1	1	1	1	1	1	1	0	1	1	1	1	1
K02454	2	1	2	0	2	2	0	0	2	2	2	2	2	2	2	2	2	2	2	4	0	2	2	2	2	1
K02455	1	0	1	0	1	1	0	0	1	1	1	1	1	1	1	1	1	1	1	1	0	1	1	1	1	1
K02456	1	0	1	0	1	1	0	0	1	1	1	1	1	1	1	1	1	1	1	1	0	1	1	1	1	1
K02458	1	0	1	0	1	1	0	0	1	1	1	1	1	1	1	1	1	1	1	3	0	1	1	0	1	1
K02459	1	0	1	0	1	1	0	0	1	1	1	1	1	1	1	1	1	1	1	3	0	1	1	1	1	1
K02460	1	0	1	0	1	1	0	0	1	1	1	1	1	1	1	1	1	1	1	3	0	1	1	1	1	1
K02461	1	0	1	0	1	1	0	0	1	1	1	1	1	1	1	1	1	1	1	1	0	1	1	1	1	1
K02463	1	0	1	0	1	1	0	0	1	1	1	1	1	1	1	1	1	1	1	1	0	1	1	1	1	1
K02469	1	1	1	1	1	1	1	1	1	1	1	1	1	1	1	1	1	1	1	1	1	1	1	1	1	1
K02470	1	1	1	1	1	1	1	1	1	1	1	1	1	1	1	1	1	1	1	1	1	1	1	1	1	1
K02472	0	0	0	0	1	0	0	0	1	0	0	0	0	0	0	0	1	0	0	1	0	0	0	1	1	1
K02473	1	1	0	0	0	1	0	0	0	1	0	0	0	0	1	0	0	1	1	0	1	1	1	0	0	1
K02474	1	1	1	1	0	1	0	0	0	1	0	0	0	1	1	2	0	1	1	0	1	1	1	0	0	1
K02478	0	0	1	0	0	0	0	0	0	0	0	0	0	0	0	0	0	1	1	0	0	0	0	0	0	0
K02483	1	0	0	0	0	1	0	0	1	0	0	0	0	0	0	0	0	1	1	0	0	0	0	1	1	1
K02484	0	0	0	0	0	1	0	0	1	0	0	0	0	0	0	0	0	1	1	0	0	0	0	1	1	0
K02492	1	1	1	1	1	1	1	1	1	1	1	1	1	1	1	1	1	1	1	1	1	1	1	1	1	1
K02493	1	1	1	1	1	1	1	1	1	1	1	1	1	1	1	1	1	1	1	1	1	1	1	1	1	1
K02494	1	1	1	1	1	1	1	1	1	1	1	1	1	1	1	1	1	1	1	1	1	1	1	1	1	1
K02495	2	2	2	2	2	2	2	2	2	2	2	2	2	2	2	2	2	2	2	2	2	2	2	2	2	2
K02500	1	1	1	1	1	1	1	1	1	1	1	1	1	1	1	1	1	1	1	1	1	1	1	1	1	1
K02501	1	1	1	1	1	1	1	1	1	1	1	1	1	1	1	1	1	1	1	1	1	1	1	1	1	1
K02502	1	1	1	1	1	1	1	1	1	1	1	1	1	1	1	1	1	1	1	1	1	1	1	1	1	1
K02503	1	1	1	1	1	1	1	1	1	1	1	1	1	1	1	1	1	1	1	1	1	1	1	1	1	1
K02517	2	2	2	1	2	2	2	2	2	2	2	2	2	2	2	2	2	2	2	2	2	2	2	2	2	2
K02518	1	1	1	1	1	1	1	1	1	1	1	1	1	1	1	1	1	1	1	1	1	1	1	1	1	1
K02519	1	1	1	1	1	1	1	1	1	1	1	1	1	1	1	1	1	1	1	1	1	1	1	1	1	1
K02520	1	1	1	1	1	1	1	1	1	1	1	1	1	1	1	1	1	1	1	1	1	1	1	1	1	1
K02523	1	1	1	1	1	1	1	1	1	1	1	1	1	1	1	1	1	1	1	1	1	1	1	1	1	1
K02527	1	1	1	1	1	1	1	1	1	1	1	1	1	1	1	1	1	1	1	1	1	1	1	1	1	1
K02528	1	1	1	1	1	1	1	1	1	1	1	1	1	1	1	1	1	1	1	1	1	1	1	1	1	1
K02535	1	1	1	1	1	1	1	1	1	1	1	1	1	1	1	1	1	1	1	1	1	1	1	1	1	1
K02536	1	1	1	1	1	1	1	1	1	1	1	1	1	1	1	1	1	1	1	1	1	1	1	1	1	1
K02548	0	0	0	1	0	0	1	1	0	0	0	0	0	0	0	0	0	0	0	0	0	0	0	0	0	0
K02553	1	1	1	1	1	1	1	1	1	1	1	1	1	1	1	1	1	1	1	1	1	1	1	0	1	1
K02554	0	0	0	0	1	0	0	0	0	0	0	0	0	0	0	1	0	0	0	0	0	0	0	0	0	0
K02557	0	1	0	0	0	0	0	0	0	0	0	0	0	0	0	0	0	0	0	0	0	0	0	0	0	0
K02558	1	1	1	1	1	1	1	1	1	1	1	1	1	1	1	1	1	1	1	1	1	1	1	0	1	1
K02563	1	1	1	1	1	1	1	1	1	1	1	1	1	1	1	1	1	1	1	1	1	1	1	1	1	1
K02571	0	0	0	1	0	0	1	0	0	0	0	0	0	0	0	0	0	0	0	0	0	0	0	0	0	0
K02575	3	2	1	3	0	0	1	2	1	1	0	1	2	2	2	2	1	2	2	1	3	0	0	0	1	1
K02600	1	1	1	1	1	1	1	1	1	1	1	1	1	1	1	1	1	1	1	1	1	1	1	1	1	1
K02601	1	1	1	1	1	1	1	1	1	1	1	1	1	1	1	1	1	1	1	1	1	1	1	1	1	1
K02609	0	0	0	1	0	0	0	0	0	0	0	0	1	0	1	0	0	0	0	0	0	0	0	0	0	0
K02610	0	0	0	1	0	0	0	0	0	0	1	0	1	0	1	0	0	0	0	0	0	0	0	0	0	0
K02611	0	0	0	1	0	0	0	0	0	0	1	0	1	0	1	0	0	0	0	0	0	0	0	0	0	0
K02612	0	0	0	1	0	0	0	0	0	0	1	0	1	0	1	0	0	0	0	0	0	0	0	0	0	0

K02613	0	0	0	1	0	0	0	0	0	0	1	0	1	0	1	0	0	0	0	0	0	0	0	0	0	0
K02614	0	0	0	1	0	0	0	0	0	0	0	0	0	0	1	0	0	0	0	0	0	0	0	0	0	0
K02615	0	0	0	1	1	0	0	0	0	0	0	0	0	0	0	0	0	0	0	0	0	0	0	0	0	0
K02616	0	0	0	0	0	0	0	0	1	0	2	0	1	0	0	0	1	0	0	1	0	1	1	1	1	0
K02617	0	0	0	0	0	0	0	0	0	0	1	0	1	0	0	0	0	0	0	0	0	0	0	0	0	0
K02618	0	0	0	0	0	0	0	0	0	0	0	0	1	0	0	0	0	0	0	0	0	0	0	0	0	0
K02619	1	1	1	1	1	1	1	1	1	1	1	1	1	1	1	1	1	1	1	1	1	1	1	1	1	1
K02621	1	1	1	1	1	1	1	1	1	1	1	1	1	1	1	1	1	1	1	1	1	1	1	1	1	1
K02622	1	1	1	1	1	1	1	1	1	1	1	1	1	1	1	1	1	1	1	1	1	1	1	1	1	1
K02624	0	0	0	1	1	0	0	0	0	0	0	0	1	0	1	0	0	1	1	0	0	0	0	0	0	0
K02650	2	2	2	1	2	1	1	1	2	1	2	1	2	2	2	2	2	2	2	1	1	2	2	2	2	2
K02652	1	1	1	1	1	1	1	1	1	1	1	1	1	1	1	1	1	1	1	1	1	1	1	1	1	1
K02653	1	1	1	1	1	1	1	0	1	1	0	1	1	1	1	1	1	1	1	1	1	1	1	1	1	1
K02654	1	1	1	2	1	1	1	1	1	1	1	1	1	1	1	1	1	1	1	1	1	1	1	1	1	1
K02655	2	2	2	1	2	2	1	1	2	2	2	2	2	2	2	2	2	2	2	2	1	2	2	2	2	2
K02656	1	1	1	1	1	1	1	1	1	1	1	0	1	0	1	1	1	1	1	1	1	1	1	1	1	1
K02657	1	1	1	1	1	1	0	0	1	1	1	1	1	1	1	1	1	1	1	1	1	1	1	1	1	1
K02658	1	1	1	1	1	1	0	0	1	1	1	1	1	1	1	1	1	1	1	1	1	1	1	1	1	1
K02659	1	1	1	1	1	1	0	0	1	1	1	1	1	1	1	1	1	1	1	1	1	1	1	1	1	1
K02660	1	1	1	1	1	1	0	0	1	1	1	1	1	1	1	1	1	1	1	1	1	1	1	1	1	1
K02661	1	1	0	0	1	0	0	0	1	1	1	1	1	1	1	1	1	0	0	1	0	0	0	1	1	1
K02662	1	1	1	0	1	1	1	1	1	1	1	1	1	1	1	1	1	1	1	1	1	1	1	1	1	1
K02663	1	1	1	1	1	1	1	0	1	1	1	1	1	1	1	1	1	1	1	1	1	1	1	1	1	1
K02664	1	1	1	0	1	1	0	1	1	1	1	1	1	1	1	1	1	1	1	1	0	1	1	1	1	1
K02665	1	1	1	1	1	1	0	0	1	1	1	1	1	1	1	1	1	1	1	1	1	1	1	1	1	1
K02666	1	1	1	1	1	1	1	1	1	1	0	1	1	1	1	1	1	1	1	1	1	1	1	1	1	1
K02667	1	0	1	0	1	1	0	0	1	1	1	1	1	1	1	1	1	1	1	1	0	1	1	1	1	1
K02668	1	0	1	0	1	1	0	0	1	1	1	1	1	1	1	1	1	1	1	1	0	1	1	1	1	1
K02669	1	1	1	1	1	1	1	1	1	1	1	1	1	1	1	1	1	1	1	1	1	1	1	1	1	1
K02670	1	1	1	0	1	1	0	0	1	1	1	1	1	1	1	1	1	1	1	1	0	1	1	1	1	1
K02671	1	1	1	1	1	1	1	1	1	1	1	0	1	1	1	1	1	1	1	1	1	1	1	1	1	1
K02672	1	1	1	1	1	1	1	0	1	1	1	1	0	1	0	1	1	1	1	1	1	1	1	1	1	1
K02674	0	1	1	0	1	1	0	0	1	1	0	0	0	1	1	1	0	1	1	1	0	1	1	1	1	1
K02676	1	1	1	0	1	1	0	0	1	1	1	1	1	1	1	1	1	1	1	1	0	1	1	1	1	1
K02687	1	1	1	1	1	1	1	1	1	1	1	1	1	1	1	1	1	1	1	1	1	1	1	1	1	1
K02805	0	0	0	0	0	0	0	0	0	0	0	0	0	0	1	0	0	0	0	0	0	0	0	0	0	0
K02834	1	1	1	1	1	1	1	1	1	1	1	1	1	1	1	1	1	1	1	1	1	1	1	1	1	1
K02835	1	1	1	1	1	1	1	1	1	1	1	1	1	1	1	1	1	1	1	1	1	1	1	1	1	1
K02836	1	1	1	1	1	1	1	1	1	1	1	1	1	1	1	1	1	1	1	1	1	1	1	1	1	1
K02837	1	1	1	1	1	1	1	1	1	1	1	1	1	1	1	1	1	1	1	1	1	1	1	1	1	1
K02838	1	1	1	1	1	1	1	1	1	1	1	1	1	1	1	1	1	1	1	1	1	1	1	1	1	1
K02860	1	1	1	1	1	1	1	1	1	1	1	1	1	1	1	1	1	1	1	1	1	1	1	1	1	1
K02863	1	1	1	1	1	1	1	1	1	1	1	1	1	1	1	1	1	1	1	1	1	1	1	1	1	1
K02864	1	1	1	1	1	1	1	1	1	1	1	1	1	1	1	1	1	1	1	1	1	1	1	1	1	1
K02867	1	1	1	1	1	1	1	1	1	1	1	1	1	1	1	1	1	1	1	1	1	1	1	1	1	1
K02871	1	1	1	1	1	1	1	1	1	1	1	1	1	1	1	1	1	1	1	1	1	1	1	1	1	1
K02874	1	1	1	1	1	1	1	1	1	1	1	1	1	1	1	1	1	1	1	1	1	1	1	1	1	1
K02876	1	1	1	1	1	1	1	1	1	1	1	1	1	1	1	1	1	1	1	1	1	1	1	1	1	1
K02878	1	1	1	1	1	1	1	1	1	1	1	1	1	1	1	1	1	1	1	1	1	1	1	1	1	1
K02879	1	1	1	1	1	1	1	1	1	1	1	1	1	1	1	1	1	1	1	1	1	1	1	1	1	1
K02881	1	1	1	1	1	1	1	1	1	1	1	0	1	1	1	1	1	1	1	1	1	1	1	1	1	1
K02884	1	1	1	1	1	1	1	1	1	1	1	1	1	1	1	1	1	1	1	1	1	1	1	1	1	1
K02886	1	1	1	1	1	1	1	1	1	1	1	1	1	1	1	1	1	1	1	1	1	1	1	1	1	1
K02887	1	1	1	1	1	1	1	1	1	1	1	1	1	1	1	1	1	1	1	1	1	1	1	1	1	1
K02888	1	1	1	1	1	1	1	1	1	1	1	1	1	1	1	1	1	1	1	1	1	1	1	1	1	1
K02890	1	1	1	1	1	1	1	1	1	1	1	1	1	1	1	1	1	1	1	1	1	1	1	1	1	1
K02892	1	1	1	1	1	1	1	1	1	1	0	0	1	1	1	1	1	1	1	1	1	1	1	1	1	1
K02895	1	1	1	1	1	1	1	1	1	1	1	0	1	1	1	1	1	1	1	1	1	1	1	1	1	1
K02897	1	1	1	1	1	1	1	1	1	1	1	1	1	1	1	1	1	1	1	1	1	1	1	1	1	1
K02899	1	1	1	1	1	1	1	1	1	1	1	1	0	0	1	1	1	1	1	1	1	1	1	1	1	1
K02902	1	1	1	1	1	1	1	1	1	1	0	1	1	1	1	1	1	1	1	1	1	1	1	1	1	1
K02904	1	1	1	1	1	1	1	1	1	1	1	1	1	1	1	1	1	1	1	1	1	1	1	1	1	1
K02906	1	1	1	1	1	1	1	1	1	1	1	1	1	1	1	1	1	1	1	1	1	1	1	1	1	1
K02907	1	1	1	1	1	1	1	1	1	1	1	1	1	1	1	1	1	1	1	1	1	1	1	1	1	1
K02909	1	1	1	1	1	1	1	1	1	1	1	1	1	1	1	1	1	1	1	1	1	0	0	1	1	1
K02911	1	1	1	1	1	1	1	1	1	1	1	1	0	1	1	1	1	1	1	1	1	1	1	1	1	1
K02913	1	1	1	1	1	1	1	1	1	1	1	1	1	1	1	1	1	1	1	1	1	1	1	1	1	1
K02914	1	1	1	1	1	1	1	1	1	1	1	0	1	1	1	1	1	1	1	1	1	1	1	1	1	0

K02916	1	1	1	1	1	1	1	1	1	1	1	1	1	1	1	1	1	1	1	1	1	1	1	1	1	1
K02919	1	1	1	2	1	0	0	1	0	1	1	1	0	1	1	1	1	1	1	1	1	0	0	0	0	1
K02926	1	1	1	1	1	1	1	1	1	1	1	1	1	1	1	1	1	1	1	1	1	1	1	1	1	1
K02931	1	1	1	1	1	1	1	1	1	1	1	1	1	1	1	1	1	1	1	1	1	1	1	1	1	1
K02933	1	1	1	1	1	1	1	1	1	1	1	1	1	1	1	1	1	1	1	1	1	1	1	1	1	1
K02935	1	1	1	1	1	1	1	0	1	1	1	1	1	1	1	1	1	1	1	1	1	1	1	1	1	1
K02939	1	1	1	1	1	1	1	1	1	1	1	1	1	1	1	1	1	1	1	1	1	1	1	1	1	1
K02945	1	1	1	1	1	1	1	1	1	1	1	1	1	1	1	1	1	1	1	1	1	1	1	1	1	1
K02946	1	1	1	1	1	1	1	1	1	1	1	1	1	1	1	1	1	1	1	1	1	1	1	1	1	1
K02948	1	1	1	1	1	1	1	1	1	1	1	1	0	1	1	1	1	1	1	1	1	1	1	1	1	1
K02950	1	1	1	1	1	1	1	1	1	1	1	1	1	1	1	1	1	1	1	1	1	1	1	1	1	1
K02952	1	1	1	1	1	1	1	1	1	1	1	1	1	1	1	1	1	1	1	1	1	1	1	1	1	1
K02954	1	1	1	1	1	1	1	1	1	1	1	1	1	1	1	1	1	1	1	1	1	1	1	1	1	1
K02956	1	1	1	1	1	1	1	1	1	1	0	1	1	1	1	1	1	1	1	1	1	1	1	1	1	1
K02959	1	1	1	1	1	1	1	1	1	1	1	1	1	1	1	1	1	1	1	1	1	1	1	1	1	1
K02961	1	1	1	1	1	1	1	1	1	1	1	1	1	1	1	1	1	1	1	1	1	1	1	1	1	1
K02963	1	1	1	1	1	1	1	1	1	1	1	1	1	1	1	1	1	1	1	1	1	1	1	1	1	1
K02965	1	1	1	1	1	1	1	1	1	1	1	1	1	1	1	1	1	1	1	1	1	1	1	1	1	1
K02967	1	1	1	1	1	1	1	1	1	1	1	1	1	0	1	1	1	1	1	1	1	1	1	1	1	1
K02968	1	1	1	1	1	1	1	1	1	1	1	1	1	1	1	1	1	1	1	1	1	1	1	1	1	1
K02970	1	1	1	1	1	1	1	1	1	1	1	1	1	1	1	1	1	1	1	1	1	1	1	1	1	1
K02982	1	1	1	1	1	1	1	1	1	1	1	1	1	1	1	1	1	1	1	1	1	1	1	1	1	1
K02986	1	1	1	1	1	1	1	1	1	1	1	1	1	1	1	1	1	1	1	1	1	1	1	1	1	1
K02988	1	1	1	1	1	1	1	1	1	1	1	1	1	1	1	1	1	1	1	1	1	1	1	1	1	1
K02990	1	1	1	1	1	1	1	1	1	1	1	1	1	1	1	1	1	1	1	1	1	1	1	1	1	1
K02992	1	1	1	1	1	1	1	1	1	1	1	1	1	1	1	1	1	1	1	1	1	1	1	1	1	1
K02994	1	1	1	1	1	1	1	1	1	1	1	1	1	0	1	1	1	1	1	1	1	1	1	1	1	1
K02996	1	1	1	1	1	1	1	1	1	1	1	1	1	1	1	1	1	1	1	1	1	1	1	1	1	1
K03040	1	1	1	1	1	1	1	1	1	1	1	1	1	1	1	1	1	1	1	1	1	1	1	1	1	1
K03043	1	1	1	1	1	1	1	1	1	1	1	1	1	1	1	1	1	1	1	1	1	1	1	1	1	1
K03046	1	1	1	1	1	1	1	1	1	1	1	1	1	1	1	1	1	1	1	1	1	1	1	1	1	1
K03060	1	1	1	1	1	1	1	1	1	1	1	1	1	1	1	1	1	1	1	1	1	1	1	1	1	1
K03070	1	1	1	1	1	1	1	1	1	1	1	1	1	1	1	1	1	1	1	1	1	1	1	1	1	1
K03071	1	1	1	1	1	1	1	1	1	1	1	1	1	1	1	2	1	1	1	1	1	2	2	1	1	1
K03072	1	1	1	1	1	1	1	1	1	1	1	1	1	1	1	1	1	1	1	1	1	1	1	1	1	1
K03073	1	1	1	1	1	1	1	1	1	1	1	1	1	1	1	1	1	1	1	1	1	1	1	0	1	1
K03074	1	1	1	1	1	1	1	1	1	1	1	1	1	1	1	1	1	1	1	1	1	1	1	1	1	1
K03075	1	1	1	1	1	1	1	1	1	1	1	0	1	1	1	1	1	1	1	1	1	1	1	1	1	1
K03076	1	1	1	1	1	1	1	1	1	1	1	1	1	1	1	1	1	1	1	1	1	1	1	0	1	1
K03086	1	1	1	1	1	1	1	1	1	1	1	1	1	1	1	1	1	1	1	1	1	1	1	1	1	1
K03088	0	0	0	1	0	0	0	0	0	0	0	0	0	0	0	0	0	0	0	0	0	0	0	0	0	0
K03089	1	1	1	1	1	1	1	1	1	1	1	1	1	1	1	1	1	1	1	1	1	1	1	1	1	1
K03092	1	1	1	1	1	1	1	1	1	1	1	1	1	1	1	1	1	1	1	1	1	1	1	1	1	1
K03098	1	0	1	0	1	1	1	1	1	1	0	1	1	1	0	1	0	1	1	1	1	1	1	1	1	1
K03100	1	1	1	1	1	1	1	1	1	1	1	1	1	1	1	1	1	1	1	1	1	1	1	0	1	0
K03101	1	1	2	1	2	3	1	2	2	2	2	1	2	2	1	2	3	2	2	1	1	2	1	2	2	2
K03106	1	1	1	1	1	1	1	1	1	1	1	1	1	1	1	1	1	1	1	1	1	1	1	1	1	1
K03110	1	1	1	1	1	1	1	1	1	1	1	1	1	1	1	1	1	1	1	1	1	1	1	1	1	1
K03111	1	1	1	1	1	1	1	1	1	1	1	1	1	1	1	1	1	1	1	1	1	1	1	1	1	2
K03116	1	2	2	1	1	1	1	1	2	2	2	1	2	1	2	2	2	2	1	1	2	2	2	2	2	2
K03117	1	1	1	1	1	1	1	1	2	1	2	1	2	0	1	2	2	2	1	1	2	2	2	2	2	2
K03118	1	1	1	1	1	1	1	1	2	1	2	1	2	1	1	2	2	2	1	1	2	2	2	2	2	2
K03119	0	0	0	1	0	0	0	0	0	0	0	0	0	0	1	0	0	1	1	0	0	0	0	0	0	0
K03147	1	1	1	1	1	1	1	1	1	1	1	0	1	1	1	1	1	1	1	1	1	1	1	1	1	1
K03149	1	1	1	1	1	1	1	1	1	1	1	1	1	1	1	1	1	1	1	1	1	1	1	1	1	1
K03154	1	1	1	1	1	1	1	1	1	1	1	1	1	1	1	1	1	1	1	1	1	1	1	1	1	1
K03168	2	1	2	1	2	2	2	2	2	2	1	2	2	2	2	2	2	2	2	2	2	2	2	2	2	2
K03177	1	1	1	1	1	1	1	1	1	1	1	1	1	1	1	1	1	1	1	1	1	1	1	1	1	1
K03179	1	1	1	1	1	1	1	1	1	1	1	1	1	1	1	1	1	1	1	1	1	1	1	1	1	1
K03181	1	1	1	1	1	1	1	1	1	1	1	1	1	1	1	1	1	1	1	1	1	1	1	1	1	1
K03183	1	1	1	1	1	1	1	1	1	1	1	1	1	1	1	1	1	1	1	1	1	1	1	1	1	1
K03185	1	1	1	1	1	1	1	1	1	1	1	1	1	1	1	1	1	1	1	1	1	1	1	1	1	1
K03187	1	0	2	1	1	1	1	2	1	2	1	1	1	1	0	1	1	1	1	1	0	1	1	0	1	0
K03188	1	0	2	1	1	1	1	2	1	2	1	1	1	1	0	1	1	1	1	1	0	1	1	1	1	0
K03189	1	0	2	1	1	1	1	2	1	2	0	1	1	1	0	1	1	1	1	1	0	1	1	1	1	0
K03190	1	0	2	1	1	1	1	2	1	2	1	1	1	1	0	1	1	1	1	1	0	1	1	1	1	0
K03192	1	0	1	0	1	1	0	2	1	1	1	0	1	1	0	1	1	1	1	1	0	1	1	1	1	0
K03210	1	1	1	1	1	1	1	1	1	1	1	1	1	1	1	1	1	1	1	1	1	1	1	1	1	1

K03215	1	1	1	1	1	1	1	1	1	1	1	1	1	1	1	1	1	1	1	1	1	1	1	1	1	1
K03216	1	1	1	1	1	1	1	1	1	1	1	1	1	1	1	1	1	1	1	1	1	1	1	1	1	1
K03217	1	1	1	1	1	1	1	1	1	1	1	1	1	1	1	1	1	1	1	1	1	1	1	1	1	1
K03218	1	1	1	1	1	1	1	1	1	1	1	1	1	1	1	1	1	1	1	1	1	1	1	1	1	1
K03269	1	1	1	1	1	1	1	1	1	1	1	1	1	1	1	1	1	1	1	1	1	1	1	1	1	1
K03270	1	1	1	1	1	1	1	1	1	1	1	1	1	1	1	1	1	1	1	1	1	1	1	1	1	1
K03282	0	1	1	1	1	1	1	1	1	1	1	1	1	1	0	1	1	0	0	1	1	0	0	1	1	2
K03284	1	1	1	1	1	2	1	1	2	1	1	1	1	1	1	1	1	2	2	1	1	1	1	2	2	1
K03286	2	1	4	1	2	2	1	1	4	3	1	0	4	3	2	2	1	3	4	4	1	1	1	4	4	3
K03294	0	0	0	0	0	0	0	0	0	0	0	0	0	0	0	1	0	0	0	0	0	0	0	0	0	0
K03297	1	1	1	1	1	1	1	1	1	1	0	1	1	2	1	1	1	1	1	2	1	1	1	1	1	1
K03298	0	0	0	0	0	0	0	0	0	0	1	1	0	0	0	0	1	0	0	0	0	0	0	0	0	0
K03299	1	0	1	0	1	1	0	0	0	1	1	1	1	1	0	1	1	0	0	0	1	0	0	0	0	1
K03300	0	0	0	0	0	1	0	0	0	0	0	0	0	0	0	0	0	0	0	0	0	0	0	0	0	1
K03303	0	1	1	1	2	2	1	0	1	1	1	2	1	1	0	1	0	2	1	0	1	1	1	1	1	1
K03305	1	0	1	1	1	1	1	0	1	1	1	1	1	1	0	1	1	1	1	1	1	1	1	1	1	1
K03306	1	1	1	1	1	1	1	1	1	1	1	1	1	1	1	1	1	1	1	1	1	1	1	1	1	1
K03307	1	1	1	2	1	1	1	1	1	1	1	1	1	1	1	1	1	2	2	1	1	1	1	1	1	1
K03308	1	1	1	1	1	1	1	1	1	1	1	1	1	1	1	1	1	1	1	1	1	1	1	1	1	1
K03310	3	1	3	3	2	2	2	2	3	3	5	3	2	3	1	3	5	4	4	4	2	3	3	3	3	3
K03311	0	0	0	1	0	0	1	1	0	0	0	0	0	0	0	0	0	0	0	0	1	0	0	0	0	0
K03312	1	0	1	1	1	0	1	1	1	1	1	1	1	1	0	1	1	1	1	1	1	1	1	1	1	1
K03313	1	1	1	1	1	1	1	1	1	1	1	1	1	1	1	1	1	1	1	1	1	1	1	1	1	1
K03315	1	0	2	1	3	1	2	3	2	1	2	1	1	2	1	2	2	2	2	2	2	1	1	2	2	1
K03316	1	1	1	1	1	1	0	1	1	1	1	1	1	1	1	1	1	1	1	1	1	1	1	0	1	1
K03319	1	0	0	1	1	0	1	1	1	0	0	0	1	1	1	1	0	1	1	0	1	1	1	1	1	1
K03320	1	1	1	1	1	2	1	2	1	1	2	2	1	1	1	1	2	1	1	2	1	1	1	1	1	1
K03321	3	2	2	2	2	2	2	2	2	2	3	3	2	2	3	2	3	2	2	2	2	2	2	2	2	2
K03324	0	0	0	1	0	0	1	1	0	0	0	0	0	0	0	0	0	0	0	0	1	0	0	0	0	0
K03327	2	2	2	2	2	2	2	2	2	2	2	2	2	2	2	3	2	2	2	2	2	2	2	2	2	2
K03328	0	0	0	0	0	0	0	0	0	0	0	0	0	0	0	0	0	2	1	0	0	0	0	0	0	1
K03333	0	0	0	1	0	1	1	1	0	0	0	0	1	1	0	1	0	1	1	0	1	0	0	0	0	0
K03340	0	0	0	1	0	0	1	0	0	0	0	0	0	0	0	0	0	0	0	0	1	0	0	0	0	0
K03381	1	0	1	1	1	0	0	0	0	1	0	0	0	0	0	1	0	1	1	0	0	1	1	0	0	1
K03386	1	1	1	1	1	1	1	1	1	1	1	1	1	1	1	1	1	1	1	1	1	1	1	1	1	1
K03387	1	1	1	1	1	1	1	1	1	1	1	1	1	1	1	1	1	1	1	1	1	1	1	1	1	1
K03409	2	0	2	0	2	2	0	0	0	2	2	2	2	2	2	2	2	2	2	2	0	0	0	0	0	2
K03412	0	0	1	0	0	0	0	0	0	0	0	0	0	0	0	0	0	0	0	0	0	0	0	0	0	0
K03413	1	0	1	0	1	1	0	0	0	1	1	1	1	1	1	1	1	1	1	1	0	0	0	0	0	1
K03417	1	1	1	1	1	1	1	1	1	1	1	1	1	1	1	1	1	1	1	1	1	1	1	1	1	1
K03424	2	2	2	2	2	2	2	2	2	1	2	1	2	2	2	2	3	2	2	4	2	2	2	2	2	2
K03426	1	1	1	0	1	1	0	0	1	1	1	1	1	1	1	1	1	1	1	3	0	1	1	1	1	1
K03427	2	2	4	3	0	3	1	2	5	1	1	1	0	2	2	2	2	1	2	2	2	2	1	5	5	2
K03431	1	1	1	1	1	1	1	1	1	1	1	1	1	1	1	1	1	1	1	1	1	1	1	1	1	1
K03437	1	1	1	1	1	1	1	1	1	1	1	1	1	1	1	1	1	1	1	1	1	1	1	1	1	1
K03438	1	1	1	1	1	1	1	1	1	1	1	1	1	1	1	1	1	1	1	1	1	1	1	1	1	1
K03439	1	1	1	1	1	1	1	1	1	1	1	1	1	1	1	1	1	1	1	1	1	1	1	1	1	1
K03442	1	1	1	1	1	1	1	1	1	1	1	1	1	1	1	1	1	1	1	1	1	1	1	1	1	1
K03449	1	1	1	1	3	1	1	1	1	2	1	1	2	2	1	3	1	1	1	2	1	1	1	1	1	2
K03451	0	0	0	1	0	0	2	1	0	1	0	0	0	0	1	0	0	2	2	0	1	0	0	0	0	0
K03453	2	0	2	3	1	2	5	3	2	2	5	5	2	1	2	1	5	2	2	1	2	1	1	2	2	3
K03457	0	0	2	1	0	0	1	0	0	2	0	0	0	0	0	0	0	2	2	0	0	0	0	0	0	0
K03462	1	1	1	1	1	1	1	0	1	1	1	1	1	1	1	1	1	1	1	1	1	1	1	1	1	1
K03464	1	0	1	0	1	0	0	0	0	1	0	0	0	0	0	1	0	1	1	0	0	1	1	0	0	1
K03465	0	0	0	0	0	0	0	0	0	0	0	0	0	0	0	0	0	0	0	0	0	0	0	0	0	1
K03466	1	1	1	1	1	1	2	1	1	1	1	0	1	1	1	1	1	1	1	1	1	1	1	1	1	1
K03470	1	1	1	1	1	1	1	1	1	1	1	1	1	1	1	1	1	1	1	1	1	1	1	1	1	1
K03473	1	1	1	1	1	1	1	1	1	1	1	1	1	1	1	1	1	1	1	1	1	1	1	1	1	1
K03474	1	1	1	1	2	1	1	1	1	1	1	1	1	1	2	1	1	1	1	1	1	1	1	1	1	1
K03495	1	1	1	1	1	1	1	1	1	1	1	1	1	1	1	1	1	1	1	1	1	1	1	1	1	1
K03496	1	1	2	2	2	2	2	2	2	1	2	1	2	2	1	2	2	2	1	1	2	2	1	2	2	2
K03497	1	1	1	1	1	1	1	1	1	1	1	1	1	1	1	1	1	1	1	1	1	1	1	1	1	1
K03498	0	0	0	1	0	0	1	1	0	0	0	0	0	0	0	0	0	0	0	0	1	0	0	0	0	0
K03499	1	1	1	1	1	1	1	1	1	1	1	1	1	1	1	1	1	1	1	1	1	0	0	1	1	1
K03500	1	1	1	1	1	1	1	1	1	1	1	1	1	1	1	1	1	1	1	1	1	1	1	1	1	1
K03501	1	1	1	1	1	1	1	1	1	1	1	1	1	1	1	1	1	1	1	1	1	1	1	1	1	1
K03502	0	0	0	1	0	2	1	0	0	1	0	0	0	0	0	0	0	0	0	0	0	2	2	0	0	0
K03503	1	0	0	2	0	2	1	0	0	1	0	0	0	0	1	0	0	0	0	0	0	2	2	0	0	0

K03517	1	1	1	1	1	1	1	1	1	1	1	1	1	1	1	1	1	1	1	1	1	1	1	1	1	1
K03521	1	1	1	1	1	1	1	1	1	1	1	1	1	1	1	1	1	1	1	1	1	1	1	1	1	1
K03522	1	1	1	1	1	1	1	1	1	1	1	1	1	1	1	1	1	1	1	1	1	1	1	1	1	1
K03524	1	1	1	1	1	1	1	1	1	1	1	1	1	1	1	1	1	1	1	1	1	1	1	1	1	1
K03525	1	1	1	1	1	1	1	1	1	1	1	1	1	1	1	1	1	1	1	1	1	1	1	1	1	1
K03526	1	1	1	1	1	1	1	1	1	1	1	1	1	1	1	1	1	1	1	1	1	1	1	1	1	1
K03527	1	1	1	1	1	1	1	1	1	1	1	1	1	1	1	1	1	1	1	1	1	1	1	1	1	1
K03528	1	1	1	1	1	1	1	1	1	1	1	1	1	1	1	1	1	1	1	1	1	1	1	1	1	1
K03529	1	1	1	1	1	1	1	1	1	1	1	1	1	1	1	1	1	1	1	1	1	2	2	1	1	1
K03530	2	1	1	1	1	1	1	1	1	1	1	1	1	1	1	1	1	1	1	1	1	1	1	1	1	1
K03531	1	1	1	1	1	1	1	1	1	1	1	1	1	1	1	1	1	1	1	1	1	1	1	1	1	1
K03532	0	0	0	1	0	0	1	0	0	0	0	0	0	0	0	0	0	0	0	0	1	0	0	0	0	0
K03533	0	0	0	1	0	0	1	0	0	0	0	0	0	0	0	0	0	0	0	0	1	0	0	0	0	0
K03534	0	0	0	0	0	0	0	0	0	0	1	0	0	0	0	0	0	0	0	0	0	0	0	0	0	0
K03536	1	1	1	1	1	1	1	1	1	1	1	1	1	1	1	1	1	1	1	1	1	1	1	1	1	1
K03544	1	1	1	1	1	1	1	1	1	1	1	1	1	1	1	1	1	1	1	1	1	1	1	1	1	1
K03545	1	1	1	1	1	1	1	1	1	1	1	0	1	1	1	1	1	1	1	1	1	1	1	1	1	1
K03546	1	1	1	1	1	1	1	1	1	1	1	0	1	1	1	1	1	1	1	1	1	1	1	1	1	1
K03547	1	1	1	1	1	1	1	1	1	1	0	1	1	1	1	1	1	1	1	1	1	1	1	1	1	1
K03550	1	1	1	1	1	1	1	1	1	1	1	1	1	1	1	1	1	1	1	1	1	1	1	1	1	1
K03551	1	1	1	1	1	1	1	1	1	1	1	1	1	1	1	1	1	1	1	1	1	1	1	1	1	1
K03553	1	1	1	1	1	1	1	1	1	1	1	1	1	1	1	1	1	1	1	1	1	1	1	1	1	1
K03555	1	1	1	1	1	1	1	1	1	1	1	1	1	1	1	1	1	1	1	1	1	1	1	1	1	1
K03556	0	1	0	0	0	0	0	0	0	0	0	0	0	0	0	0	0	0	0	0	0	0	0	0	0	0
K03557	1	1	1	1	1	1	1	1	1	1	1	1	1	1	1	1	1	1	1	1	1	1	1	1	1	1
K03558	1	1	1	1	1	1	1	1	1	1	1	1	1	1	1	1	1	1	1	1	1	1	1	1	1	1
K03559	2	2	3	1	2	2	2	1	2	3	2	2	2	2	3	3	2	2	2	3	1	2	2	2	2	2
K03560	1	1	1	1	1	1	1	1	1	1	1	1	1	1	1	1	1	1	1	1	1	1	1	1	1	1
K03561	2	2	3	1	2	2	2	1	2	3	2	2	2	2	3	3	2	2	2	3	1	2	2	2	2	2
K03562	1	1	1	1	1	1	1	1	1	1	1	1	1	1	1	1	1	1	1	1	1	1	1	1	1	1
K03563	1	1	1	1	1	1	1	1	1	1	1	1	1	1	1	1	1	1	1	1	1	1	1	1	1	1
K03564	1	1	1	1	1	1	1	1	1	1	1	1	1	1	1	1	1	1	1	1	1	1	1	1	1	1
K03565	1	1	1	1	1	1	1	1	1	1	1	1	1	1	1	1	1	1	1	1	1	1	1	1	1	1
K03566	1	0	0	1	0	0	0	0	0	0	2	0	0	0	1	0	1	0	0	0	0	0	0	0	0	1
K03569	1	1	1	1	1	1	1	1	1	1	1	1	1	1	1	1	1	1	1	1	1	1	1	1	1	1
K03570	1	1	1	1	1	1	1	1	1	1	1	1	1	1	1	1	1	1	1	1	1	1	1	1	1	1
K03571	1	1	1	1	1	1	1	1	1	1	1	1	1	1	1	1	1	1	1	1	1	1	1	1	1	1
K03572	1	1	1	1	1	1	1	1	1	1	1	1	1	1	1	1	1	1	1	1	1	1	1	1	1	1
K03574	1	1	1	1	1	1	1	2	1	1	1	1	1	1	1	1	1	2	1	1	2	1	1	1	1	1
K03575	1	1	1	1	1	1	1	1	1	1	0	0	1	1	1	1	1	1	1	1	1	1	1	1	1	1
K03576	1	1	1	1	1	1	1	1	1	1	1	1	1	1	1	1	1	1	1	1	1	1	1	1	1	1
K03578	1	1	1	1	1	1	1	1	1	0	1	1	1	1	1	1	1	1	1	1	1	1	1	1	1	1
K03580	1	1	1	1	1	1	1	0	1	1	0	1	0	1	1	1	1	1	1	1	1	1	1	1	1	1
K03581	1	1	1	1	1	1	1	1	1	1	1	1	1	1	1	1	1	1	1	1	1	1	1	1	1	1
K03582	1	1	1	1	1	1	1	1	1	1	1	0	1	1	1	1	1	1	1	1	1	1	1	1	1	1
K03583	1	1	1	1	1	1	1	1	1	1	1	1	1	1	1	1	1	1	1	1	1	1	1	1	1	1
K03584	1	1	1	1	1	1	0	1	1	1	1	1	1	1	1	1	1	1	1	1	1	1	1	1	1	1
K03585	1	1	1	1	1	1	1	1	1	1	0	1	1	1	1	1	1	1	1	1	1	1	1	1	1	1
K03586	1	1	1	1	1	1	1	1	1	1	1	1	1	1	1	1	1	1	1	1	1	1	1	1	1	1
K03587	1	1	1	1	1	1	1	1	1	1	1	1	1	1	1	1	1	1	1	1	1	1	1	1	1	1
K03588	1	1	1	1	1	1	1	1	1	1	1	1	1	1	1	1	1	1	1	1	1	1	1	1	1	1
K03589	1	1	1	1	1	1	1	1	1	1	1	1	1	1	1	1	1	1	1	1	1	1	1	1	1	1
K03590	1	1	1	1	1	1	1	1	1	1	1	1	1	1	1	1	1	1	1	1	1	1	1	1	1	1
K03593	1	1	1	1	1	1	1	1	1	1	1	1	1	1	1	1	1	1	1	1	1	1	1	1	1	1
K03594	2	2	2	2	2	2	2	2	2	2	2	2	2	1	2	2	2	2	2	2	2	2	2	2	2	2
K03595	1	1	1	1	1	1	1	1	1	1	1	0	1	1	1	1	1	1	1	1	1	1	1	1	1	1
K03596	1	1	1	1	1	1	1	1	1	1	1	0	1	1	1	1	1	1	1	1	1	1	1	1	1	1
K03599	1	1	1	1	1	1	1	1	1	1	1	1	1	1	1	1	1	1	1	1	1	1	1	1	1	1
K03600	1	1	1	1	1	1	1	1	1	1	1	1	1	1	1	1	1	1	1	1	1	1	1	1	1	1
K03601	1	1	1	1	1	1	1	1	1	1	1	1	1	1	1	1	1	1	1	1	1	1	1	1	1	1
K03608	1	1	1	1	1	1	1	1	1	1	1	1	1	1	1	1	1	1	1	1	1	1	1	1	1	1
K03609	1	1	1	1	1	1	1	1	1	1	1	1	1	1	1	1	1	1	1	1	1	1	1	1	1	1
K03610	1	1	1	1	1	1	1	1	1	1	1	1	1	1	1	1	1	1	1	1	1	1	1	1	1	1
K03611	1	1	1	1	1	1	1	1	1	1	1	1	1	1	1	1	1	1	1	1	1	1	1	1	1	1
K03616	1	1	1	1	1	1	1	1	1	1	1	1	1	1	1	1	1	1	1	3	1	1	1	0	1	1
K03624	1	1	1	1	1	1	1	1	1	1	1	1	1	1	1	1	1	1	1	1	1	1	1	1	1	1
K03625	1	1	1	1	1	1	1	1	1	1	1	1	1	1	1	1	1	1	1	1	1	1	1	1	1	1
K03628	1	1	1	1	1	1	1	1	1	1	1	1	1	1	1	1	1	1	1	1	1	1	1	1	1	1

K03629	1	1	1	1	1	1	1	1	1	1	1	1	1	1	1	1	1	1	1	1	1	1	1	1	1	1
K03630	1	1	1	1	1	1	1	1	1	1	1	1	1	1	1	1	1	1	1	1	1	1	1	1	1	1
K03631	1	1	1	1	1	1	1	1	1	1	1	1	1	1	1	1	1	1	1	1	1	1	1	0	1	1
K03634	1	1	1	1	1	1	1	1	1	1	1	1	1	1	1	1	1	1	1	1	1	1	1	1	1	1
K03635	1	1	1	1	1	1	1	1	1	1	1	1	0	1	1	1	1	2	2	1	1	1	1	1	1	1
K03636	2	1	1	1	1	1	1	1	1	1	1	1	1	1	1	1	1	2	2	1	1	1	1	1	1	1
K03637	1	1	1	1	1	1	1	1	1	1	1	1	1	1	1	1	1	2	2	1	1	1	1	1	1	1
K03638	2	1	1	1	1	1	1	1	1	1	1	1	1	1	1	1	1	2	2	1	1	1	1	1	1	0
K03639	2	1	1	1	1	1	1	1	1	1	1	1	1	1	1	1	1	2	2	1	1	1	1	1	1	1
K03640	1	1	1	1	1	1	1	1	1	1	1	1	1	1	1	1	1	1	1	1	1	1	1	0	1	1
K03641	2	1	1	1	2	1	1	1	2	1	1	1	0	1	1	1	1	1	1	1	1	1	1	2	2	2
K03642	2	2	2	2	2	2	2	2	2	2	2	2	2	2	2	2	2	2	2	2	2	2	2	2	2	2
K03643	1	1	1	1	1	1	1	1	1	1	1	1	1	1	1	1	1	1	1	1	1	1	1	1	1	1
K03644	1	1	1	1	1	1	1	1	1	1	1	1	1	1	1	1	1	1	1	1	1	1	1	1	1	1
K03648	1	1	1	1	1	1	1	1	1	1	1	1	1	1	1	1	1	1	1	1	1	1	1	1	1	1
K03650	1	1	1	1	1	1	1	1	1	1	1	1	1	1	1	1	1	1	1	1	1	1	1	1	1	1
K03651	1	1	1	1	1	1	1	1	1	1	1	1	1	1	1	1	1	1	1	1	1	1	1	1	1	1
K03652	1	0	1	0	1	1	0	0	1	1	1	1	1	1	1	1	1	1	1	1	0	1	1	1	1	1
K03655	1	1	1	2	1	1	1	1	3	1	1	1	1	1	2	2	1	1	1	1	1	1	1	3	3	1
K03656	1	1	1	1	1	1	1	1	1	1	1	1	1	1	1	1	1	1	1	1	1	1	1	1	1	1
K03657	0	0	1	1	1	1	1	1	1	1	0	1	1	1	1	1	1	1	1	3	1	1	1	1	1	1
K03658	0	0	1	0	0	0	0	0	0	0	0	0	0	0	0	0	0	0	0	0	0	0	0	0	0	0
K03664	1	1	1	1	1	1	1	1	1	1	1	1	1	1	2	1	1	1	1	1	1	1	1	1	1	1
K03665	1	1	1	1	1	1	1	1	1	1	1	1	1	1	1	1	1	1	1	1	1	1	1	1	1	1
K03666	1	1	1	1	1	1	1	1	1	1	1	0	1	1	1	1	1	1	1	1	1	1	1	1	1	1
K03671	1	1	1	1	1	1	1	1	1	1	1	1	1	1	1	1	1	1	1	1	1	1	1	1	1	1
K03672	1	1	1	1	1	1	1	1	1	1	1	1	1	1	1	1	1	1	1	1	1	1	1	1	1	1
K03673	1	1	1	1	1	1	1	1	1	1	1	1	1	1	1	1	1	1	1	1	1	1	1	1	1	1
K03676	1	1	1	1	1	1	1	1	1	1	1	1	1	1	1	1	1	1	1	1	1	1	1	1	1	1
K03683	1	1	1	1	1	1	1	1	1	1	1	1	1	1	1	1	1	1	1	1	1	1	1	1	1	1
K03684	1	1	1	1	1	1	1	1	1	1	1	1	1	1	1	1	1	1	1	1	1	1	1	1	1	1
K03685	1	1	1	1	1	1	1	1	1	1	1	1	1	1	1	1	1	1	1	1	1	1	1	0	1	1
K03686	1	1	1	1	1	1	1	1	1	1	1	1	1	1	1	1	1	1	1	1	1	1	1	1	1	1
K03687	1	1	1	1	1	1	1	1	1	1	1	1	1	1	1	1	1	1	1	1	1	1	1	1	1	1
K03688	1	1	1	1	1	1	1	1	1	1	1	1	1	1	1	1	1	1	1	1	1	1	1	0	1	1
K03690	1	1	1	1	1	1	1	1	1	1	1	1	1	1	1	1	1	1	1	1	1	1	1	1	1	1
K03694	1	1	1	1	1	1	1	1	1	1	1	1	1	1	1	1	1	1	1	1	1	1	1	1	1	1
K03695	1	1	1	1	1	1	1	1	1	1	1	1	1	1	1	1	1	1	1	1	1	1	1	1	1	1
K03701	2	1	2	1	2	2	1	1	2	2	2	1	0	1	2	1	2	2	2	3	1	2	2	1	2	2
K03702	1	1	1	1	1	1	1	1	1	1	1	1	1	1	1	1	1	1	1	1	1	1	1	1	1	1
K03703	1	1	1	1	1	1	1	1	1	1	0	0	0	0	1	1	1	1	1	1	1	1	1	1	1	1
K03704	3	3	3	4	3	3	6	2	3	3	3	3	3	3	3	3	3	3	3	3	4	3	3	3	3	3
K03707	0	0	0	1	0	0	1	0	0	0	0	0	1	1	0	1	0	0	0	1	0	0	0	0	0	0
K03710	1	0	1	0	1	0	0	0	0	1	0	0	0	1	0	0	1	0	0	0	1	0	0	0	0	1
K03711	1	1	1	1	1	1	1	1	1	1	1	1	1	1	1	1	1	1	1	1	1	1	1	1	1	1
K03719	2	1	2	1	2	3	1	1	2	2	2	3	2	3	3	2	3	2	2	3	1	2	2	2	2	4
K03720	1	1	1	1	1	1	1	1	1	1	1	1	1	1	1	1	1	1	1	1	1	1	1	1	1	1
K03723	1	1	1	1	1	1	1	1	1	1	1	1	1	1	1	1	1	1	1	1	1	1	1	1	1	1
K03724	0	0	0	0	0	0	0	0	0	0	0	0	0	0	1	0	0	1	0	0	0	0	0	0	0	0
K03732	1	1	1	1	1	1	1	1	1	1	1	1	1	1	1	1	1	1	1	1	1	1	1	1	1	1
K03733	1	1	1	1	1	1	1	2	1	1	1	1	1	1	1	1	1	1	1	1	1	2	2	0	1	1
K03734	1	2	1	1	2	1	2	2	2	2	1	2	1	1	2	1	1	2	1	1	2	2	2	2	2	1
K03735	0	0	0	0	0	0	0	0	0	0	0	0	0	0	0	0	0	1	1	0	0	0	0	0	0	0
K03736	0	0	0	0	0	0	0	0	0	0	0	0	0	0	0	0	0	1	1	0	0	0	0	0	0	0
K03741	0	0	0	1	0	0	1	1	0	0	0	0	0	0	0	0	0	0	0	0	1	0	0	0	0	0
K03742	0	0	0	0	0	0	1	1	0	0	0	0	0	0	0	0	0	0	0	0	0	0	0	0	0	0
K03743	0	0	1	0	0	0	0	1	0	1	0	0	0	0	0	0	0	0	1	0	0	0	0	0	0	0
K03744	2	1	2	1	2	2	1	1	2	2	2	2	2	2	1	2	2	2	2	2	1	1	1	2	2	2
K03745	1	1	1	1	1	1	1	1	1	1	1	1	1	1	1	1	1	1	1	1	1	1	1	1	1	1
K03746	1	0	1	0	1	1	0	0	1	1	1	0	1	1	1	1	1	1	1	1	0	1	1	1	1	1
K03750	2	1	1	1	1	1	1	1	1	1	1	0	1	1	1	1	1	2	2	1	1	1	1	1	1	1
K03752	1	1	1	1	1	1	1	1	1	1	1	1	1	1	1	1	1	1	1	1	1	1	1	1	1	1
K03758	1	0	0	0	1	0	0	0	0	0	0	1	0	0	0	0	0	0	0	1	1	0	0	0	0	1
K03760	0	1	1	0	1	2	1	1	2	1	1	1	1	1	1	1	1	1	1	1	1	1	1	2	2	1
K03768	1	1	1	1	1	1	1	1	1	1	1	1	1	1	1	1	1	1	1	1	1	1	1	1	1	1
K03769	2	2	1	1	1	1	1	1	1	1	1	1	2	2	1	2	1	2	2	1	2	1	1	1	1	1
K03770	1	1	1	1	1	1	1	1	1	1	1	1	1	1	1	1	1	1	1	1	1	1	1	1	1	1
K03771	1	1	1	1	1	1	1	1	1	1	0	0	1	1	1	1	1	1	1	1	1	1	1	0	1	1

K03773	1	1	1	1	1	1	1	1	1	1	1	1	1	1	1	1	1	1	1	1	1	1	1	1	1	1
K03774	1	1	1	1	1	1	1	1	1	1	1	1	1	1	1	1	1	1	1	1	1	1	1	1	1	1
K03775	1	1	1	1	1	1	1	1	1	1	1	1	1	1	1	1	1	1	1	1	1	1	1	1	1	1
K03776	1	0	0	0	1	1	0	0	1	0	1	1	1	1	1	1	1	1	1	1	0	1	1	1	1	1
K03777	0	0	0	0	2	1	0	0	1	0	1	1	2	2	1	2	2	1	1	1	1	1	1	1	1	1
K03778	0	0	0	1	0	0	0	0	0	0	0	0	0	0	0	0	0	0	0	0	0	0	0	0	0	0
K03781	1	1	2	1	1	1	2	2	2	2	1	1	2	2	1	2	1	1	1	1	2	2	2	2	2	1
K03782	1	0	0	0	1	1	0	0	1	0	1	1	1	1	1	1	1	1	1	1	0	0	0	1	1	1
K03786	1	1	1	1	1	1	1	1	1	1	1	1	1	1	1	1	1	1	1	1	1	1	1	1	1	1
K03787	1	1	1	0	1	1	0	0	1	1	1	1	1	1	0	1	1	1	1	1	0	1	1	1	1	1
K03789	1	1	1	1	1	1	1	1	1	1	1	1	1	1	1	1	1	1	1	1	1	1	1	1	1	1
K03790	0	0	0	0	0	0	1	1	0	0	0	0	0	0	0	0	0	0	0	0	1	0	0	0	0	1
K03793	0	1	1	0	0	1	0	0	1	1	1	1	0	0	1	0	1	1	1	1	0	1	1	1	1	1
K03797	2	2	2	2	2	2	2	2	2	2	1	2	2	2	2	2	2	2	2	2	2	2	2	2	2	2
K03798	1	1	1	1	1	1	1	1	1	1	1	1	1	1	1	1	1	1	1	1	1	1	1	1	1	1
K03799	1	0	0	1	1	1	1	1	1	0	1	1	1	1	0	1	1	1	1	0	1	0	0	0	1	1
K03800	1	0	1	1	1	1	1	1	1	1	1	1	1	1	1	1	1	1	1	0	1	1	1	1	1	1
K03801	1	1	1	1	1	1	1	1	1	1	1	0	1	1	1	1	1	1	1	1	1	1	1	1	1	1
K03806	1	1	1	1	1	1	1	1	1	1	0	1	1	1	1	1	1	1	1	1	1	1	1	1	1	1
K03809	2	2	2	2	2	2	2	2	2	2	2	2	2	2	2	2	2	2	2	2	2	2	2	1	2	2
K03811	2	0	2	0	2	2	1	1	2	2	2	1	2	2	1	2	2	2	2	2	1	2	2	2	2	2
K03814	1	1	1	1	1	1	1	1	1	1	1	1	1	1	1	1	1	1	1	1	1	1	1	1	1	1
K03820	1	1	2	1	2	2	1	2	2	2	2	1	2	2	1	2	3	2	2	1	1	2	1	2	2	1
K03821	0	0	0	0	0	0	1	1	0	0	0	0	0	0	0	0	0	1	1	0	0	0	0	0	0	0
K03823	1	1	1	1	1	1	1	1	1	1	1	1	1	1	1	1	1	1	1	1	1	1	1	1	1	1
K03825	0	0	0	0	0	0	0	0	0	0	0	0	0	0	0	1	0	0	0	1	0	0	0	0	0	1
K03826	0	0	0	0	0	0	0	0	0	0	0	0	0	0	0	0	0	0	0	0	0	0	0	0	0	1
K03830	0	0	0	1	0	0	0	1	0	0	0	0	0	0	0	0	0	0	0	0	0	0	0	0	0	0
K03832	2	2	2	1	2	2	1	0	2	2	2	2	2	2	2	3	2	2	2	2	1	1	1	2	2	2
K03841	1	1	1	1	1	1	1	1	1	1	1	1	1	1	1	1	1	1	1	1	1	1	1	1	1	1
K03862	0	0	0	0	1	0	0	0	0	0	0	0	0	0	0	0	0	0	0	0	0	0	0	0	0	0
K03863	0	0	0	0	1	0	0	0	0	0	0	0	0	0	0	0	0	0	0	0	0	0	0	0	0	0
K03885	0	0	0	0	0	0	0	0	1	0	0	0	0	0	0	0	0	0	0	2	0	0	0	1	1	0
K03892	2	1	1	2	1	1	1	1	1	1	1	1	1	1	1	1	2	2	2	1	1	1	1	1	1	1
K03893	1	1	1	1	1	1	1	1	1	1	1	1	1	1	1	1	1	1	1	1	1	1	1	1	1	1
K03897	1	0	0	0	1	0	0	0	0	0	0	0	0	0	0	1	0	0	0	1	0	0	0	0	0	0
K03923	0	0	0	0	0	0	0	0	0	0	0	1	0	0	0	0	0	0	0	0	1	0	0	0	0	1
K03924	1	1	1	0	1	1	0	0	1	1	1	1	1	1	1	1	1	1	1	1	0	1	1	1	1	1
K03929	0	0	0	0	0	0	0	0	0	0	0	0	0	0	0	0	1	0	0	0	0	0	0	0	0	0
K03976	1	1	1	1	1	1	1	1	1	1	1	1	1	1	1	1	1	1	1	1	1	1	1	1	1	1
K03977	1	1	1	1	1	1	1	1	1	1	1	1	1	1	1	1	1	1	1	1	1	1	1	1	1	1
K03978	1	1	1	1	1	1	1	1	1	1	1	1	0	0	1	1	1	1	1	1	1	1	1	1	1	1
K03979	1	1	1	1	1	1	1	1	1	1	1	1	1	1	1	1	1	1	1	1	1	1	1	1	1	1
K03980	1	1	1	1	1	1	1	1	1	1	1	1	1	1	1	1	1	1	1	1	1	1	1	1	1	1
K03981	1	1	1	1	1	1	1	1	1	1	1	1	1	1	1	1	1	1	1	1	1	1	1	1	1	1
K04030	0	0	0	1	0	0	0	0	0	0	1	0	0	0	0	0	0	0	0	0	0	0	0	0	0	0
K04042	1	1	1	1	1	1	1	1	1	1	1	1	1	1	1	1	1	1	1	1	1	1	1	1	1	1
K04043	1	1	1	1	1	1	1	1	1	1	1	1	1	1	1	1	1	1	1	1	1	1	1	1	1	1
K04044	1	1	1	1	1	1	1	1	1	1	1	1	1	1	1	1	1	1	1	1	1	1	1	0	1	1
K04045	0	0	0	1	0	0	0	0	0	0	0	0	0	0	0	0	0	0	0	0	0	0	0	0	0	0
K04047	2	1	2	1	2	2	1	1	2	2	2	2	2	2	2	2	2	2	2	2	1	2	2	2	2	2
K04066	1	1	1	0	1	1	1	1	1	1	1	1	1	1	1	1	1	1	1	1	1	1	1	1	1	1
K04068	0	0	0	1	0	0	0	0	0	0	0	0	0	0	0	0	0	0	0	0	0	0	0	0	0	0
K04073	0	0	0	0	0	0	0	0	0	0	0	0	0	0	0	1	0	0	0	0	0	0	0	0	0	0
K04075	1	1	1	1	1	1	1	1	1	1	1	1	1	1	1	1	1	1	1	1	1	1	1	1	1	1
K04077	1	1	1	1	1	1	1	1	1	1	1	1	1	1	1	1	1	1	1	1	1	1	1	1	1	1
K04078	1	1	1	1	1	1	1	1	1	1	1	1	1	1	1	1	1	1	1	1	1	1	1	1	1	1
K04079	1	1	1	1	1	1	1	1	1	1	1	1	1	1	1	1	1	1	1	1	1	1	1	1	1	1
K04082	1	1	1	1	1	1	1	1	1	1	1	1	1	1	1	1	1	1	1	1	1	1	1	1	1	1
K04083	1	1	1	1	1	1	1	1	1	1	1	1	1	1	1	1	1	1	1	1	1	1	1	1	1	1
K04084	2	2	2	1	2	3	1	1	2	2	2	2	2	2	2	2	3	2	2	2	1	2	2	2	2	2
K04085	1	1	1	1	1	1	1	1	1	1	1	1	1	1	1	1	1	1	1	1	1	1	1	1	1	1
K04096	1	1	1	1	1	1	1	1	1	1	1	1	1	1	1	1	1	1	1	1	1	0	0	1	1	1
K04097	1	0	1	0	1	1	0	0	1	0	1	1	1	1	0	1	1	1	0	1	0	0	0	1	1	1
K04103	1	1	1	1	1	1	1	1	1	1	1	1	1	1	1	1	1	1	1	1	1	1	1	1	1	1
K04485	1	1	1	1	1	1	1	1	1	1	1	1	1	1	1	1	1	1	1	1	1	1	1	1	1	1
K04487	1	1	1	1	1	1	1	1	1	1	1	1	1	1	1	1	2	1	1	1	1	1	1	1	1	1
K04488	1	1	1	1	1	1	1	1	1	1	1	1	1	1	1	1	1	1	1	1	1	1	1	1	1	1

K04561	0	0	0	0	0	0	0	1	1	0	0	0	0	0	0	0	0	0	0	0	1	0	0	1	1	0
K04564	1	1	1	1	1	1	1	1	1	1	1	1	1	1	1	1	1	1	1	1	1	1	1	1	1	1
K04565	1	0	1	1	1	1	1	1	1	1	1	1	1	1	1	1	1	1	1	1	1	1	1	1	1	1
K04567	1	1	1	1	1	1	1	1	1	1	1	1	1	1	1	1	1	1	1	1	1	1	1	1	1	1
K04568	1	1	1	1	1	1	1	1	1	1	1	1	1	1	1	1	1	1	1	1	1	1	1	1	1	1
K04691	1	1	1	1	1	1	1	1	1	1	1	1	1	1	1	1	1	1	1	1	1	1	1	1	1	1
K04744	1	1	1	1	1	1	1	1	1	1	0	0	1	1	1	1	1	1	1	1	1	1	1	1	1	1
K04752	1	1	1	1	1	1	1	1	1	1	1	1	1	1	1	1	1	1	1	1	1	1	1	1	1	1
K04755	1	1	1	1	1	1	1	1	1	1	1	1	1	1	1	1	1	1	1	1	1	1	1	1	1	1
K04758	0	0	0	0	0	0	1	0	0	0	0	0	0	0	0	0	0	0	0	0	1	0	0	0	0	0
K04759	0	0	0	0	0	0	1	0	0	0	0	0	0	0	0	0	0	0	0	0	1	0	0	0	0	0
K04761	1	1	1	1	1	1	1	1	1	1	1	1	1	1	1	1	1	1	1	1	1	1	1	1	1	1
K04762	1	1	1	1	1	1	1	1	1	1	1	1	1	1	1	1	1	1	1	1	1	1	1	1	1	1
K04763	1	1	1	1	1	1	1	1	1	1	1	1	1	1	1	1	1	1	1	1	1	1	1	1	1	1
K04764	1	1	1	1	1	1	1	1	1	1	0	0	1	1	1	1	1	1	1	1	1	1	1	1	1	1
K04765	1	1	1	1	1	1	1	1	1	1	1	1	1	1	1	1	1	1	1	1	0	1	1	1	1	1
K04771	1	1	1	1	1	1	1	1	1	1	1	1	1	1	1	1	1	1	1	1	1	1	1	1	1	1
K04773	1	1	1	1	1	1	1	1	1	1	1	1	1	1	1	1	1	1	1	1	1	1	1	1	1	1
K04774	1	1	1	1	1	1	1	1	1	1	1	1	1	1	1	1	1	1	1	1	1	1	1	1	1	1
K04940	0	0	0	0	0	0	1	0	0	0	0	0	0	0	0	0	0	0	0	0	0	0	0	0	0	0
K05020	0	0	0	1	0	0	0	0	0	0	0	0	0	0	0	0	0	0	1	0	0	0	0	0	0	0
K05297	1	1	1	1	1	1	1	1	1	1	1	1	1	1	1	1	1	1	1	1	1	1	1	1	1	1
K05365	1	1	1	1	1	1	1	1	1	1	1	1	0	1	1	1	1	1	1	1	1	1	1	1	1	1
K05366	1	1	1	1	1	1	1	1	1	1	1	1	1	1	1	1	1	1	1	1	1	1	1	0	1	1
K05396	0	1	1	0	1	0	0	0	1	1	0	0	1	1	0	1	0	1	2	0	0	1	1	1	1	0
K05515	1	1	1	1	1	1	1	1	1	1	1	1	1	1	1	1	1	1	1	1	1	1	1	1	1	1
K05516	1	1	1	1	1	1	1	1	1	1	1	0	1	1	1	1	1	1	1	1	1	1	1	1	1	1
K05520	1	0	1	0	1	1	1	1	1	1	2	1	1	1	0	1	2	1	1	1	1	1	1	1	1	1
K05523	1	0	0	0	0	1	1	1	1	0	0	0	0	0	0	0	0	0	0	1	0	1	1	1	1	0
K05524	0	1	1	1	1	1	1	1	1	1	0	1	0	1	1	1	1	2	2	1	1	2	2	1	1	1
K05527	1	1	1	1	1	1	1	1	1	1	1	1	1	1	1	1	1	1	1	1	1	1	1	1	1	1
K05539	1	1	1	1	1	1	1	1	1	1	1	1	1	1	1	1	1	1	1	1	1	1	1	1	1	1
K05540	1	1	1	1	1	1	1	1	1	1	1	1	1	1	1	1	1	1	1	1	1	1	1	1	1	1
K05541	1	1	1	1	1	1	1	1	1	1	1	1	1	1	1	1	1	1	1	1	1	1	1	1	1	1
K05548	1	0	1	0	1	0	0	0	0	1	0	0	0	0	0	0	0	1	1	0	0	1	1	0	0	1
K05549	1	0	1	0	1	0	0	0	0	1	0	0	0	0	0	0	0	1	1	0	0	1	1	0	0	1
K05550	1	0	1	0	1	0	0	0	0	1	0	0	0	0	0	0	0	1	1	0	0	1	1	0	0	1
K05589	1	1	1	1	1	1	1	1	1	1	1	1	1	1	1	1	1	1	1	1	1	1	1	1	1	1
K05595	1	1	1	2	1	1	2	2	1	1	1	1	1	1	0	1	1	1	1	1	2	1	1	1	1	1
K05685	1	1	1	1	1	1	1	0	1	1	0	0	1	1	1	1	1	1	1	1	1	1	1	1	1	1
K05710	0	0	0	0	0	0	0	0	0	0	0	0	0	0	0	1	0	0	0	0	0	0	0	0	0	0
K05712	0	0	0	0	1	0	0	0	0	0	0	0	0	0	0	0	0	0	0	0	0	0	0	0	0	0
K05713	0	0	0	0	1	0	0	0	0	0	0	0	0	0	0	0	0	0	0	0	0	0	0	0	0	0
K05777	1	0	1	0	1	0	0	0	1	1	1	1	1	1	1	1	1	1	1	1	0	0	0	1	1	0
K05778	1	0	1	0	1	0	0	0	1	1	1	0	1	1	1	1	1	1	1	1	0	0	0	1	1	0
K05779	1	0	1	0	1	0	0	0	1	1	1	1	1	1	1	1	1	1	1	1	0	0	0	1	1	0
K05782	1	0	2	0	1	0	0	0	0	2	0	0	0	0	1	0	0	2	2	0	0	1	1	0	0	1
K05783	1	0	1	0	1	0	0	0	0	1	0	0	0	0	0	0	0	1	1	0	0	1	1	0	0	1
K05784	1	0	1	0	1	0	0	0	0	1	0	0	0	0	0	0	0	1	1	0	0	1	1	0	0	1
K05786	3	1	3	2	4	4	2	2	4	3	4	4	4	4	4	4	4	4	4	4	2	3	3	4	4	4
K05788	1	1	1	1	1	1	1	1	1	1	1	1	1	1	1	1	1	1	1	1	1	1	1	1	1	1
K05791	1	2	1	1	1	1	0	2	1	1	1	0	1	1	1	1	1	1	1	1	2	1	1	1	1	1
K05792	1	1	1	0	1	1	0	1	1	1	1	1	1	1	1	1	1	2	2	1	1	1	1	1	1	1
K05794	1	0	1	0	0	0	0	1	0	0	0	0	0	0	0	0	0	0	0	0	0	0	0	0	0	1
K05795	2	2	2	0	2	2	0	2	2	2	2	2	2	2	2	2	2	2	2	2	2	2	2	2	2	2
K05807	1	1	1	1	1	1	1	1	1	1	0	1	1	1	1	1	1	1	1	1	1	1	1	1	1	1
K05808	1	1	1	1	1	1	1	1	1	1	1	1	1	1	1	1	1	1	1	1	1	1	1	1	1	1
K05810	1	1	1	1	1	1	1	1	1	1	1	0	1	1	1	1	1	1	1	1	1	1	1	1	1	1
K05819	0	0	0	0	1	0	0	0	0	0	0	0	0	0	0	1	0	0	0	0	0	0	0	0	0	0
K05825	0	0	0	0	0	0	1	0	0	0	0	0	0	0	0	0	0	0	0	0	1	0	0	0	0	0
K05832	1	1	1	1	1	1	1	1	1	1	1	1	1	1	1	1	1	1	1	1	1	1	1	1	1	1
K05833	1	1	1	1	1	1	1	1	1	1	1	1	1	1	1	1	1	1	1	1	1	1	1	1	1	1
K05836	1	0	1	0	1	1	0	1	1	1	0	0	1	1	1	1	1	1	1	1	1	1	1	1	1	1
K05837	1	1	1	1	1	1	1	1	1	1	1	1	1	1	1	1	1	1	1	1	1	1	1	0	1	1
K05845	0	0	0	0	0	0	0	0	0	0	0	0	0	0	1	0	0	0	0	0	0	0	0	0	0	0
K05846	0	0	0	0	0	0	0	0	0	0	0	0	0	0	2	0	0	0	0	0	0	0	0	0	0	0
K05847	0	0	0	0	0	0	0	0	0	0	0	0	0	0	1	0	0	0	0	0	0	0	0	0	0	0
K05896	1	1	1	1	1	1	1	1	1	1	1	1	1	1	1	1	1	1	1	1	1	1	1	1	1	1

K05916	2	0	1	0	1	0	0	0	1	1	1	1	1	1	1	1	0	1	1	1	1	0	0	1	1	1
K05952	1	1	1	1	1	1	1	1	1	1	0	1	1	1	1	1	1	1	1	1	1	1	1	1	1	1
K05982	0	0	0	0	0	0	0	0	0	0	0	2	0	0	0	0	0	0	0	0	0	0	0	0	0	0
K06001	0	0	1	1	1	0	1	1	0	1	0	0	1	1	0	1	0	1	1	0	1	1	1	0	0	0
K06006	1	0	1	0	1	0	1	1	1	1	0	0	1	1	0	1	0	1	0	1	0	1	1	1	1	1
K06016	0	0	0	0	0	0	0	0	0	0	0	0	0	0	0	1	0	0	0	0	0	0	0	0	0	0
K06024	1	1	1	1	1	1	1	1	1	1	1	1	1	1	1	1	1	1	1	1	1	1	1	1	1	1
K06041	1	1	1	1	1	1	1	1	1	1	1	1	1	1	1	1	1	1	1	1	1	1	1	1	1	1
K06076	2	2	2	3	2	2	4	3	2	2	2	2	1	3	2	2	2	2	2	3	2	2	2	2	2	1
K06131	2	2	2	2	2	1	1	2	2	2	1	1	2	2	2	2	1	2	2	2	2	1	1	2	2	1
K06133	1	1	1	1	1	1	1	1	1	1	1	1	1	1	1	1	1	1	1	1	1	1	1	1	1	1
K06134	1	1	1	1	1	1	1	1	1	1	1	1	1	1	1	1	1	1	1	1	1	1	1	1	1	1
K06136	1	0	0	0	1	1	0	0	0	0	1	1	0	0	0	0	1	0	0	0	0	0	0	0	0	1
K06137	1	0	0	0	1	1	0	0	0	0	0	1	0	0	0	0	1	0	0	0	0	0	0	0	0	1
K06138	1	0	0	0	1	1	0	0	0	0	1	0	0	0	0	0	1	0	0	0	0	0	0	0	0	1
K06139	1	0	0	0	1	1	0	0	0	0	0	1	0	0	0	0	1	0	0	0	0	0	0	0	0	1
K06143	0	0	0	0	0	0	0	0	0	0	0	0	0	0	0	0	1	0	0	0	0	0	0	0	0	1
K06149	1	1	1	1	1	2	1	1	1	1	2	2	1	1	2	1	2	2	2	1	1	2	2	1	1	2
K06153	1	1	1	1	1	1	1	1	1	1	1	1	1	1	1	1	1	1	1	1	1	1	1	1	1	1
K06158	1	1	1	1	1	1	1	1	1	1	0	1	1	1	1	1	1	1	1	1	1	1	1	1	1	1
K06159	1	0	0	0	1	1	1	1	1	1	1	1	1	1	0	1	1	0	0	1	1	1	1	1	1	1
K06168	1	1	1	1	1	1	1	1	1	1	1	1	1	1	1	1	1	1	1	1	1	1	1	1	1	1
K06169	1	1	1	1	1	1	1	1	1	1	1	1	1	1	1	1	1	1	1	1	1	1	1	1	1	1
K06173	1	1	1	1	1	1	1	1	1	1	1	1	1	1	1	1	1	1	1	1	1	1	1	0	1	1
K06175	0	0	1	0	1	0	1	1	1	1	1	1	1	1	0	1	1	1	1	1	1	1	1	1	1	1
K06176	1	1	1	1	1	1	1	1	1	1	1	1	1	1	1	1	1	1	1	1	1	1	1	1	1	1
K06177	1	1	1	1	1	1	1	1	1	1	1	0	1	1	1	1	1	1	1	1	1	1	1	1	1	1
K06178	1	1	1	1	1	1	1	1	1	1	1	1	1	1	1	1	1	1	1	1	1	1	1	1	1	1
K06179	1	1	1	1	1	1	1	1	1	1	1	1	1	1	1	1	1	1	1	1	1	1	1	1	1	1
K06180	1	1	1	1	1	1	1	1	1	1	1	1	1	1	1	1	1	1	1	1	1	1	1	1	1	1
K06181	1	1	1	1	1	1	1	1	1	1	1	1	1	1	1	1	1	1	1	1	1	1	1	1	1	1
K06182	1	1	1	0	1	1	0	0	1	1	1	1	1	1	0	1	1	1	1	1	0	1	1	1	1	1
K06183	1	1	1	1	1	1	1	1	1	1	1	1	1	1	1	1	1	1	1	1	1	1	1	1	1	1
K06186	1	1	1	1	1	1	1	1	1	1	1	1	1	1	1	1	1	1	1	1	1	1	1	1	1	1
K06187	1	1	1	1	1	1	1	1	1	1	1	1	1	1	1	1	1	1	1	1	1	1	0	1	1	1
K06188	0	0	0	1	0	0	1	1	0	0	0	0	0	0	0	0	0	0	0	0	0	0	0	0	0	0
K06189	1	1	1	1	1	1	1	1	1	1	1	1	1	1	1	1	1	1	1	1	1	1	1	1	1	1
K06190	1	1	1	1	1	1	1	1	1	1	1	1	1	1	1	1	1	1	1	1	1	1	1	1	1	1
K06193	1	1	1	1	1	1	1	1	1	1	1	1	1	1	1	1	1	1	1	1	1	1	1	1	1	1
K06194	1	1	1	1	1	1	1	1	1	1	1	1	1	1	1	1	1	1	1	1	1	1	1	1	1	1
K06197	0	0	0	0	0	0	0	0	1	0	0	0	0	0	1	0	0	1	2	0	0	1	1	1	1	1
K06198	0	0	0	0	0	0	0	0	1	0	0	0	0	0	0	0	0	0	0	0	0	0	0	1	1	0
K06199	1	1	1	1	1	1	1	1	1	1	1	1	1	1	1	1	1	1	1	1	1	1	1	1	1	1
K06200	1	1	1	1	1	1	1	1	1	1	1	1	1	1	1	1	1	1	1	1	1	1	1	1	1	1
K06204	1	1	1	1	1	1	1	1	1	1	1	1	1	1	1	1	1	1	1	1	1	1	1	1	1	1
K06207	1	1	1	1	1	1	1	1	1	1	1	1	1	1	2	1	1	1	1	1	1	1	1	1	1	1
K06211	1	0	1	1	1	1	1	1	1	1	1	1	1	1	0	1	1	1	1	1	1	1	1	1	1	1
K06213	1	1	1	2	1	1	2	2	2	1	1	1	2	2	1	2	1	2	2	1	2	1	1	2	2	1
K06217	1	1	1	1	1	1	1	1	1	1	1	1	1	1	1	1	1	1	1	1	1	1	1	1	1	1
K06218	1	1	1	0	0	1	0	0	1	1	0	0	0	1	0	1	0	1	1	1	0	1	1	1	1	0
K06219	1	1	1	1	1	1	1	1	1	1	1	1	1	1	1	1	1	1	1	1	1	1	1	1	1	1
K06221	0	0	0	0	0	0	0	0	0	0	0	0	0	0	0	0	0	0	0	0	0	1	1	0	0	0
K06222	0	0	0	0	0	0	0	0	0	0	0	1	0	0	0	0	0	0	0	0	1	0	0	0	0	0
K06223	0	0	0	0	0	0	1	0	0	0	0	0	0	0	0	0	0	2	0	0	0	1	1	0	0	2
K06287	1	1	1	1	1	1	1	1	1	1	1	1	1	1	1	1	1	1	1	1	1	1	1	1	1	1
K06445	1	1	1	1	1	1	1	1	1	1	1	1	1	1	1	1	1	1	1	1	1	1	1	1	1	1
K06446	0	1	1	0	1	1	0	0	1	1	0	0	0	0	0	0	1	1	1	1	0	1	1	1	1	1
K06518	0	0	0	0	1	0	0	0	0	0	0	0	0	0	0	0	0	0	0	0	1	0	0	0	0	1
K06596	1	1	1	0	1	1	0	0	1	1	1	0	1	1	1	1	1	1	1	1	1	1	1	1	1	1
K06597	1	0	0	0	1	1	0	0	1	1	1	1	1	1	1	1	1	1	1	1	0	0	0	1	1	1
K06860	0	0	1	0	0	0	0	0	0	0	0	0	0	0	0	0	0	0	0	0	0	0	0	0	0	0
K06861	1	1	1	1	1	1	1	1	1	1	1	1	1	1	1	1	1	1	1	1	1	1	1	1	1	1
K06867	0	0	0	0	0	0	1	0	0	0	0	0	0	0	0	0	0	0	0	0	0	0	0	0	0	0
K06871	0	0	0	0	0	0	0	0	0	0	0	0	0	0	0	0	0	1	0	0	0	0	0	0	0	0
K06872	1	1	1	1	1	1	1	1	1	1	1	1	1	1	1	1	1	1	1	1	1	1	1	1	1	1
K06876	0	0	0	0	0	0	0	0	1	0	0	0	0	0	0	0	0	0	0	0	0	1	1	1	1	0
K06879	1	1	1	1	1	1	1	1	1	1	1	1	1	1	1	1	1	1	1	1	1	1	1	1	1	1
K06889	0	0	0	0	1	0	0	0	0	0	0	0	0	0	0	0	0	0	0	0	0	1	1	0	0	1

K06891	1	1	1	1	1	1	1	1	1	1	1	1	1	1	1	1	1	1	1	1	1	1	1	1	1	1
K06893	1	0	0	0	0	0	0	0	0	0	0	0	0	0	0	0	0	0	0	0	0	0	0	0	0	0
K06895	1	0	1	1	1	1	1	1	1	1	1	1	1	1	1	1	1	1	1	1	1	1	1	1	1	1
K06899	0	0	0	0	0	0	0	0	1	0	0	0	0	0	0	0	0	0	0	0	0	0	0	1	1	0
K06900	0	1	0	0	0	0	0	0	0	0	0	0	0	0	0	0	0	0	0	0	0	0	0	0	0	0
K06901	1	0	1	1	1	1	1	1	1	1	1	1	1	1	1	1	1	1	1	1	1	1	1	1	1	1
K06903	1	0	0	1	0	1	0	0	0	1	0	0	0	0	0	0	0	0	0	0	0	0	0	0	0	0
K06904	0	1	0	0	0	1	0	0	0	0	0	0	0	0	0	0	0	0	0	0	0	0	0	0	0	0
K06905	0	0	0	1	0	1	0	0	0	1	0	0	0	0	0	0	0	0	0	0	0	0	0	0	0	0
K06906	0	0	0	0	0	1	0	0	0	1	0	0	0	0	0	0	0	0	0	0	0	0	0	0	0	0
K06907	1	0	0	1	0	1	0	0	0	2	0	0	0	0	0	0	0	0	0	0	0	0	0	0	0	0
K06908	1	0	0	1	0	1	0	0	0	1	0	0	0	0	0	0	0	0	0	0	0	0	0	0	0	0
K06909	0	1	0	0	1	0	1	0	0	0	0	0	0	0	0	0	0	0	0	0	0	0	0	0	0	0
K06910	1	0	0	0	0	0	0	0	0	0	1	1	1	1	0	1	2	0	0	0	0	0	0	0	0	0
K06911	2	1	1	4	3	4	4	4	5	1	3	3	3	2	1	3	3	3	3	1	4	3	3	5	5	3
K06915	0	0	0	0	0	0	0	0	1	0	0	0	0	0	0	1	0	0	0	0	0	0	0	1	1	0
K06916	1	1	1	1	1	1	1	1	1	1	1	1	1	1	1	1	1	1	1	1	1	1	1	1	1	1
K06919	0	2	0	0	0	1	0	0	0	0	0	0	0	0	0	0	0	0	0	0	0	0	0	0	0	2
K06920	1	1	1	1	1	1	1	1	1	1	1	1	1	1	1	1	1	2	1	1	1	1	1	1	1	1
K06923	1	1	1	1	1	1	1	1	1	1	1	1	1	1	1	1	1	1	1	1	1	1	1	1	1	1
K06925	1	1	1	1	1	1	1	1	1	1	1	1	1	1	1	1	1	1	1	1	1	1	1	1	1	1
K06926	0	0	1	0	1	0	0	0	0	0	0	0	0	0	1	0	0	0	1	0	0	0	0	0	0	0
K06938	1	1	1	1	1	1	1	1	1	1	1	0	0	1	1	1	1	1	1	1	1	1	1	1	1	1
K06941	1	1	1	1	1	1	1	1	1	1	1	1	1	1	1	1	1	1	1	1	1	1	1	1	1	1
K06942	1	1	1	1	1	1	1	1	1	1	1	1	1	1	1	1	1	1	1	3	1	1	1	1	1	1
K06945	0	0	0	0	1	1	0	0	0	0	0	0	1	2	0	1	0	0	0	0	0	1	1	0	0	1
K06949	1	1	1	1	1	1	1	1	1	1	1	1	1	1	1	1	1	1	1	1	1	1	1	1	1	1
K06950	1	1	1	1	1	1	1	1	1	1	1	1	1	1	1	1	1	1	1	1	1	1	1	1	1	1
K06955	1	0	1	0	1	1	1	1	1	1	1	1	1	1	1	1	1	1	1	1	1	1	1	1	1	1
K06956	0	0	0	1	0	0	1	1	0	0	0	0	0	0	0	0	0	0	0	0	1	0	0	0	0	0
K06958	1	1	1	1	1	1	1	1	1	1	1	1	1	1	1	1	1	1	1	1	1	1	1	1	1	1
K06959	1	1	1	1	1	1	1	1	1	1	1	1	1	1	1	1	1	1	1	1	1	1	1	1	1	1
K06966	1	1	1	2	1	1	1	1	1	1	1	1	1	1	1	1	1	2	2	1	1	1	1	1	1	1
K06970	1	1	1	1	1	1	1	1	1	1	1	1	1	1	1	1	1	1	1	1	1	1	1	1	1	1
K06972	1	1	1	1	1	1	1	1	1	1	1	1	1	1	1	1	1	1	1	1	1	1	1	1	1	1
K06975	1	1	1	2	1	1	2	2	2	1	1	1	2	2	1	2	1	2	2	1	2	2	2	2	2	2
K06980	1	1	1	1	1	1	1	1	1	1	1	1	1	1	1	1	1	1	1	1	1	1	1	1	1	1
K06989	0	0	1	0	0	0	0	0	0	1	0	0	0	0	0	0	0	0	0	0	0	0	0	0	0	0
K06990	0	0	0	1	0	0	1	1	0	0	0	0	0	0	0	0	0	0	0	0	1	0	0	0	0	0
K06992	0	0	0	0	0	0	1	0	0	0	0	0	0	0	0	0	0	0	0	0	0	0	0	0	0	0
K06996	0	0	0	0	0	0	1	1	0	0	0	0	0	0	0	0	0	0	0	0	0	0	0	0	0	0
K06997	1	1	1	1	1	1	1	1	1	1	0	1	1	1	1	1	1	1	1	1	1	1	1	1	1	1
K06999	1	1	1	1	1	1	1	1	1	1	1	1	1	1	1	1	1	1	1	1	1	1	1	1	1	1
K07000	1	1	1	1	1	1	1	1	1	1	1	1	1	1	1	1	1	1	1	1	1	1	1	1	1	1
K07001	1	1	1	1	1	1	1	1	1	1	2	1	1	1	1	1	1	3	3	1	1	1	1	1	1	1
K07007	1	1	1	1	1	1	1	1	1	1	1	1	1	1	1	1	1	1	1	1	1	1	1	0	1	1
K07008	1	1	1	1	1	1	1	1	1	1	1	1	1	1	1	1	2	1	1	1	1	1	1	1	1	2
K07011	0	0	1	0	0	0	0	0	0	1	0	0	0	0	0	0	0	0	0	0	0	0	0	0	0	0
K07012	0	0	0	2	0	1	1	0	0	1	0	0	0	0	2	1	0	0	0	0	0	0	0	0	0	0
K07014	0	0	0	0	0	0	0	0	0	0	0	1	0	0	0	0	0	0	0	0	0	0	0	0	0	0
K07017	0	0	0	0	0	0	0	0	0	0	0	0	0	0	0	1	0	0	0	0	1	0	0	0	0	0
K07018	1	1	1	1	1	1	1	1	1	1	1	1	1	1	1	1	1	1	1	1	1	1	1	1	1	1
K07019	1	1	1	1	1	1	1	1	1	1	1	1	1	1	1	1	1	1	1	1	1	1	1	1	1	1
K07023	1	1	1	1	1	1	1	1	1	1	1	1	1	1	0	1	1	0	0	1	1	1	1	1	1	1
K07024	1	1	1	1	1	1	1	1	1	1	1	1	1	1	1	1	1	1	1	1	1	1	1	1	1	1
K07026	0	0	1	0	0	0	0	0	0	0	0	0	0	0	0	0	0	0	0	0	0	0	0	0	0	0
K07027	0	0	0	1	0	0	1	1	0	0	0	0	0	0	0	0	0	0	0	0	1	0	0	0	0	0
K07035	1	1	1	2	1	1	1	1	1	1	1	1	1	1	1	1	1	1	1	1	1	1	1	1	1	1
K07038	1	0	0	0	0	0	0	0	0	0	0	0	0	0	0	0	0	0	0	0	0	1	1	0	0	0
K07039	1	1	1	1	1	1	1	1	1	1	1	1	1	1	1	1	1	1	1	1	1	1	1	1	1	1
K07040	1	1	1	1	1	1	1	1	1	1	1	1	1	1	1	1	1	1	1	1	1	1	1	1	1	1
K07042	1	1	1	1	1	1	1	1	1	1	1	1	1	1	1	1	1	1	1	1	1	1	1	1	1	1
K07043	3	2	3	1	2	2	3	2	2	2	1	2	2	4	3	2	2	2	3	2	3	2	2	2	2	2
K07046	0	0	0	0	0	0	0	0	0	0	1	0	0	0	0	0	0	0	0	0	0	0	0	0	0	0
K07047	0	0	0	3	1	0	3	1	0	0	0	0	1	0	0	1	0	1	2	0	2	0	0	0	0	0
K07052	1	1	1	1	1	1	1	1	1	1	1	1	1	1	1	1	1	1	1	1	1	1	1	1	1	1
K07053	1	1	1	1	1	1	1	1	1	1	1	1	1	1	1	1	1	1	1	1	1	1	1	1	1	1
K07054	1	1	1	1	1	1	1	1	1	1	1	1	1	1	0	1	1	1	1	1	1	1	1	1	1	1

K07056	1	1	1	1	1	1	1	1	1	1	1	1	1	1	1	1	1	1	1	1	1	1	1	1	1	1
K07058	2	1	1	1	2	2	1	1	2	1	2	2	2	2	1	2	2	2	2	2	1	2	2	2	2	2
K07061	0	0	0	1	0	0	0	0	0	0	0	0	0	1	0	0	0	0	0	0	0	0	0	0	0	0
K07062	0	0	0	1	0	0	0	0	0	0	0	0	0	0	0	0	0	0	0	0	0	0	0	0	0	0
K07070	1	1	1	1	1	1	1	1	1	1	1	0	1	1	1	1	1	1	1	1	1	1	1	1	1	1
K07071	1	0	1	0	1	1	1	1	2	1	1	1	1	1	1	1	1	1	1	1	1	1	1	2	2	1
K07078	1	1	1	1	2	1	1	1	1	1	1	1	2	2	1	3	1	1	1	1	1	1	1	1	1	1
K07080	1	1	1	2	1	1	1	2	1	0	1	1	1	1	1	1	1	1	1	1	0	0	0	1	1	1
K07082	1	1	1	1	1	1	1	1	1	1	1	1	1	1	1	1	1	1	1	1	1	1	1	1	1	1
K07084	1	0	1	1	1	1	1	1	1	1	1	1	1	1	1	1	1	1	1	1	2	1	1	1	1	2
K07085	0	0	0	0	1	0	0	0	0	0	0	0	0	0	0	0	0	0	0	0	0	0	0	0	0	0
K07088	1	1	1	0	1	1	2	2	1	1	1	1	1	1	1	1	1	2	2	1	3	1	1	1	1	1
K07090	5	3	3	3	5	3	4	4	3	4	3	4	3	4	4	3	4	3	4	3	4	4	4	3	3	4
K07091	1	1	1	1	1	1	1	1	1	1	1	1	1	1	1	1	1	1	1	1	1	1	1	1	1	1
K07093	1	1	1	1	1	1	1	1	1	1	1	1	1	1	1	1	1	1	1	1	1	1	1	1	1	1
K07098	1	0	2	0	2	1	0	0	2	2	1	1	2	2	2	2	1	2	2	2	0	1	1	2	2	1
K07102	1	1	1	1	1	1	1	1	1	1	1	1	1	1	1	1	1	1	1	1	1	1	1	1	1	1
K07107	2	3	3	1	3	3	1	1	3	3	1	1	2	2	3	2	3	3	3	3	1	2	2	3	3	3
K07112	2	3	2	2	2	2	3	2	2	2	3	3	2	2	3	2	3	3	3	2	2	2	2	2	2	3
K07113	1	1	1	1	1	1	1	1	1	1	1	1	1	1	1	1	1	1	1	1	1	1	1	1	1	1
K07114	0	0	0	0	0	0	1	1	1	0	1	1	0	0	1	0	1	0	0	1	1	1	1	1	1	1
K07115	1	1	1	1	1	1	1	1	1	1	1	1	1	1	1	1	1	1	1	1	1	1	1	1	1	1
K07116	0	0	1	0	0	0	0	0	1	1	0	0	1	1	0	1	1	1	1	1	0	1	1	1	1	1
K07119	2	0	2	1	2	2	1	1	2	2	2	2	2	2	2	2	2	1	1	2	1	2	2	2	2	2
K07120	0	0	0	0	0	0	0	0	0	1	0	0	0	0	0	0	0	0	0	0	0	0	0	0	0	0
K07126	0	1	2	0	1	0	1	1	2	0	0	0	4	2	3	1	0	0	1	1	1	1	1	2	2	4
K07127	0	0	1	0	2	1	0	0	0	1	1	1	2	2	1	2	1	1	1	0	0	1	1	0	0	1
K07131	1	0	0	0	1	1	0	0	1	1	1	0	1	1	1	1	1	1	1	1	0	1	1	1	1	1
K07137	1	1	1	0	1	0	0	0	1	1	0	0	1	1	0	1	0	1	1	1	0	0	0	1	1	0
K07141	1	0	1	0	1	1	1	1	1	1	1	1	1	1	0	1	1	1	1	1	1	1	1	1	1	1
K07146	1	1	1	1	1	1	1	1	1	1	1	1	1	1	1	1	1	1	1	1	1	1	1	1	1	1
K07152	0	0	0	0	0	0	0	1	0	0	0	0	0	0	0	0	0	0	0	0	0	0	0	0	0	0
K07154	0	1	0	0	0	0	0	0	0	0	0	1	1	0	1	0	0	1	1	0	0	0	0	0	0	0
K07160	0	1	0	0	0	0	0	0	1	0	1	0	0	0	1	0	1	2	2	1	1	1	1	1	1	1
K07170	0	0	0	1	0	0	1	1	0	0	0	0	0	0	0	0	0	0	0	0	1	0	0	0	0	0
K07182	1	1	1	1	1	1	1	1	1	1	1	1	1	1	1	1	1	1	1	1	1	1	1	1	1	1
K07213	1	0	0	0	0	0	0	1	0	1	0	0	1	1	0	1	0	0	0	0	0	1	1	0	0	0
K07215	0	0	0	0	0	0	0	0	0	0	0	0	0	1	0	0	0	0	0	0	0	0	0	0	0	0
K07222	0	0	0	0	1	0	0	0	0	0	0	0	0	0	0	0	0	0	0	0	0	0	0	0	0	1
K07225	0	0	0	1	0	0	1	0	0	1	0	0	1	1	0	1	0	0	0	0	1	1	1	0	0	0
K07230	0	0	0	1	0	0	0	0	0	0	0	0	0	0	0	0	0	0	0	0	0	0	0	0	0	0
K07231	1	1	1	1	1	1	1	1	1	1	1	1	1	1	1	1	1	1	1	1	1	1	1	1	1	1
K07233	1	1	1	0	0	2	0	1	1	1	2	1	1	0	1	1	2	2	1	1	1	2	2	1	1	1
K07234	2	1	0	1	1	0	1	1	1	0	0	0	1	1	1	1	1	1	1	1	1	1	1	1	1	0
K07235	1	1	1	1	1	1	1	1	1	1	1	1	1	1	1	1	1	1	1	1	1	1	1	1	1	1
K07236	1	1	1	1	1	1	1	1	1	1	1	1	1	1	1	1	1	1	1	1	1	1	1	1	1	1
K07237	1	1	1	1	1	1	1	1	1	1	1	1	1	0	1	1	1	1	1	1	1	1	1	1	1	1
K07238	0	0	0	1	0	1	1	1	1	0	0	0	0	0	2	0	0	0	0	0	0	2	2	1	1	1
K07240	1	0	1	0	0	0	0	0	0	1	0	0	0	0	0	0	0	0	1	0	0	0	0	0	0	0
K07243	0	0	0	1	0	0	0	0	0	0	0	0	0	0	0	0	0	0	0	0	0	0	0	0	0	0
K07248	0	0	0	0	0	0	0	0	0	0	0	0	0	0	0	0	1	0	0	0	0	0	0	0	0	0
K07250	1	1	1	1	1	1	1	0	1	1	1	1	1	1	1	1	1	1	1	1	1	0	0	0	1	1
K07258	1	1	1	1	1	1	1	1	1	1	1	1	1	1	1	1	1	1	1	1	1	1	1	1	1	1
K07259	1	0	1	1	1	1	1	1	1	1	1	1	1	1	1	1	1	1	1	1	1	1	1	1	1	1
K07262	1	1	1	1	1	1	1	1	1	1	1	1	1	1	1	1	1	1	1	1	1	1	1	1	1	1
K07263	2	2	2	2	2	2	2	2	2	2	1	2	2	2	2	2	2	2	2	2	2	2	2	2	2	2
K07265	0	0	0	0	1	0	0	0	1	0	1	1	1	1	0	1	1	1	1	1	0	0	0	1	1	1
K07266	0	0	0	0	1	0	0	0	0	0	1	0	1	1	0	3	1	1	3	1	0	0	0	0	0	1
K07270	0	1	0	2	2	0	0	1	2	1	1	1	2	0	1	2	0	0	1	0	2	0	0	2	2	0
K07274	1	1	1	1	1	1	1	1	1	1	1	1	1	1	1	1	1	1	1	1	1	1	1	1	1	1
K07275	1	1	1	1	1	1	1	1	1	1	1	1	1	1	1	1	1	1	1	1	1	1	1	1	1	1
K07277	1	1	1	1	1	1	1	1	1	1	1	1	1	1	1	1	1	1	1	1	1	1	1	1	1	1
K07278	1	1	1	1	1	1	1	1	1	1	0	1	1	1	1	1	1	1	1	1	1	1	1	1	1	1
K07301	1	0	1	1	1	1	1	1	1	1	1	1	1	1	1	1	1	2	1	1	1	1	1	1	1	2
K07304	1	1	1	1	1	1	1	1	1	1	1	1	1	1	1	1	1	1	1	1	1	1	1	1	1	1
K07305	1	1	1	1	1	1	1	1	1	1	1	1	1	1	1	1	1	1	1	1	1	1	1	1	1	1
K07313	0	0	0	0	0	0	0	1	0	0	0	0	0	0	0	0	0	0	0	0	0	0	0	0	0	0
K07316	1	0	0	0	0	0	0	1	0	1	0	0	0	0	0	0	0	1	1	0	0	1	1	0	0	0

K07318	0	0	0	0	0	0	0	0	0	0	0	0	0	0	0	0	0	1	0	0	0	0	0	0	0	0
K07320	1	1	1	1	1	1	1	1	1	1	1	1	1	1	1	1	1	1	1	1	1	1	1	1	1	1
K07334	0	0	0	0	0	0	0	0	1	0	0	0	0	0	1	0	0	0	0	2	0	0	0	1	1	0
K07336	0	0	0	0	0	0	0	0	0	0	0	0	0	0	0	0	0	0	0	0	1	0	0	0	0	0
K07338	1	1	1	1	1	1	1	1	1	1	1	1	1	1	1	1	1	1	1	1	1	1	1	1	1	1
K07340	1	1	1	1	1	1	1	1	1	1	1	1	1	1	1	1	1	1	1	1	1	1	1	1	1	1
K07345	0	0	0	0	0	0	0	1	0	0	0	0	0	0	0	0	0	0	0	0	1	0	0	0	0	0
K07346	1	0	1	0	1	0	0	0	0	1	1	0	0	0	1	0	0	1	1	1	1	0	0	0	0	1
K07347	1	0	1	0	1	0	0	1	0	1	0	0	0	0	1	0	0	1	1	1	1	0	0	0	0	1
K07390	1	1	1	1	1	1	1	1	1	1	1	1	1	1	1	1	1	1	1	1	1	1	1	1	1	1
K07391	1	1	1	1	1	1	1	1	1	1	1	1	1	1	1	1	1	1	1	1	1	1	1	1	1	1
K07393	1	1	1	1	1	1	1	1	1	1	1	1	1	2	3	1	1	1	1	1	1	1	1	1	1	1
K07394	1	1	1	1	1	1	1	1	1	1	1	1	1	1	1	1	1	1	1	1	1	1	1	1	1	1
K07395	1	0	0	0	0	0	0	1	1	0	1	1	0	0	0	0	1	1	1	1	0	0	0	1	1	1
K07397	1	1	1	1	1	1	1	1	1	1	1	1	1	1	1	1	1	1	1	1	1	1	1	1	1	1
K07400	1	1	1	1	1	1	1	1	1	1	1	1	1	1	1	1	1	1	1	1	1	1	1	1	1	1
K07402	0	0	1	0	0	1	0	0	0	1	1	1	0	0	1	0	1	1	1	0	0	1	1	0	0	0
K07447	1	1	1	1	1	1	1	1	1	1	1	1	1	1	1	1	1	1	1	1	1	1	1	1	1	1
K07448	0	0	0	0	0	0	0	0	0	0	0	0	0	1	0	0	0	0	0	0	0	0	0	0	0	0
K07451	0	1	1	0	0	0	0	0	0	1	0	1	0	0	0	0	0	0	0	1	0	0	0	0	0	1
K07452	1	1	2	1	1	1	1	2	1	1	1	1	2	2	1	1	1	2	1	1	2	2	2	1	1	1
K07453	1	0	0	0	0	0	0	0	0	0	0	0	0	0	0	0	0	0	0	0	0	0	0	0	0	0
K07460	1	1	1	1	1	1	0	1	1	1	1	1	1	1	1	1	1	1	1	1	1	1	1	1	1	1
K07462	1	1	1	1	1	1	1	1	1	1	1	1	1	1	1	1	1	1	1	1	1	1	1	1	1	1
K07464	0	0	0	1	0	1	1	0	0	0	0	0	0	0	1	0	0	0	0	0	0	0	0	0	0	0
K07473	0	0	0	0	0	0	0	0	0	0	0	0	0	0	0	0	0	0	0	0	0	0	0	0	0	1
K07474	0	0	0	0	0	0	1	0	0	0	0	0	0	0	0	0	0	0	0	0	0	0	0	0	0	0
K07478	1	1	1	1	1	1	1	1	1	1	1	1	1	1	1	1	1	1	1	1	1	1	1	1	1	1
K07480	0	0	0	0	0	0	0	3	0	0	0	0	0	0	0	0	0	0	0	0	0	0	0	0	0	0
K07483	1	8	1	0	3	4	5	2	26	3	0	1	1	2	7	4	2	5	1	2	13	9	6	21	31	1
K07488	1	0	0	0	2	1	1	1	0	0	0	0	0	3	0	2	0	0	8	0	0	11	13	0	0	0
K07491	0	1	1	2	0	0	1	0	1	0	0	0	0	0	12	0	0	0	0	0	0	1	1	1	1	1
K07496	0	0	1	0	0	1	0	2	0	1	0	0	0	2	20	0	0	0	0	0	0	1	1	0	0	0
K07497	0	12	1	0	2	2	0	1	11	0	0	2	0	5	10	1	0	0	1	0	12	0	0	7	10	1
K07501	1	1	1	1	1	1	1	1	1	1	1	1	1	1	1	1	1	1	1	1	1	1	1	1	1	1
K07507	1	0	2	0	1	0	1	1	1	1	1	0	1	1	0	1	1	1	1	0	1	1	1	1	1	0
K07552	1	1	1	1	1	1	1	1	1	1	1	1	1	1	1	1	1	1	1	1	1	1	1	1	1	1
K07560	1	1	1	1	1	1	1	1	1	1	1	1	1	1	1	1	1	1	1	1	1	1	1	1	1	1
K07566	1	1	1	1	1	1	1	1	1	1	1	1	1	1	1	1	1	1	1	1	1	1	1	1	1	1
K07568	1	1	1	1	1	1	1	1	1	1	1	1	1	1	1	1	1	1	1	1	1	1	1	1	1	1
K07574	1	1	1	1	1	1	1	1	1	1	1	1	1	1	1	1	1	1	1	1	1	1	1	1	1	1
K07636	1	1	1	1	1	1	1	1	1	1	1	1	1	1	1	1	1	1	1	1	1	1	1	1	1	1
K07638	1	1	1	1	1	1	1	1	1	1	0	1	1	1	1	1	1	1	1	1	1	1	1	1	1	1
K07639	1	1	1	1	1	1	1	1	1	1	1	1	1	1	1	1	1	1	1	1	1	1	1	1	1	1
K07640	1	0	1	0	1	1	1	0	1	1	1	0	1	1	0	1	1	1	1	1	0	1	1	1	1	1
K07641	1	0	0	1	1	1	1	1	1	0	1	1	1	1	1	1	1	1	1	1	1	1	1	1	1	1
K07644	1	1	1	0	1	2	0	1	1	1	2	2	2	1	1	2	2	2	1	1	1	2	2	1	1	1
K07645	0	1	0	0	1	1	1	1	1	1	0	1	0	0	1	0	0	1	0	0	1	1	1	1	1	1
K07646	0	0	0	0	0	0	0	0	0	1	0	0	0	0	0	0	0	0	0	1	0	0	0	0	0	1
K07657	1	1	1	1	1	1	1	1	1	1	1	1	1	1	1	1	1	1	1	1	1	1	1	1	1	1
K07659	1	1	1	1	1	1	1	1	1	1	1	1	1	1	1	1	1	1	1	1	1	1	1	1	1	1
K07661	1	1	1	1	1	1	1	1	1	1	1	1	1	1	1	1	1	1	1	1	1	1	1	1	1	1
K07662	1	0	1	1	1	1	1	1	1	1	1	1	1	1	0	1	1	1	1	1	1	1	1	1	1	1
K07663	1	0	0	1	1	1	1	1	1	0	1	1	1	1	1	1	1	1	1	1	1	1	1	1	1	1
K07665	1	1	1	0	1	2	0	1	1	1	2	2	2	1	1	2	2	2	1	1	1	2	2	1	1	1
K07666	0	1	0	0	1	1	1	1	1	1	0	1	0	0	1	0	0	1	0	0	1	1	1	1	1	1
K07667	0	0	0	0	0	0	0	0	0	1	0	0	0	0	0	0	0	0	0	1	0	0	0	0	0	1
K07673	1	1	0	1	0	0	0	1	0	0	0	0	1	1	1	1	0	1	1	0	1	0	0	0	0	0
K07678	1	1	1	1	1	1	1	1	1	1	1	0	1	1	1	1	1	1	1	1	1	1	1	1	1	1
K07684	1	1	0	1	0	0	0	1	0	0	0	0	1	1	1	1	0	1	1	0	1	0	0	0	0	0
K07705	0	0	1	0	0	0	0	0	0	0	0	0	0	0	0	0	0	1	1	0	0	0	0	0	0	0
K07708	1	1	1	1	1	1	1	1	1	1	1	1	1	1	1	1	1	1	1	1	1	1	1	1	1	1
K07712	1	1	1	1	1	1	1	1	1	1	1	1	1	1	1	1	1	1	1	1	1	1	1	0	1	1
K07722	0	0	0	1	0	0	1	0	0	0	0	0	0	0	0	0	0	0	0	0	0	0	0	0	0	0
K07726	0	0	0	1	0	1	0	0	0	0	0	0	0	0	0	0	0	0	0	0	0	1	1	0	0	0
K07727	0	0	1	1	1	3	2	1	2	2	2	0	1	2	1	4	3	3	2	0	2	3	2	2	2	1
K07733	1	1	2	0	1	1	0	0	2	0	2	0	0	1	0	1	0	1	1	0	0	0	0	2	2	1
K07734	1	0	0	1	1	0	1	0	1	0	0	0	1	1	2	1	0	0	0	0	0	0	0	1	1	0

K07735	1	1	1	1	1	1	1	1	1	1	1	1	1	1	1	1	1	1	1	1	1	1	1	1	1	1
K07738	1	1	1	1	1	1	1	1	1	1	1	1	1	1	1	1	1	1	1	1	1	1	1	1	1	1
K07741	0	0	0	0	0	0	0	0	0	0	0	0	0	0	0	0	0	0	0	0	0	0	0	0	0	1
K07749	1	1	1	1	1	1	2	1	1	1	1	1	1	1	1	1	1	1	1	1	1	0	0	1	1	1
K07793	1	0	1	0	1	0	0	0	0	1	0	0	0	0	0	0	1	0	0	0	0	0	0	0	0	1
K07794	1	0	1	0	1	0	0	0	0	1	0	1	0	1	0	0	1	0	0	0	0	0	0	0	0	1
K07795	1	0	1	0	1	0	0	0	0	1	0	1	0	1	0	0	1	0	0	0	0	0	0	0	0	1
K07807	0	1	1	1	0	0	0	0	0	1	0	0	0	0	0	0	0	0	1	0	0	0	0	0	0	0
K07811	0	0	0	1	0	0	1	0	0	0	0	0	0	0	0	0	0	0	0	0	1	0	0	0	0	0
K07862	1	1	1	1	1	1	1	1	1	1	0	1	2	2	1	2	1	1	1	1	1	1	1	1	1	1
K08082	1	0	1	0	1	1	0	0	1	1	1	1	1	1	1	1	1	1	1	1	0	1	1	1	1	1
K08083	1	0	1	0	1	1	0	0	1	1	1	1	1	1	1	1	1	1	1	1	0	1	1	1	1	1
K08084	2	2	2	1	2	2	1	1	2	2	2	2	2	2	3	2	2	2	2	2	1	2	2	2	2	2
K08086	1	1	1	0	1	1	0	0	1	1	0	1	1	1	1	1	1	1	1	1	0	1	1	1	1	1
K08159	1	1	1	1	1	1	1	1	1	1	1	1	1	1	1	1	1	1	1	1	1	1	1	1	1	1
K08195	0	0	0	0	0	0	0	0	0	0	0	0	0	0	0	0	0	0	0	0	1	0	0	0	0	0
K08196	0	1	1	0	1	1	0	0	1	1	0	0	0	0	0	0	1	1	1	1	0	0	0	1	1	1
K08218	1	1	1	1	1	1	1	1	1	1	1	1	1	1	1	1	1	1	1	1	1	1	1	1	1	1
K08279	0	0	0	0	0	0	0	0	0	1	0	0	0	0	0	0	0	1	0	0	0	0	0	0	0	0
K08296	1	1	1	1	1	1	1	1	1	1	1	1	1	1	1	1	1	1	1	1	1	1	1	1	1	1
K08299	0	0	0	0	0	0	0	0	0	1	0	0	0	0	0	0	0	1	0	0	0	0	0	0	0	0
K08300	1	1	1	1	1	1	1	1	1	1	1	0	1	1	1	1	1	1	1	1	1	1	1	1	1	1
K08301	1	1	1	1	1	1	1	1	1	1	1	1	1	1	1	1	1	1	1	1	1	1	1	1	1	1
K08303	0	0	0	1	0	0	1	1	0	0	0	0	0	0	0	0	0	0	0	0	1	0	0	0	0	0
K08309	1	1	1	1	1	1	1	1	1	1	1	1	1	1	1	1	1	1	1	1	1	1	1	1	1	1
K08311	1	1	1	1	1	1	1	1	1	1	1	1	1	1	1	1	1	1	1	1	1	1	1	1	1	1
K08316	1	1	1	1	1	1	1	1	1	1	1	1	1	1	1	1	1	1	1	1	1	1	1	1	1	1
K08325	1	1	1	1	1	1	1	1	1	1	1	1	1	1	1	1	1	1	1	1	1	1	1	1	1	1
K08363	0	0	0	0	0	1	0	0	0	0	0	0	0	0	1	0	0	0	0	0	0	1	0	0	0	0
K08364	1	1	1	1	1	2	1	1	1	1	1	1	1	1	2	1	1	1	2	1	1	2	1	1	1	1
K08365	0	0	0	0	0	1	0	0	0	0	0	0	0	0	1	0	0	0	1	0	0	1	0	0	0	0
K08679	0	0	0	0	1	1	0	0	0	0	0	0	1	0	0	1	1	1	0	1	1	0	0	0	0	1
K08688	0	0	0	0	0	0	0	0	0	0	0	0	0	0	0	0	0	0	0	0	0	1	1	0	0	0
K08738	1	1	1	1	1	1	1	1	1	1	1	1	1	1	1	1	1	1	1	1	1	1	1	1	1	1
K08884	1	1	1	1	1	1	1	1	1	1	0	1	1	1	1	1	1	1	1	1	1	1	1	1	1	1
K08973	1	1	1	1	1	1	1	1	1	1	1	1	1	1	1	1	1	1	1	1	1	1	1	1	1	1
K08974	1	1	1	1	1	1	1	1	1	1	1	1	1	1	1	1	1	1	1	1	1	1	1	1	1	1
K08984	1	0	0	1	1	0	1	1	1	0	0	0	0	0	0	0	0	1	1	1	0	0	0	1	1	1
K08989	0	0	0	1	0	0	1	1	0	0	0	0	0	0	0	0	0	0	0	0	0	0	0	0	0	0
K08992	1	1	1	1	1	1	1	1	1	1	1	1	1	1	1	1	1	1	1	1	1	1	1	1	1	1
K08994	1	1	1	0	1	1	0	1	1	1	1	1	1	2	1	1	1	1	1	1	1	1	1	1	1	2
K08997	1	1	1	1	1	1	1	1	1	1	1	1	1	1	1	1	1	1	1	1	1	1	1	1	1	1
K08998	0	1	1	1	1	0	1	1	1	0	0	0	1	1	1	1	1	1	1	1	1	1	1	1	1	0
K09000	0	0	0	1	0	0	0	0	0	0	0	0	0	1	0	0	0	0	0	0	0	0	0	0	0	0
K09001	1	1	1	1	1	1	1	1	1	1	1	1	1	1	1	1	1	1	1	1	1	1	1	1	1	1
K09005	0	0	0	1	0	0	0	0	0	0	0	0	0	0	0	0	0	0	0	0	0	0	0	0	0	0
K09007	0	0	0	0	0	0	0	0	0	0	0	0	0	0	1	0	0	0	0	0	0	0	0	0	0	0
K09017	0	1	1	0	1	2	0	0	0	1	2	1	1	1	1	1	2	1	1	0	0	1	1	0	0	0
K09018	0	0	0	0	0	1	0	0	0	0	1	0	0	0	0	0	1	0	0	0	0	0	0	0	0	0
K09019	0	0	0	0	0	1	0	0	0	0	1	0	0	0	0	0	1	0	0	0	0	0	0	0	0	0
K09020	0	0	0	0	0	1	0	0	0	0	1	0	0	0	0	0	1	0	0	0	0	0	0	0	0	0
K09021	0	0	0	0	0	1	0	0	0	0	1	0	0	0	0	0	1	0	0	0	0	0	0	0	0	0
K09022	0	0	0	0	0	0	0	0	0	0	0	0	0	0	0	0	0	0	0	1	0	0	0	0	0	0
K09023	0	0	0	0	0	1	0	0	0	0	0	0	0	0	0	0	1	0	0	0	0	0	0	0	0	0
K09024	1	1	1	1	1	2	1	1	1	1	2	1	1	1	1	1	2	1	1	1	1	1	1	1	1	1
K09117	1	1	1	1	1	1	1	1	1	1	1	1	1	1	1	1	1	1	1	1	1	1	1	1	1	1
K09124	0	0	0	0	0	0	1	0	0	0	0	0	0	0	0	0	0	1	1	0	0	0	0	0	0	0
K09125	0	0	0	0	0	0	0	0	0	0	0	0	0	0	0	0	0	0	0	0	0	1	1	0	0	0
K09127	0	0	0	1	0	0	0	0	0	0	0	0	0	1	0	0	0	0	0	0	0	0	0	0	0	0
K09157	0	0	0	0	0	0	0	0	1	0	0	0	0	0	0	0	0	0	0	1	0	0	0	1	1	0
K09158	1	1	1	1	1	1	1	1	1	1	1	1	1	1	1	1	1	1	1	1	1	1	1	1	1	1
K09159	1	1	1	1	1	1	1	1	1	1	1	1	1	1	1	1	1	1	1	1	1	1	1	1	1	1
K09160	1	1	1	1	1	1	1	1	1	1	1	1	1	1	1	1	1	1	1	1	1	1	1	1	1	1
K09161	0	0	0	0	0	0	0	0	0	0	0	0	0	0	0	0	0	1	0	0	0	0	0	0	0	0
K09165	0	0	1	0	0	0	0	0	0	1	0	0	0	0	0	0	1	0	0	1	0	0	0	0	0	0
K09384	0	0	0	0	0	0	0	0	0	0	0	0	0	0	0	0	0	1	0	0	0	0	0	0	0	0
K09456	0	0	0	0	0	0	0	0	1	0	1	0	0	0	0	0	1	0	0	1	0	1	1	0	1	0
K09458	1	1	1	1	1	1	1	1	1	1	1	1	1	1	1	1	1	1	1	1	1	1	1	1	1	1

K09471	0	0	0	1	1	0	0	0	0	1	1	0	1	1	0	1	0	1	1	1	0	0	0	0	0	0
K09472	0	0	1	1	0	0	0	1	1	1	1	0	0	1	0	1	1	1	1	0	0	0	0	1	1	1
K09473	0	0	0	1	1	0	0	0	0	1	0	0	1	1	0	1	0	1	1	1	0	0	0	0	0	0
K09483	0	0	0	0	1	0	0	0	0	0	0	0	0	0	0	0	0	0	0	0	0	0	0	0	0	0
K09688	0	0	0	0	1	0	0	0	1	0	0	0	0	0	0	1	1	1	1	1	0	0	0	1	1	1
K09689	0	0	0	0	1	0	0	0	1	0	0	1	1	1	0	1	1	1	1	1	0	0	0	1	1	1
K09701	1	1	1	0	1	0	0	0	0	0	1	0	1	1	0	1	1	1	1	0	0	0	0	0	0	0
K09710	1	1	1	1	1	1	1	1	1	1	1	1	1	1	1	1	1	1	1	1	1	1	1	1	1	1
K09747	1	1	1	1	1	1	1	1	1	1	1	1	1	1	1	1	1	1	1	1	1	1	1	1	1	1
K09748	1	1	1	1	1	1	1	1	1	1	1	1	1	1	1	1	1	1	1	1	1	1	1	1	1	1
K09761	1	1	1	1	1	1	1	1	1	1	1	1	1	1	1	1	1	1	1	1	1	1	1	1	1	1
K09764	1	1	1	1	1	1	1	1	1	1	1	0	0	0	1	0	1	1	1	1	1	1	1	1	1	1
K09765	0	0	0	0	0	0	0	0	0	0	0	0	0	1	0	0	0	0	0	0	0	0	0	0	0	0
K09768	1	1	1	1	1	1	1	1	1	1	1	0	1	1	1	1	1	1	1	1	1	1	1	1	1	1
K09771	1	1	2	0	1	1	0	0	1	2	1	1	0	1	1	1	2	1	1	1	0	2	2	1	1	1
K09773	1	1	1	1	1	1	1	1	1	1	1	0	1	1	1	1	1	1	1	1	1	1	1	1	1	1
K09774	1	1	1	1	1	1	1	1	1	1	1	1	1	1	1	1	1	1	1	1	1	1	1	1	1	1
K09780	1	1	1	1	1	1	1	1	1	1	1	1	1	1	1	1	1	1	1	1	1	1	1	1	1	1
K09781	1	1	2	1	1	1	1	1	1	1	1	1	1	1	1	1	1	1	1	1	1	1	1	1	1	1
K09788	1	1	1	1	1	1	1	1	1	1	1	1	1	1	1	1	1	1	1	1	1	1	1	1	1	1
K09790	1	1	1	1	1	1	2	1	1	1	1	1	1	1	1	1	1	1	1	0	1	1	1	1	1	1
K09792	1	1	1	1	1	1	1	1	1	1	1	1	2	1	1	2	1	1	1	1	2	1	1	1	1	1
K09794	1	1	1	1	1	1	1	1	2	1	1	1	1	1	1	1	1	1	1	2	1	1	1	2	2	1
K09799	1	1	3	1	1	1	1	2	1	2	1	1	1	1	1	1	1	1	1	1	1	1	1	1	1	1
K09800	1	1	1	1	1	1	1	1	1	1	1	1	1	1	1	1	1	1	1	1	1	1	1	1	1	1
K09801	1	1	1	1	1	1	1	1	1	1	0	0	1	1	1	1	1	1	1	1	1	1	1	1	1	1
K09803	0	0	1	0	1	0	0	0	1	0	0	0	0	1	0	0	0	1	2	0	0	0	0	1	1	1
K09805	0	0	0	0	0	0	1	0	0	0	0	0	0	0	0	0	0	0	0	0	1	0	0	0	0	0
K09808	1	1	1	2	2	1	1	2	1	1	1	1	1	2	2	2	1	2	2	1	1	1	1	1	1	1
K09810	1	1	1	1	2	1	1	1	1	1	1	1	2	2	2	2	1	2	2	1	1	1	1	1	1	1
K09815	1	1	1	1	1	1	1	1	1	1	1	1	1	1	1	1	1	1	1	1	1	1	1	1	1	1
K09816	1	1	1	1	1	1	1	1	1	1	1	1	1	1	1	1	1	1	1	1	1	1	1	1	1	1
K09817	1	1	1	1	1	1	1	1	1	1	1	1	0	1	1	1	1	1	1	1	1	1	1	1	1	1
K09823	1	1	1	1	1	1	1	1	1	1	1	1	1	1	1	1	1	1	1	1	1	1	1	1	1	1
K09858	1	1	1	0	1	1	1	1	1	1	1	1	1	1	1	1	1	1	1	1	1	1	1	1	1	1
K09861	1	1	1	1	1	1	1	1	1	1	1	1	1	1	1	1	1	1	1	1	1	1	1	1	1	1
K09862	1	1	1	1	1	1	0	1	1	1	1	1	1	1	1	1	1	0	0	1	1	1	1	1	1	0
K09888	1	1	1	0	1	1	1	1	1	1	1	1	1	1	1	1	1	1	1	1	1	1	1	1	1	1
K09889	1	1	1	1	1	1	1	1	1	1	1	1	1	1	1	1	1	1	1	1	1	1	1	1	1	1
K09895	1	1	1	1	1	1	1	1	1	1	1	1	1	1	1	1	1	1	1	1	1	1	1	1	1	1
K09902	1	1	1	1	1	1	1	1	1	1	1	1	1	1	1	1	1	1	1	1	1	1	1	1	1	1
K09903	1	1	1	1	1	1	1	1	1	1	1	1	1	1	1	1	1	1	1	1	1	1	1	1	1	1
K09906	1	1	1	1	1	1	1	1	1	1	0	0	1	1	1	1	1	1	1	1	1	1	1	1	1	1
K09909	1	1	1	1	1	1	1	1	1	1	1	1	1	1	1	1	1	1	1	1	1	1	1	1	1	1
K09912	1	1	1	1	1	1	1	1	1	1	1	1	1	1	1	1	1	1	1	1	1	1	1	1	1	1
K09913	2	2	2	1	2	2	2	2	2	2	2	2	2	2	2	2	2	2	2	2	2	2	2	2	2	2
K09916	1	1	1	1	1	1	1	1	1	1	1	1	1	1	1	1	1	1	1	1	1	1	1	1	1	1
K09919	1	1	1	1	1	1	1	1	1	1	1	1	1	1	1	1	1	1	1	1	1	1	1	0	1	1
K09924	0	0	1	2	0	0	0	0	1	0	0	0	1	1	0	0	0	0	0	0	1	0	0	1	1	0
K09926	1	1	1	1	1	1	1	1	1	1	1	1	1	1	1	1	1	1	1	1	1	1	1	1	1	1
K09928	1	1	1	0	1	1	0	0	1	1	1	1	1	1	1	1	1	1	1	1	0	1	1	1	1	1
K09929	0	0	0	1	0	0	0	0	0	0	0	0	0	0	0	0	0	0	0	0	0	0	0	0	0	0
K09930	0	0	0	1	0	0	0	0	0	0	0	0	0	0	0	0	0	0	0	0	0	0	0	0	0	0
K09931	1	1	0	0	1	0	0	0	1	1	1	1	1	1	0	1	1	1	1	1	0	1	1	1	1	0
K09933	1	1	1	1	1	1	0	1	1	1	1	1	1	1	2	1	1	1	1	1	1	1	1	1	1	1
K09935	0	0	0	0	0	1	0	0	0	0	0	0	0	0	0	0	0	0	0	0	0	0	0	0	0	0
K09936	2	2	2	0	3	0	0	1	0	2	0	0	0	0	0	1	0	0	0	1	0	0	0	0	0	5
K09947	1	1	1	1	1	1	1	1	1	1	1	1	1	1	1	1	1	1	1	1	1	1	1	1	1	1
K09948	1	0	1	0	0	1	0	0	1	1	1	1	0	0	0	0	1	1	1	1	0	1	1	1	1	1
K09951	0	0	0	2	0	1	1	0	0	0	0	0	0	0	1	0	0	0	0	0	0	0	0	0	0	0
K09956	0	0	0	0	0	0	0	0	0	0	0	0	0	1	0	0	0	0	0	0	0	0	0	0	0	0
K09959	1	1	1	0	1	1	1	0	1	1	1	1	1	1	1	1	1	1	1	1	1	1	1	1	1	2
K09960	0	0	0	0	0	0	0	0	0	0	0	0	0	0	0	0	0	0	0	0	0	0	0	0	0	1
K09961	0	0	0	0	0	0	0	0	0	0	0	0	0	0	0	0	0	0	0	0	0	0	0	0	0	1
K09964	0	0	0	0	0	0	0	0	0	0	0	0	0	0	0	0	0	0	0	1	0	0	0	0	0	0
K09966	1	1	1	1	1	1	1	1	1	1	1	1	1	1	1	1	1	1	1	1	1	1	1	1	1	1
K09967	2	0	0	0	0	0	0	0	0	0	0	0	0	0	0	0	0	0	0	0	0	0	0	0	0	0
K09974	0	0	0	0	1	0	0	0	1	0	0	0	1	0	0	1	0	1	1	1	0	0	0	1	1	1

K09977	1	1	1	1	1	1	1	1	1	1	1	1	1	1	1	1	1	1	1	1	1	1	1	1	1	1
K09984	0	0	0	0	0	0	0	0	0	0	0	0	0	0	1	0	0	0	0	1	0	0	0	0	0	0
K09986	1	1	1	1	1	1	1	1	1	1	0	1	1	1	1	1	1	1	1	1	1	1	1	1	1	1
K09989	1	1	1	1	1	1	1	1	1	1	1	1	1	1	1	1	1	1	1	1	1	1	1	1	1	1
K10001	1	1	1	0	1	1	0	0	1	1	1	1	1	1	1	1	1	1	1	1	1	1	1	1	1	1
K10002	1	1	1	0	1	1	0	0	1	1	1	1	1	0	1	1	1	1	1	1	1	1	1	1	1	1
K10003	1	1	1	0	1	1	0	0	1	1	1	1	1	0	1	1	1	1	1	1	1	1	1	1	1	1
K10004	1	1	1	0	1	1	0	0	1	1	1	1	1	1	1	1	1	1	1	1	1	1	1	0	1	1
K10009	1	0	1	1	1	1	0	1	1	1	1	1	1	1	1	1	1	1	1	1	1	1	1	1	1	1
K10010	1	0	1	1	1	1	0	1	1	1	1	1	1	1	1	1	1	1	1	1	1	1	1	1	1	1
K10026	1	1	1	1	1	1	1	1	1	1	1	1	1	1	1	1	1	1	1	1	1	0	0	1	1	1
K10107	0	0	0	0	1	0	0	0	1	0	1	1	1	1	0	1	1	1	1	1	0	0	0	1	1	1
K10216	0	0	0	0	1	0	1	1	0	0	0	0	0	0	0	0	0	0	0	0	1	0	0	0	0	0
K10254	0	0	0	0	2	0	0	0	0	0	0	0	1	1	0	1	0	0	0	0	0	0	0	0	0	0
K10536	0	0	1	0	1	0	0	0	0	1	1	0	1	1	0	1	0	1	1	1	0	0	0	0	0	0
K10563	1	1	1	1	1	1	1	1	1	1	1	1	1	1	1	1	1	1	1	1	1	1	1	1	1	1
K10678	0	0	0	1	0	0	1	1	0	0	0	0	0	0	0	0	0	0	0	0	1	0	0	0	0	0
K10679	0	0	0	0	1	0	1	1	1	0	1	0	1	1	0	1	2	1	1	0	0	0	0	1	1	0
K10680	2	1	2	1	2	1	1	1	2	2	2	2	1	2	2	2	2	2	2	2	1	1	1	2	2	1
K10716	2	2	2	2	2	2	2	2	2	2	2	2	2	2	2	2	2	2	2	2	2	2	2	2	2	2
K10763	1	1	1	1	1	1	1	1	1	1	1	1	1	1	1	1	1	1	1	1	1	1	1	1	1	1
K10764	1	1	1	1	1	1	1	1	1	1	1	1	1	1	1	1	1	1	1	1	1	1	1	1	1	1
K10773	1	1	1	1	1	1	1	1	1	1	1	1	1	1	1	1	1	1	1	3	1	1	1	1	1	1
K10804	1	1	1	1	1	1	1	1	1	1	1	1	1	1	1	1	1	1	1	1	1	1	1	1	1	1
K10805	1	1	1	1	1	1	1	1	1	1	1	1	1	1	1	1	1	1	1	1	1	1	1	1	1	1
K10823	0	0	0	1	0	0	1	1	0	0	1	0	1	1	0	1	1	0	0	1	1	0	0	0	0	1
K10857	1	0	1	1	2	1	2	2	2	1	1	1	1	2	2	1	1	1	1	2	1	1	1	2	2	1
K10918	0	0	0	0	0	1	0	0	0	0	0	0	0	0	0	0	0	0	0	0	0	0	0	0	0	1
K10938	0	0	0	0	0	1	0	0	1	0	0	0	0	0	0	0	0	1	0	0	0	0	0	1	1	0
K11016	0	0	0	0	0	0	1	1	0	0	0	0	0	0	0	0	0	0	0	0	0	0	0	0	0	0
K11066	1	0	0	0	0	0	1	1	1	1	0	1	1	1	1	1	1	1	1	1	1	0	0	1	1	1
K11068	0	0	0	1	0	0	0	0	0	0	0	0	0	0	0	0	0	0	0	0	0	0	0	0	0	0
K11069	1	0	1	1	1	1	1	1	1	1	1	1	1	1	1	1	1	1	1	1	1	1	1	1	1	1
K11070	1	0	1	1	1	1	1	1	1	1	1	1	1	1	1	1	1	1	1	1	1	1	1	1	1	1
K11071	1	0	1	1	1	1	1	1	1	1	1	1	1	1	1	1	1	1	1	1	1	1	1	1	1	1
K11072	1	0	1	1	1	1	1	1	1	1	1	1	1	1	1	1	1	1	1	1	1	1	1	1	1	1
K11085	1	1	1	1	1	1	1	1	1	1	1	1	1	1	1	1	1	1	1	1	1	1	1	1	1	1
K11105	1	0	2	1	1	1	1	1	2	1	1	1	1	1	0	1	1	1	1	1	1	0	0	2	2	1
K11107	1	1	1	1	1	1	1	1	1	1	1	1	1	1	1	1	1	1	1	1	1	1	1	1	1	0
K11175	1	1	1	1	1	1	1	1	1	1	1	1	1	1	1	1	1	1	1	1	1	1	1	1	1	1
K11177	1	0	1	0	1	1	1	1	1	1	1	1	1	1	0	1	1	1	1	1	1	1	1	1	1	1
K11178	1	0	1	0	1	1	1	1	1	1	1	1	1	1	1	1	1	1	1	1	1	1	1	1	1	1
K11179	1	1	1	1	1	1	1	1	1	1	0	0	1	1	1	1	1	1	1	1	1	1	1	1	1	1
K11209	1	1	1	1	1	1	1	1	1	1	2	2	1	1	2	1	2	2	2	2	1	1	1	1	1	1
K11250	0	0	0	0	0	0	1	1	0	0	0	0	0	0	0	0	0	0	0	0	1	0	0	0	0	0
K11472	0	1	1	0	1	0	0	0	0	1	0	0	0	0	1	0	0	1	1	0	0	0	0	0	0	0
K11473	0	1	1	0	1	0	0	0	0	1	0	0	0	0	1	0	0	1	1	0	0	0	0	0	0	0
K11475	0	0	0	0	1	0	0	0	0	0	0	0	0	0	0	0	0	0	0	0	0	0	0	0	0	0
K11719	1	1	1	1	1	1	1	1	1	1	1	1	1	1	1	1	1	1	1	1	1	1	1	1	1	1
K11720	1	1	1	1	1	1	1	1	1	1	1	1	1	1	1	1	1	1	1	1	1	1	1	1	1	1
K11747	1	1	1	1	1	1	1	1	1	1	1	1	1	1	1	1	1	1	1	1	1	1	1	1	1	1
K11749	1	1	1	1	1	1	1	1	1	1	1	1	1	1	1	1	1	1	1	1	1	1	1	1	1	1
K11752	1	1	1	1	1	1	1	1	1	1	1	1	1	1	1	1	1	1	1	1	1	1	1	1	1	1
K11753	1	1	1	1	1	1	1	1	1	1	1	1	1	1	1	1	1	1	1	1	1	1	1	1	1	1
K11754	1	1	1	1	1	1	1	1	1	1	1	1	1	1	1	1	1	1	1	1	1	1	1	0	1	1
K11755	1	1	1	1	1	1	1	1	1	1	1	1	1	1	1	1	1	1	1	1	1	1	1	1	1	1
K11811	1	1	1	0	1	1	1	1	1	1	1	1	1	1	1	1	1	1	1	1	1	1	1	1	1	1
K11891	1	0	0	0	1	0	0	0	1	0	0	0	0	0	0	1	0	1	0	1	0	0	0	0	1	0
K11892	1	0	0	0	1	0	0	0	1	0	0	0	1	1	0	1	0	1	0	1	0	0	0	1	1	0
K11893	1	0	0	0	1	0	0	0	1	0	0	0	1	1	0	1	0	1	0	1	0	0	0	1	1	0
K11895	1	0	0	0	1	0	0	0	1	0	0	0	1	1	0	1	0	1	0	1	0	0	0	1	1	0
K11896	1	0	0	0	1	0	0	0	1	0	0	0	1	0	0	1	0	1	0	1	0	0	0	1	1	0
K11897	1	0	0	0	1	0	0	0	1	0	0	0	0	1	0	1	0	1	0	1	0	0	0	1	1	0
K11900	1	0	0	0	1	0	0	0	1	0	0	0	1	1	0	1	0	1	0	1	0	0	0	1	1	0
K11901	1	0	0	0	1	0	0	0	1	0	0	0	1	1	0	1	0	1	0	1	0	0	0	1	1	0
K11902	1	0	0	0	1	0	0	0	1	0	0	0	1	1	0	1	0	1	0	1	0	0	0	1	1	0
K11903	1	0	0	0	1	0	0	0	1	0	0	0	1	1	0	1	0	1	0	1	0	0	0	1	1	0
K11907	1	0	0	0	1	0	0	0	1	0	0	0	1	0	0	1	0	1	0	1	0	0	0	1	1	0

K11921	0	0	0	0	1	0	0	0	0	1	0	0	1	0	0	1	0	0	0	0	0	0	0	0	0	0
K11923	0	0	0	0	0	1	0	0	0	0	0	0	0	0	0	0	0	0	0	0	0	0	0	0	0	0
K11928	0	0	0	0	0	0	1	0	0	0	0	0	0	0	0	0	0	0	0	0	0	0	0	0	0	0
K11939	0	0	0	0	0	0	0	0	0	0	0	0	0	0	0	0	0	0	0	0	0	0	0	0	0	1
K11959	1	0	1	1	1	1	0	0	1	1	1	1	1	1	0	1	1	0	0	1	0	1	1	1	1	0
K11960	1	0	1	1	1	1	0	0	1	1	1	1	1	1	0	1	1	0	0	1	0	1	1	1	1	0
K11961	1	0	1	1	1	1	0	0	1	1	1	1	1	1	0	1	1	0	0	1	0	1	1	1	1	0
K11962	1	0	1	1	1	1	0	0	1	1	0	0	1	1	0	1	1	0	0	1	0	1	1	1	1	0
K11963	1	0	1	1	1	1	0	0	1	1	1	1	1	1	0	1	1	0	0	1	0	1	1	1	1	0
K11991	0	0	1	0	0	0	0	0	1	1	0	0	1	1	0	0	0	0	0	0	0	1	1	1	1	1
K11996	1	1	1	1	1	1	1	1	1	1	1	1	1	1	1	1	1	1	1	1	1	1	1	1	1	1
K12251	0	0	0	0	1	0	0	0	0	1	1	0	1	1	0	1	0	1	1	1	0	0	0	0	0	0
K12255	1	0	1	0	1	1	0	0	1	1	1	1	1	1	1	1	1	1	1	1	0	0	0	1	1	1
K12262	1	1	1	1	1	1	1	1	1	1	1	1	1	2	1	1	1	1	2	1	2	1	1	1	1	1
K12267	2	1	2	1	2	1	1	1	2	2	1	1	2	1	1	2	1	1	1	1	1	1	1	2	2	1
K12297	1	1	1	1	1	1	1	1	1	1	1	1	1	1	1	1	1	1	1	1	1	1	1	1	1	1
K12339	1	1	1	1	1	1	1	1	1	1	1	1	1	1	1	1	1	1	1	1	1	1	1	1	1	1
K12349	0	0	0	1	0	0	1	1	1	0	0	0	0	0	0	0	0	0	0	0	1	0	0	0	1	0
K12368	0	0	1	1	1	1	0	1	1	1	1	1	1	1	0	1	1	1	1	1	1	0	0	1	1	1
K12369	0	0	1	1	1	1	0	1	1	1	1	1	1	1	0	1	1	1	1	1	1	0	0	1	1	1
K12370	0	0	1	1	1	1	0	1	1	1	1	1	1	1	0	1	1	1	1	1	1	0	0	1	1	1
K12371	0	0	1	0	1	1	0	1	1	1	1	1	1	1	0	1	1	1	1	1	1	0	0	1	1	1
K12372	0	0	1	1	1	1	0	1	1	1	1	1	1	1	0	1	1	1	1	1	1	0	0	1	1	1
K12410	1	0	1	1	1	1	1	1	1	1	1	1	1	1	0	1	1	1	1	1	1	1	1	1	1	1
K12508	0	0	0	0	1	0	0	0	0	0	0	0	0	0	0	1	0	0	0	0	0	0	0	0	0	0
K12510	0	0	0	1	0	0	0	0	0	0	0	0	0	0	0	0	0	0	0	0	0	0	0	0	0	0
K12511	0	0	0	1	0	0	0	0	0	0	0	0	0	0	0	0	0	0	0	0	0	0	0	0	0	0
K12541	0	1	0	1	1	0	1	1	0	1	1	0	0	1	0	2	1	0	0	1	1	1	1	0	0	0
K12542	0	1	0	1	1	0	1	1	0	1	1	0	1	1	0	2	1	0	0	1	1	1	1	0	0	0
K12543	0	1	0	1	1	0	1	1	0	1	1	0	1	1	0	2	1	0	0	1	1	1	1	0	0	0
K12573	1	1	1	1	1	1	1	1	1	1	1	1	1	1	1	1	1	1	1	1	1	1	1	1	1	1
K12658	0	0	1	1	0	0	0	0	1	1	1	0	0	1	0	1	1	1	1	0	0	0	0	1	1	1
K12686	0	0	1	1	0	1	1	1	0	1	0	1	0	0	0	0	1	0	0	0	1	0	0	0	0	0
K12942	0	0	0	0	0	0	1	0	0	0	1	0	0	0	0	0	0	0	0	0	1	1	1	0	0	1
K12957	1	0	1	0	0	0	0	1	0	1	1	1	0	0	0	0	1	0	0	1	0	0	0	0	0	1
K12981	1	0	0	0	0	0	0	0	0	0	1	0	0	0	1	0	1	1	0	1	0	0	0	0	0	1
K12988	0	0	0	1	0	0	0	0	0	1	0	0	0	0	0	0	0	0	0	0	0	0	0	0	0	0
K13016	0	0	1	1	0	0	0	0	0	0	0	0	0	0	0	0	0	0	0	0	1	0	0	0	0	0
K13017	0	0	1	1	0	0	0	0	0	0	0	0	0	0	0	0	0	0	0	0	1	0	0	0	0	0
K13018	0	0	0	1	0	0	0	0	0	0	0	0	0	0	0	0	0	0	0	0	1	0	0	0	0	0
K13038	1	1	1	1	1	1	1	1	1	1	1	1	1	1	1	1	1	1	1	1	1	1	1	1	1	1
K13040	0	1	0	0	1	0	0	0	0	0	0	0	1	1	1	1	0	0	0	0	0	0	0	0	0	0
K13041	0	1	0	0	1	0	0	0	0	0	0	0	1	1	1	1	0	0	0	0	0	0	0	0	0	0
K13288	1	1	1	1	1	1	1	1	1	1	1	1	1	1	1	1	1	1	1	1	1	1	1	1	1	1
K13292	1	1	2	1	1	1	1	1	1	2	1	1	2	2	1	2	1	2	2	1	2	1	1	1	1	1
K13378	1	1	1	1	1	1	1	1	1	1	1	1	1	1	1	1	1	1	1	1	1	1	1	1	1	1
K13481	0	0	1	0	0	1	0	0	0	1	1	1	0	0	1	0	1	1	1	0	0	1	1	0	0	0
K13482	0	0	1	0	0	1	0	0	0	1	1	1	0	0	1	0	1	1	1	0	0	1	1	0	0	0
K13483	1	0	1	0	1	1	1	1	1	1	0	1	1	1	0	1	1	1	1	1	1	1	1	1	1	1
K13500	0	0	0	0	0	0	0	0	0	0	0	1	0	0	0	0	0	0	0	0	0	0	0	0	0	0
K13522	1	1	1	1	1	1	1	1	1	1	1	1	1	1	1	1	1	1	1	1	1	1	1	1	1	1
K13628	1	1	1	1	1	1	1	1	1	1	1	1	1	1	1	1	1	1	1	1	1	1	1	1	1	1
K13635	1	1	1	1	1	1	1	1	1	1	1	1	1	1	1	1	1	1	1	1	1	1	1	1	1	1
K13636	1	0	0	0	0	0	0	0	0	0	0	0	0	0	0	0	0	0	0	0	0	0	0	0	0	0
K13639	1	0	0	0	0	0	0	0	0	0	0	0	0	0	0	0	0	0	0	0	0	0	0	0	0	1
K13643	1	1	1	1	1	1	1	1	1	1	1	1	1	1	1	1	1	1	1	1	1	1	1	1	1	1
K13693	0	0	1	0	0	0	0	0	0	0	0	0	0	0	0	0	0	0	0	0	0	0	0	0	0	0
K13745	1	0	0	0	1	0	1	0	0	0	1	0	0	0	0	0	0	0	0	1	0	0	0	0	0	1
K13747	1	1	1	1	1	1	1	1	1	1	1	1	1	1	1	1	1	1	1	1	1	1	1	1	1	1
K13766	1	1	1	1	1	1	1	1	1	1	1	1	1	1	1	1	1	1	1	1	1	1	1	1	1	1
K13767	0	0	0	0	0	0	0	0	0	0	0	0	0	0	0	0	0	1	1	0	0	0	0	0	0	0
K13771	1	0	1	1	1	0	1	1	1	1	1	1	1	1	1	1	1	1	1	1	1	1	1	1	1	1
K13774	0	0	0	0	0	0	0	0	0	1	0	0	0	0	0	0	0	1	0	0	0	0	0	0	0	0
K13788	1	1	1	1	1	1	1	1	1	1	1	0	1	1	1	1	1	1	1	1	1	1	1	0	1	1
K13821	1	1	1	0	1	1	1	1	1	1	1	1	1	1	1	1	1	1	1	1	1	1	1	1	1	1
K13874	0	0	0	0	0	0	0	0	0	0	0	0	0	0	0	0	0	0	0	0	0	0	0	0	0	1
K13888	1	1	1	1	1	1	1	1	1	1	1	1	1	1	1	1	1	1	1	1	1	1	1	1	1	1
K13893	0	0	0	0	0	0	0	0	0	1	0	0	0	0	0	0	0	0	0	0	0	0	0	0	0	0

K13894	1	1	1	1	1	1	1	1	1	1	1	1	1	1	1	1	1	1	1	1	1	1	1	1	1	1
K13895	1	1	1	1	1	1	1	1	1	1	0	1	1	1	1	1	1	1	1	1	1	1	1	1	1	1
K13896	1	1	1	0	1	1	1	1	1	1	1	0	0	1	1	1	1	1	1	1	1	1	1	1	1	1
K13926	0	0	0	1	0	0	1	0	0	0	0	0	0	0	0	0	0	0	0	1	0	0	0	0	0	0
K13929	1	1	0	0	1	0	0	0	0	1	0	0	0	0	1	0	0	0	1	0	0	0	0	0	0	1
K13930	1	1	0	0	1	0	0	0	0	1	0	0	0	0	1	0	0	0	1	0	0	0	0	0	0	1
K13931	1	1	0	0	1	0	0	0	0	1	0	0	0	0	1	0	0	0	1	0	0	0	0	0	0	1
K13932	1	1	0	0	1	0	0	0	0	1	0	0	0	0	1	0	0	0	1	0	0	0	0	0	0	1
K13933	1	1	0	0	1	0	0	0	0	1	0	0	0	0	1	0	0	0	1	0	0	0	0	0	0	1
K13934	1	1	0	0	1	0	0	0	0	1	0	0	0	0	1	0	0	0	1	0	0	0	0	0	0	1
K13953	1	0	0	0	1	1	1	0	1	0	0	1	1	1	1	1	0	1	1	0	0	1	1	1	1	1
K13954	0	0	0	1	0	0	0	0	0	0	0	0	0	0	0	0	0	0	0	0	1	0	0	0	0	0
K13963	0	0	0	0	0	0	0	0	0	0	0	0	0	0	0	0	0	0	0	1	0	0	0	0	0	0
K13993	0	0	0	1	0	0	1	1	0	0	0	0	0	0	0	0	0	1	1	0	0	0	0	0	0	0
K14056	0	0	0	1	1	0	0	0	0	1	0	0	1	1	0	1	0	1	1	1	0	0	0	0	0	0
K14058	1	1	1	1	1	1	1	1	1	1	1	1	1	1	1	1	1	1	1	1	1	1	1	1	1	1
K14059	3	1	1	0	0	3	3	0	0	3	0	2	2	2	0	2	1	2	1	0	2	2	2	0	0	1
K14155	1	0	1	1	1	1	1	1	1	1	1	1	1	1	0	1	1	1	1	1	1	1	1	1	1	1
K14159	1	1	1	1	1	1	1	1	1	1	1	1	1	1	1	1	1	1	1	2	0	1	1	1	1	1
K14170	1	1	1	1	1	1	1	1	1	1	1	1	1	1	1	1	1	1	1	1	1	1	1	1	1	1
K14260	1	1	1	1	1	1	1	1	1	1	1	1	1	1	1	1	1	1	1	1	1	1	1	1	1	1
K14267	1	1	1	1	1	1	1	1	1	1	1	1	1	1	1	1	1	1	1	1	1	1	1	1	1	1
K14392	0	0	0	0	0	0	0	0	0	0	0	0	0	0	0	0	0	0	0	0	0	1	1	0	0	0
K14393	1	1	1	1	1	1	1	1	1	1	1	1	1	1	1	1	1	1	1	1	1	1	1	0	1	1
K14414	1	0	0	0	1	1	0	0	0	0	0	0	0	1	0	1	0	1	0	0	0	0	0	0	0	1
K14415	2	0	0	0	2	2	1	1	0	0	1	0	2	1	0	2	1	1	0	0	1	0	0	0	0	1
K14424	0	0	0	0	0	0	0	0	1	0	0	0	0	0	0	0	0	0	0	0	0	0	0	1	1	0
K14441	1	1	1	1	1	1	1	1	1	1	1	1	1	1	1	1	1	1	1	1	1	1	1	1	1	1
K14445	1	1	1	1	1	1	1	1	1	1	1	1	1	1	1	1	1	1	1	1	1	1	1	1	1	1
K14519	0	0	0	0	0	0	0	0	0	0	0	0	0	0	0	0	0	0	0	0	0	0	0	0	0	1
K14540	1	1	1	1	1	1	1	1	1	1	1	1	1	1	1	1	1	1	1	1	1	1	1	1	1	1
K14623	0	0	0	1	0	0	0	0	0	0	0	0	0	0	0	0	0	0	0	0	0	0	0	0	0	0
K14652	1	1	1	1	1	1	1	1	1	1	1	1	1	1	1	1	1	1	1	1	1	1	1	1	1	1
K14682	1	1	1	1	1	1	1	1	1	1	1	1	1	1	1	1	1	1	1	1	1	1	1	1	1	1
K14683	0	0	0	1	0	0	1	0	0	0	0	0	0	0	0	0	0	0	0	0	0	0	0	0	0	0
K14727	0	0	0	1	1	0	0	0	0	0	1	0	1	0	1	0	0	1	1	0	0	0	0	0	0	0
K14742	1	1	1	1	1	1	1	1	1	1	1	1	1	1	1	1	1	1	1	1	1	1	1	1	1	1
K14748	0	0	0	0	0	0	0	0	0	0	0	0	0	0	0	1	0	0	0	0	0	0	0	0	0	0
K14749	0	0	0	0	0	0	0	0	0	0	0	0	0	0	0	1	0	0	0	0	0	0	0	0	0	0
K14977	0	0	1	0	1	0	0	0	0	1	0	0	1	1	0	1	0	1	1	0	0	1	1	0	0	0
K15034	1	1	1	0	1	1	1	1	1	1	0	1	1	1	1	1	1	1	1	1	1	1	1	0	1	1
K15045	0	0	0	1	0	0	0	0	0	0	0	0	0	0	0	0	0	1	0	0	0	0	0	0	0	0
K15125	0	0	0	0	0	0	2	0	0	0	0	0	0	0	0	0	0	0	0	1	0	0	0	0	0	0
K15256	1	1	1	1	1	1	1	1	1	1	1	1	1	1	1	1	1	1	1	1	1	1	1	1	1	1
K15257	1	1	1	1	1	1	1	1	1	1	1	1	1	1	1	1	1	1	1	1	1	1	1	1	1	1
K15268	0	1	1	0	2	1	1	1	1	1	1	1	1	1	1	1	1	0	0	1	0	0	0	1	1	1
K15269	0	0	0	0	0	0	1	0	0	0	0	0	0	0	0	0	0	0	0	0	0	0	0	0	0	0
K15270	0	0	0	0	1	0	0	0	0	0	1	1	0	0	0	0	0	1	0	0	2	0	0	0	0	1
K15342	0	0	0	2	0	1	1	0	0	1	0	0	0	0	2	1	0	0	0	1	0	0	0	0	0	0
K15371	1	1	1	0	1	1	0	1	1	0	1	1	1	1	1	1	1	1	1	1	1	1	1	1	1	1
K15396	1	1	1	1	1	1	1	1	1	1	1	1	0	1	1	1	1	1	1	1	1	1	1	1	1	1
K15461	1	1	1	1	1	1	1	1	1	1	1	1	1	1	1	1	1	1	1	1	1	1	1	1	1	1
K15539	0	0	0	0	0	0	0	1	0	0	0	0	0	0	0	0	0	0	0	0	0	0	0	0	0	0
K15580	0	0	0	2	0	0	2	2	0	0	1	0	1	1	0	1	1	0	0	1	3	0	0	0	0	1
K15581	0	0	0	1	0	0	1	1	0	0	1	0	1	1	0	1	1	0	0	1	1	0	0	0	0	1
K15582	0	0	0	1	0	0	1	1	0	0	1	0	1	1	0	1	1	0	0	1	1	0	0	0	0	1
K15583	0	0	0	1	0	0	1	1	0	0	1	0	1	1	0	1	1	0	0	1	1	0	0	0	0	1
K15633	1	1	1	1	1	1	1	1	1	1	1	1	1	1	1	1	1	1	1	1	1	1	1	1	1	1
K15724	1	1	1	1	1	1	1	1	1	1	1	1	1	1	1	1	1	1	1	1	1	1	1	1	1	1
K15725	0	0	0	0	0	1	0	0	1	0	0	0	0	0	0	0	0	1	0	0	0	1	1	1	1	0
K15726	0	0	0	0	0	1	0	0	1	0	0	0	0	0	0	0	0	1	0	0	0	1	1	1	1	0
K15727	0	0	0	0	0	1	0	0	1	0	0	0	0	0	0	0	0	1	0	0	0	1	1	1	1	0
K15738	1	1	1	1	1	1	1	1	1	1	1	1	1	1	1	1	1	1	1	1	1	1	1	1	1	1
K15778	1	1	1	0	1	1	1	1	1	1	0	1	1	1	1	1	1	1	1	1	1	1	1	0	1	1
K15866	1	1	1	2	1	1	1	1	1	1	2	1	2	1	2	1	1	1	1	1	1	1	1	1	1	1
K15896	0	1	0	0	0	0	0	0	0	0	0	0	0	0	0	0	0	0	0	0	0	0	0	0	0	0
K15977	2	1	2	2	1	1	1	1	1	2	2	1	1	1	1	1	2	1	1	1	2	1	1	1	1	2
K15984	1	1	1	1	1	1	1	1	1	1	1	1	1	1	1	1	1	1	1	1	1	1	1	1	1	1

K16012	0	0	0	1	0	0	1	1	0	0	0	0	0	0	0	0	0	0	0	0	1	0	0	0	0	0
K16013	0	0	0	1	0	0	1	1	0	0	0	0	0	0	0	0	0	0	0	0	1	0	0	0	0	0
K16049	0	0	0	0	0	0	0	0	0	0	0	0	0	0	0	1	0	0	0	0	0	0	0	0	0	0
K16052	1	1	1	1	1	1	1	1	1	1	1	1	1	1	1	1	1	1	1	1	1	1	1	1	1	1
K16066	1	0	1	0	0	1	0	0	0	1	1	1	0	0	1	1	0	0	0	0	0	0	0	0	0	0
K16087	1	0	1	1	1	1	3	1	1	1	1	1	1	3	0	1	1	1	1	1	1	1	1	1	1	0
K16088	1	0	0	0	0	0	0	0	0	0	0	0	0	0	0	0	0	0	0	0	0	0	0	0	0	1
K16089	1	0	0	1	1	0	1	0	0	1	0	1	0	1	0	2	1	0	0	1	2	1	1	0	0	1
K16090	0	0	0	0	0	0	0	0	0	0	0	0	0	0	0	0	0	0	0	0	1	0	0	0	0	0
K16091	0	0	1	0	0	0	1	0	0	1	0	0	0	0	1	0	0	0	0	0	0	0	0	0	0	0
K16092	1	1	1	1	1	1	1	1	1	1	1	1	1	1	1	1	1	1	1	1	1	1	1	1	1	1
K16137	1	1	1	1	1	1	1	1	1	1	2	1	1	1	1	1	2	1	1	1	1	1	1	1	1	1
K16165	0	0	0	1	0	0	0	0	0	0	0	0	0	0	0	0	0	0	0	0	2	0	0	0	0	0
K16242	0	0	0	0	0	0	0	0	0	0	0	0	0	0	0	1	0	1	1	0	0	0	0	0	0	0
K16243	0	0	0	0	0	0	0	0	0	0	0	0	0	0	0	1	0	1	1	0	0	0	0	0	0	0
K16244	0	0	0	0	0	0	0	0	0	0	0	0	0	0	0	1	0	1	1	0	0	0	0	0	0	0
K16245	0	0	0	0	0	0	0	0	0	0	0	0	0	0	0	1	0	1	1	0	0	0	0	0	0	0
K16246	0	0	0	0	0	0	0	0	0	0	0	0	0	0	0	1	0	1	1	0	0	0	0	0	0	0
K16249	0	0	0	0	0	0	0	0	0	0	0	0	0	0	0	1	0	1	1	0	0	0	0	0	0	0
K16263	0	0	0	1	0	0	0	0	0	0	0	0	0	0	0	0	0	0	0	0	0	0	0	0	0	0
K16264	1	0	1	1	2	2	1	2	2	1	2	1	2	1	1	2	2	2	1	1	1	2	1	2	2	2
K16345	0	0	1	0	1	1	0	0	0	1	1	1	1	1	0	1	1	2	2	0	0	1	1	0	0	0
K16422	0	0	0	1	0	0	0	0	0	0	0	0	0	0	0	0	0	0	0	0	1	0	0	0	0	0
K16692	1	1	1	1	1	1	1	1	0	1	1	1	1	1	1	1	1	1	1	0	1	1	1	0	0	1
K16710	0	0	0	0	0	1	0	0	0	0	0	0	0	0	0	0	0	0	0	0	0	0	0	0	0	0
K16840	0	0	1	0	1	1	0	0	0	1	1	1	1	1	1	1	1	1	1	0	0	1	1	0	0	0
K16841	0	0	1	0	0	0	0	0	0	1	0	0	0	0	0	0	0	1	1	0	0	0	0	0	0	0
K16842	0	0	1	1	1	1	1	0	0	1	1	1	1	1	1	1	1	1	1	0	0	1	1	0	0	0
K16872	1	1	1	1	1	1	1	1	1	1	1	1	1	1	1	1	1	1	1	1	1	1	1	1	1	1
K17103	1	1	1	1	1	1	1	1	1	1	1	1	1	1	1	1	1	1	1	1	1	1	1	1	1	1
K17218	0	0	0	0	0	0	0	0	0	0	0	0	0	0	1	0	0	0	0	0	0	0	0	0	0	0
K17677	0	0	0	0	0	0	0	0	0	0	0	0	0	0	0	0	0	1	0	0	1	0	0	0	0	0
K17686	2	2	2	1	1	1	1	3	2	3	2	1	3	2	2	3	3	3	2	2	2	4	4	2	2	3
K17713	1	1	1	1	1	1	1	1	1	1	1	1	1	1	1	1	1	1	1	1	1	1	1	1	1	1
K17716	0	0	0	1	0	0	0	0	0	0	0	0	0	0	0	0	0	0	0	0	0	0	0	0	0	0
K17736	0	0	0	0	0	0	0	0	0	1	0	0	0	0	0	0	0	1	0	0	0	0	0	0	0	0
K17865	0	0	0	1	0	0	0	0	0	0	0	0	0	0	0	0	0	0	0	0	0	0	0	0	0	0
K18009	1	1	1	1	1	1	1	1	1	1	1	1	1	1	1	1	1	1	1	1	1	1	1	1	1	1
K18092	0	0	0	0	0	0	0	0	0	0	0	0	0	0	0	1	0	0	0	0	0	0	0	0	0	0
K18118	0	1	1	0	0	0	0	0	0	1	0	0	0	0	0	0	0	1	1	0	0	1	1	0	0	0
K18138	1	1	1	1	1	1	1	1	1	1	1	1	1	1	1	1	1	1	1	1	1	1	1	1	1	1
K18139	1	1	1	1	1	1	1	1	1	1	1	1	1	1	2	1	1	1	1	1	1	1	1	1	1	1
K18277	0	0	0	0	0	0	1	0	1	0	0	0	0	1	2	1	0	1	1	0	0	1	1	1	1	0
K18292	1	0	0	0	1	1	0	0	1	1	1	1	1	0	1	1	1	1	1	0	0	0	0	1	1	1
K18333	0	0	0	0	0	0	0	0	0	0	1	0	0	0	0	0	0	0	0	0	0	0	0	0	0	0
K18336	0	0	0	0	0	0	0	0	0	0	1	0	0	0	0	0	0	0	0	0	0	0	0	0	0	0
K18365	0	0	0	0	1	0	0	0	0	0	0	0	0	0	0	0	0	0	0	0	0	0	0	0	0	0
K18366	0	0	0	0	1	0	0	0	0	0	0	0	0	0	0	0	0	0	0	0	0	0	0	0	0	0
K18383	0	0	0	0	1	0	0	0	0	0	0	0	0	0	0	0	0	0	0	0	0	0	0	0	0	0
K18425	0	1	1	0	0	0	0	0	0	1	0	0	0	0	0	0	0	0	0	0	0	0	0	0	0	0
K18429	0	0	0	0	0	0	0	1	0	0	0	0	0	0	0	0	0	0	0	0	0	0	0	0	0	0
K18430	0	0	0	0	0	0	0	1	0	0	0	0	0	0	0	0	0	0	0	0	0	0	0	0	0	0
K18431	0	0	0	0	0	0	0	1	0	0	0	0	0	0	1	0	0	0	0	0	0	0	0	0	0	0
K18456	0	0	1	0	0	0	0	0	0	1	0	0	0	0	0	0	0	0	0	0	0	0	0	0	0	0
K18540	0	0	0	0	0	0	0	0	0	0	1	0	0	0	0	0	0	0	0	0	0	0	0	0	0	0
K18622	0	0	0	0	0	0	0	0	0	0	0	0	0	0	1	0	0	1	0	0	0	0	0	0	0	0
K18829	0	0	0	1	0	0	0	0	0	0	0	0	0	0	0	0	0	0	0	0	0	0	0	0	0	0
K18831	0	0	0	0	0	1	0	0	0	0	0	0	0	0	1	0	0	0	0	0	0	0	0	0	0	0
K18850	1	1	1	1	1	1	1	1	1	1	1	1	1	1	1	1	1	1	1	1	1	1	1	1	1	1
K18893	1	1	1	1	1	1	1	1	1	1	1	1	1	1	1	1	1	1	1	1	2	1	1	1	1	1
K18900	0	0	0	0	0	0	0	0	0	0	0	0	0	0	0	0	0	0	0	0	0	0	0	0	0	1
K18918	0	0	1	1	1	1	1	1	2	1	0	0	1	2	0	1	0	4	3	0	0	0	0	4	4	2
K18923	1	1	1	0	0	1	0	0	1	1	0	0	0	1	0	1	0	1	1	1	0	1	1	1	1	0
K18926	0	0	0	0	0	0	0	0	1	0	0	0	0	0	0	0	0	0	0	0	1	0	0	1	1	0
K18939	0	0	0	0	0	0	0	0	1	0	0	0	0	0	0	0	0	0	0	0	1	0	0	1	1	0
K18979	1	1	1	1	1	1	1	1	1	1	1	1	1	1	1	1	1	1	1	1	1	1	1	1	1	1
K18997	0	0	0	1	0	0	0	1	0	0	0	0	0	0	1	0	0	1	1	0	1	1	1	0	0	0
K19058	0	0	0	0	0	1	0	0	0	0	0	0	0	0	1	0	0	0	0	0	0	1	0	0	0	0

K19076	0	0	0	1	0	0	0	0	0	0	0	0	0	1	0	0	0	0	0	0	0	0	0	0	0	0
K19117	0	0	0	1	0	1	1	0	0	0	0	0	0	0	1	0	0	0	0	0	0	0	0	0	0	0
K19118	0	0	0	1	0	1	1	0	0	0	0	0	0	0	1	0	0	0	0	0	0	0	0	0	0	0
K19119	0	0	0	1	0	1	1	0	0	0	0	0	0	0	1	0	0	0	0	0	0	0	0	0	0	0
K19127	0	0	0	1	0	0	0	0	0	1	0	0	0	0	1	1	0	0	0	1	0	0	0	0	0	0
K19128	0	0	0	1	0	0	0	0	0	1	0	0	0	0	1	1	0	0	0	1	0	0	0	0	0	0
K19129	0	0	0	1	0	0	0	0	0	1	0	0	0	0	1	1	0	0	0	1	0	0	0	0	0	0
K19130	0	0	0	1	0	0	0	0	0	1	0	0	0	0	1	1	0	0	0	1	0	0	0	0	0	0
K19141	0	0	0	1	0	0	0	0	0	0	0	0	0	0	0	0	0	0	0	0	0	0	0	0	0	0
K19142	0	0	0	1	0	0	0	0	0	0	0	0	0	1	0	0	0	0	0	0	0	0	0	0	0	0
K19145	0	0	0	1	0	0	0	0	0	0	0	0	0	1	0	0	0	0	0	0	0	0	0	0	0	0
K19147	1	1	2	1	1	1	2	2	1	1	1	1	2	2	1	1	1	2	1	1	2	2	2	0	1	1
K19157	0	0	0	0	0	0	1	1	0	0	0	0	0	0	0	0	0	2	1	0	0	0	0	2	2	1
K19158	0	0	1	0	2	1	0	0	2	0	1	1	1	1	0	0	1	0	0	1	0	1	1	2	2	2
K19159	0	0	1	0	2	2	0	0	2	1	1	1	1	1	0	0	1	0	0	1	0	1	1	2	2	2
K19166	0	0	0	0	0	1	0	0	0	0	0	0	0	0	1	0	0	0	0	0	0	0	0	0	0	0
K19171	0	0	0	0	0	0	0	0	0	0	0	0	0	0	0	0	1	0	0	0	0	0	0	0	0	0
K19218	0	0	0	0	0	0	0	0	0	0	0	0	0	0	0	0	0	0	0	1	0	0	0	0	0	0
K19225	0	0	0	0	0	0	0	0	0	0	0	0	1	1	0	1	0	0	0	1	0	0	0	0	0	0
K19267	1	0	0	0	0	0	0	0	0	0	1	0	0	0	0	0	0	0	0	0	0	0	0	0	0	0
K19302	1	0	2	1	2	1	2	2	0	1	0	0	0	0	0	0	0	0	0	0	2	0	0	0	0	2

Figure S3. Complete heat map of genes with KO number in the genomes of *Psychrobacter* species.
