# Supplementary material for: Isolation and genome sequencing of four Arctic marine Psychrobacter strains exhibiting multicopper oxidase activity
Source: BMC Genomics. 2016 Feb 16;17:117. doi: 10.1186/s12864-016-2445-4 (PMC4754876; doi:10.1186/s12864-016-2445-4)
Supplement: Additional file 3: Table S1. — Predicted amino acid residues forming substrate binding pocket. (PDF 87 kb) [file 12864_2016_2445_MOESM3_ESM.pdf]

Table S1. Predicted amino acid residues forming substrate binding pocket.

| Gene candidates | Amino acids residues |
|---|---|
| **P11F6-LMCO** | S41, A44, T45, I46, S48, Q50, N51, E97, L121, K150, Q151, S152, K178, G315, N316, D317, P415, R416, M417, N418, L419, D420, R490, G509, M510, W511, S512, D513, F521, Q522, R524, D539, V540, T541, G542, E543 |
| **P2G3-LMCO** | E96, L119, L120, V121, P122, F123, E124, D126, T146, K149, Q150, S151, G314, I315, D316, P414, M416, N424, R426, E465, R466, V482, I484, K485, P486, N487, R489, V490, I492, T493, M504, L506, G508, M509, W510, S511, L513, V522, R523, K524, H525, I527, F537, D538, V539, T540, G541, E542, A543, W546, W548, R562, E563, V564, V566 |
| **P11G3-LMCO1** | Q40, A43, V44, N45, D47, K48, A49, D50, I52, V53, P54, E95, H114, W115, H116, G117, L118, L119, D125, L147, V148, Q149, S150, K176, G313, N314, D315, A443, I444, I483, P485, R488, V489, I491, H504, L505, H506, G507, M508, W509, R522, H524, V538, T539, G540, E541, W545, V563, V565 |
| **P11G3-LMCO2** | Q40, A43, V44, N45, D47, S49, D50, H51, I52, V53, P54, F61, L91, M93, E95, T98, V99, I101, H114, W115, H116, G117, L118, L119, V120, P121, F122, E123, D125, K144, F145, K146, L147, K148, Q149, S150, G151, T152, Y153, I171, V172, I173, K176, G313 , I314 , D315, P413, N423, R425, E464, R465, V481, I483, P485, R488, V489, I491, T492, M503, H504, L505, H506, G507, M508, W509, S510, L512, V521, K523, H524, T525, I526, F536, D537, V538, T539, G540, E541, W545, W547, R561, E562, V563, V565 |
| **P11G5-LMCO1** | V44, I45, N46, D48, P50, E51, K53, V54, P55, E93, L120, L148, K149, Q150, S151, K177, R179, G314, I315, D316, M416, I484, P486, R489, V490, I492, G508, M509, W510, R523, V539, T540, G541, E542, W546, V564, V566 |
| **P11G5-LMCO2** | N40, V43, N44, S45, R47, A48, D49, H50, I51, E94, H113, W114, H115, G116, L117, L118, V119, M123, D124, F144, I147, Q148, S149, K175, G312, I313, D314, P412, M414, N415, L416, N422, R424, V480, I482, K483, P484, G485, R487, V488, I490, T491, M502, H503, L504, H505, G506, M507, W508, S509, D510, L511, Q519, V520, R521, K522, H523, T524, I525, F535, D536, V537, T538, G539, E540, A541, W544, W546, R560, E561, V562, V564 |
